# Nurses’ experiences of change fatigue during healthcare reforms: a qualitative study of psychological distress, impacts, and adaptation

**DOI:** 10.3389/fpsyt.2026.1878913

**Published:** 2026-07-22

**Authors:** Yana Wang, Rong Zhang, Xiaoyan Sheng, Shengyu Liu, Yunling Zhong, Jiehui Yang

**Affiliations:** 1 School of Nursing, Guangdong Pharmaceutical University, Guangzhou, Guangdong, China; 2 Hospital Office, Guangdong Second Rongjun Youfu Hospital, Foshan, Guangdong, China

**Keywords:** change fatigue, nurses, healthcare reform, psychological distress, adaptation, qualitative study

## Abstract

**Background:**

Global healthcare systems are undergoing continuous and rapid transformations aimed at improving service quality and efficiency. As frontline care providers, clinical nurses are directly exposed to frequent organizational changes. Change fatigue has emerged as a significant concern. However, most existing studies have employed quantitative approaches, offering limited insight into nurses’ subjective experiences of this phenomenon. This qualitative study, therefore, seeks to explore clinical nurses’ lived experiences of change fatigue and the coping strategies they adopt in response. The findings are expected to inform the optimization of reform implementation, facilitate targeted support for nursing staff, and promote nurse well-being and workforce stability.

**Methods:**

A qualitative descriptive study was adopted. Semi-structured, in-depth interviews were conducted with 17 clinical nurses between December 2025 and February 2026. Data were analyzed using Colaizzi’s seven-step method to progressively develop themes and subthemes. Participants were recruited via purposive sampling.

**Results:**

Four overarching themes with eight subthemes were identified: (i) Change-related physical and psychological experiences; (ii) Multiple triggering factors; (iii) Negative impacts of change fatigue; (iv) Coping strategies.

**Conclusion:**

Clinical nurses’ experiences of change fatigue are multidimensional and complex. Management should monitor nurses’ current levels of change fatigue, optimize the implementation process of reforms, strengthen support measures and nurses’ psychological resources, and promote the stability of the nursing workforce and the orderly progress of healthcare reforms.

## 1 Introduction

In recent years, the Chinese government has been committed to healthcare optimization reforms. With the implementation of the”Healthy China 2030”Plan, policies such as the tiered diagnosis and treatment system, Diagnosis-Related Group (DRG) payment reform, and smart hospital construction have been rolled out in earnest (1). Moreover, the increasingly evident trend of cross-disciplinary integration between medicine and other fields has ushered in the era of Modern Medicine Development 4.0 (2). In 2024, the State Council issued the “Key Tasks for Deepening the Healthcare Reform in 2024,” indicating that China will continue to advance its profound healthcare reforms. In 2025, China launched pilot programs for accompanied care exemption services, placing nurses in a leading care role. It is evident that nurses are undergoing significant changes. These reforms encompass several interrelated areas, such as the introduction of new nursing equipment; the expansion and relocation of hospital facilities; updates to health insurance reimbursement models; reforms to nursing performance evaluation; and the consolidation or reorganization of nursing units within wards, as well as staff reassignments (3, 4). As frontline implementers of these reforms, clinical nurses are required to frequently adapt to changes in work processes, management systems, operational protocols, and assessment methods, leaving them in a prolonged state of high-intensity adaptation. The concept of change fatigue has thus emerged.

The concept of change fatigue originated in management studies and was initially used to explore organizational change and its associated successes and failures (5). Chinese scholars have defined change fatigue as an inability to adapt to continuous change and as occupational burnout (6). However, the underlying mechanism of change fatigue differs from that of burnout. Change fatigue is triggered by “dynamic and continuous organizational change,” rather than by reaching the limits of tolerance for “routine workload.” Furthermore, while burnout focuses on “long-term stress accumulation,” change fatigue places greater emphasis on the notion that the “frequency and speed of change exceed an individual’s adaptive threshold (6). For clinical nurses, change fatigue is a condition in which they fail to adapt to persistent alterations in the clinical nursing work environment, leading to burnout, exhaustion, and a misalignment between their thoughts, behaviors, and the expectations of organizational leaders (7). As noted by Dilkers, when individuals frequently encounter organizational change, their capacity to cope with it and their enthusiasm for engaging with it gradually diminish (8). Sustained change-related stress leads to excessive depletion of psychological resources and energy reserves, thereby giving rise to change fatigue. This well explains the formation mechanism of change fatigue.

Research by several scholars has shown that nurses generally report moderate to high levels of change fatigue, which is significantly negatively correlated with job satisfaction and psychological resilience and positively correlated with perceived stress. The hospital scale has also been identified as a significant predictor of nurses’ change fatigue, with fatigue levels rising as the number of hospital beds increases (9–11). Change fatigue typically manifests in nurses’emotional changes or behavioral responses. In terms of emotional changes, nurses may experience feelings of apathy, withdrawal, stress, and moral dilemmas (12).In terms of behavioral responses, nurses often exhibit silent resistance to new initiatives and a reduced sense of organizational commitment (13), which leads to decreased life and job satisfaction (10). In the long term, this may increase nurses’ burnout and turnover rates. This has a series of negative impacts on nurses’ psychological and physical health, as well as on the development of the healthcare industry.

Currently, most studies on nurse change fatigue have adopted quantitative research methods. However, change fatigue is essentially a dynamic, context-embedded psychological process, the emergence of which is intertwined with multiple factors, including the frequency and intensity of change, communication styles, and organizational support. Quantitative research struggles to capture the meaning-making, emotional evolution, and flexible adjustment of coping strategies involved in this process. Therefore, relying solely on existing quantitative evidence makes it difficult to fully understand the nature of change fatigue and its profound implications for nursing practice and nurses’ health. Therefore, this study employs qualitative research methods to conduct semi-structured, in-depth interviews with clinical nurses, aiming to understand their real experiences and coping strategies in the context of ongoing healthcare reforms and to provide a basis for developing targeted intervention strategies and improving healthcare change management.

## 2 The study

### 2.1 Aim and objective

This qualitative descriptive study aims to explore the authentic experiences of change fatigue among clinical nurses amid ongoing healthcare system reforms and to examine the factors that influence it.

### 2.2 Research question

1. To explore the feelings and impacts on nursing practice that clinical nurses perceive as resulting from change fatigue during continuous healthcare reforms.

## 3 Methods

### 3.1 Design

A descriptive-phenomenological approach was adopted to explore the lived experiences and coping strategies related to change fatigue among clinical nurses in China. This approach facilitated an in-depth understanding of how participants perceived and interpreted change fatigue within clinical practice environments. Data were analyzed using Colaizzi’s seven-step method, which allowed for the condensation and interpretation of textual data to uncover subjective perceptions and contextual meanings.Colaizzi’s seven-step method was chosen because it operationalises descriptive phenomenological principles in a systematic, transparent manner-particularly its emphasis on extracting significant statements directly from participants’ words and returning the findings to participants for validation. The study reporting followed the Consolidated Criteria for Reporting Qualitative Research checklist (14). (The COREQ checklist have been provided as **Supplementary Material**, See **Appendix 2** for details).

### 3.2 Theoretical framework

This study was guided by the Cognitive Appraisal Theory of Stress (15) and the Conservation of Resources model (COR) (16). The Cognitive Appraisal Theory of Stress emphasizes that an individual’s primary and secondary appraisals provide a perspective on dynamic psychological reactions. The COR further explains individuals’ stress appraisal, their evaluation of stress and personal resources, and their coping assessment. According to the loss spiral hypothesis of COR theory, nurses with insufficient resources may perceive change as a threat, leading to resource depletion and, consequently, change fatigue. The specific application of these two theories in this study is reflected in the following three aspects:

#### 3.2.1 Guiding the design of the interview guide

Based on the Cognitive Appraisal Theory of Stress, questions were designed to explore how nurses perceive change events; based on COR, questions were developed to address resource consumption and compensation.

#### 3.2.2 Guide the coding process in data analysis

The two researchers adopted inductive coding as the primary approach, supplemented by deductive coding. During the initial coding phase, the researchers remained open-minded, extracting meaning units from the data while minimizing interference from theoretical presuppositions. In the theme integration and refinement phase, the preliminary themes were theoretically categorized in light of the study’s theoretical framework, thereby ensuring that the analytical results were comprehensive and scientifically rigorous.

#### 3.2.3 Guiding the interpretation of findings

In the discussion section, the two theories were used to explain the pathways of change fatigue. Together, the two theoretical models explain the impact of change-related stressors on individual behavioral outcomes, providing a complementary explanatory framework for understanding change fatigue. These two theories are highly consistent with the concept of change fatigue, jointly explaining nurses’ stress appraisal, resource appraisal, and the impact of change-related stressors on individual behavioral outcomes when facing organizational change, thereby providing a theoretical foundation for a systematic and in-depth investigation of change fatigue among the nursing population.

### 3.3 Study Participants

Using purposive sampling and the principle of maximum variation, clinical nurses from three tertiary general hospitals in Guangdong Province were recruited as subjects between December 2025 and February 2026. Attention was paid to gender, age, education level, marital status, professional title, and years of work experience to enhance the data’s representativeness. To ensure diversity, interviews were conducted across multiple departments-including internal medicine, surgery, psychiatry, emergency medicine, and intensive care-and involved participants with varying years of work experience and professional titles. The sample size was determined by data saturation. After completing each interview, the researcher conducted preliminary theme extraction and compared the findings with data from subsequent interviews. When no new themes or subthemes emerged after the 15th participant, data saturation was confirmed. To validate this determination, an additional two nurses were interviewed after the initial saturation point, and no new themes emerged. Therefore, the final sample size was set at 17 participants. In this study, 20 nurses were invited to participate. Twelve participated in face-to-face interviews, five completed online interviews via Tencent Meeting, and three declined due to concerns about the study’s confidentiality. A total of 17 nurses were ultimately interviewed and coded N1-N17 in the order of interviews. The online interview procedure was identical to that of the face-to-face interviews. The online participants chose to turn off their cameras, and the interviewer compensated for the lack of nonverbal information by attending to vocal tone and using follow-up probes. The two formats did not differ in terms of interview guide, duration, or transcription process.

### 3.4 Inclusion and exclusion criteria

Inclusion criteria (1): Holding a valid nursing practice license (2); Having worked in clinical nursing positions for ≥2 years (3); Having clear verbal expression and good comprehension ability (4); Voluntarily participating in this study. Exclusion criteria (1): previously diagnosed with a mental illness (2); on leave (maternity leave, sick leave, etc.) or away for advanced studies during the study period (3); confirmed pregnancy. General information about the participants is shown in **Table 1**.

**Table 1:** Demographic and clinical characteristics (N = 17).

Characteristic	n	%
Age		
18~30	12	70.6
31-45	5	29.4
Gender		
Female	14	82.4
Male	3	17.6
Working years		
≤5	10	58.8
6~10	6	35.3
≥11	1	5.9
Department		
Psychiatry	2	11.8
Traumatic Orthopedics	1	5.9
Neurology	1	5.9
Emergency	2	11.8
Internal Medicine	1	5.9
Oncologic Orthopedics	1	5.9
ICU	1	5.9
Cardiology	1	5.9
General Surgery	1	5.9
Rehabilitation	1	5.9
Operating Room	1	5.9
Neurosurgery	1	5.9
Biliary Tract Surgery	1	5.9
General Practice	1	5.9
Respiratory Medicine	1	5.9
Highest education level		
Bachelor’s degree	15	88.2
Master’s degree	2	11.8
Professional title		
Junior nurse	3	17.6
Intermediate nurse	13	76.5
Senior Nurse	1	5.9
Position		
Nursing Manager	4	23.5
Nurse	13	76.5
Marital and fertility status		
Married with children	2	11.8
Married without children	1	5.9
Unmarried and childless	14	82.4

### 3.5 Development of the Interview Outline

Based on the research objectives, an interview guide was developed through a literature review and team discussion. Two nurses who met the inclusion criteria were pilot-interviewed to revise and refine the guide, resulting in the final interview guide: (1) Among the hospital or work changes you have experienced, which changes have been most stressful or challenging for you? (2) In the process of coping with these changes, which of your own resources are more likely to contribute to physical and mental fatigue? (3) When you already feel change fatigue, how does this affect your attitude toward subsequent new changes? (4) How do you usually adapt to the impacts brought by changes? (5) What is your view on the stable period after changes? (The full interview transcripts have been provided as **Supplementary Material**, See **Appendix 1** for details).

### 3.6 Data collection

This was a phenomenological study that used one-on-one, semi-structured, in-depth interviews. The interviews were conducted by the first author, a full-time master’s degree student who had received systematic training in qualitative research and conducted pilot interviews with two clinical nurses prior to the formal study to refine questioning techniques. The interviewer had no working or personal relationship with any of the participants before the study. To mitigate potential power imbalances, participants were explicitly informed of the interviewer’s student identity prior to the interviews, and it was emphasized that the interview content would be used solely for academic analysis and was unrelated to any hospital evaluations. During the interviews, the researcher employed strategies such as listening, restating, and non-judgmental responses to minimize the interviewer’s background influence on data collection.Before the formal interviews, in accordance with the principles of voluntariness, informed consent, and confidentiality, the study’s purpose and methods were clearly explained to participants. After obtaining informed consent, the entire interview was audio-recorded. Interview locations and times were scheduled in advance with participants to ensure a quiet and undisturbed environment. During the interviews, facial expressions and body movements were observed and recorded. Techniques such as listening, restating, timely probing, and periodic summarisation were used to deeply explore participants’ real experiences and feelings. Each interview lasted approximately 25–40 minutes, with no strict time limit.

### 3.7 Data analysis

Data were analyzed using Colaizzi’s seven-step method to progressively develop themes and subthemes (17). Within 24 hours after each interview, the audio recordings were transcribed into text. Uncertainties were verified with the participants again. The transcribed text was imported into NVivo 15.0 software for organization and analysis. Non-verbal information presented by participants during the interviews was marked at the corresponding locations.

### 3.8 Ethical considerations

All study procedures were strictly conducted in accordance with the ethical principles outlined in the Declaration of Helsinki. This study was approved by the Ethics Committee of Guangdong Second Rongjun Youfu Hospital (Approval No. AF/SC-01/01.0-29). Both oral and written informed consent were obtained prior to all interviews, and only the research team had access to the digital audio recordings and transcribed texts.

### 3.9 Rigour and reflexivity

The study generated 410 minutes of audio recordings, which were transcribed into 83,763 words of effective text.To minimize potential bias and ensure research rigor,the following measures were taken (1): Researchers: researchers trained in qualitative research independently coded the data; disagreements were resolved through team discussion (2). Participant verification: To enhance the credibility of the study, we conducted participant verification. Transcripts were returned to participants for checking to ensure accuracy and the absence of bias (3). Disagreements were addressed through research team meetings (4). To control for potential bias arising from implicit shared understanding between the two coders, the research team reviewed the transcripts after the interviews and cross-validated the coding with authors who were not involved in the coding process. Throughout the interviews, the researcher maintained a neutral stance and encouraged participants to express their views authentically.

## 4 Results

### 4.1 Identified themes

We identified four overarching themes and eight subthemes. **Table 2** summarises all themes and provides illustrative quotations.

**Table 2:** Themes, subthemes, codegroups, and example quotes.

Theme	Subtheme	Codegroup	Examplequote
Theme 1: Change-related physical and psychological experiences	Emotional experiences	Resignation and frustration	When it comes to changes. I feel nothing but helplessness. But as frontline staff, we have no choice but to passively follow orders from above; we have no say in the matter.(N17)
		guilt toward colleagues	The change has already been implemented, and everyone else seems to know how to do it, not knowing makes you feel guilty about falling behind the team’s pace.(N10)
	physiological reactions	Anxiety and insomnia	This state becomes particularly strong when approaching work. For example, thinking about having to work the next day tonight makes me start to feel anxious, and the more I think, the more anxious I become, unable to sleep.(N2)
Theme 2: Multiple triggering factors	Organizational factors	Hasty implementation of reforms	Previously, we used traditional methods at a slower pace, but after the new system came we were required to master it completely in a very short time, which gave me a feeling of haste and discomfort.(N5)
		Lack of communication and feedback mechanisms	Previously, the hospital implemented a new nursing process change. The leader simply said there would be a change, but did not explain specifically how or to what extent due to poor communication, we didn’t know if we were doing it right, which made us anxious. Over time, fatigue set in.(N12)
		Insufficient rationality of reforms	There are many unreasonable work arrangements in the changes, such as a serious imbalance in workload between night shifts and day shifts, which makes me more tired and dissatisfied.(N10)
		Intensive and overlapping reforms	The frequency of changes is too high. Before one change is fully adapted, new changes follow one after another. It feels like constantly chasing a goal that can never be reached. The spirit is always in a state of high tension, and both body and mind become very tired.(N12)
	Personal factors	Inadequate personal capability	In recent years, I feel my ability has declined a bit… (wry smile) To be honest, I feel my adaptability is declining.(N13)
Theme 3: Negative impacts of change fatigue	Occupational consequences	Perceived increased workload	Although efficiency has improved for patients, the workload feels much heavier. One surgery that used to take over 40 minutes now has to be completed in 20, essentially doubling the workload.(N5)
		Passive work behavior	For routine work arrangements in the department, everyone shirks and passes the buck, avoiding work as much as possible. (N16)
		Develop thoughts of resignation	After being transferred to a new department the pressure of night shifts is so great that I have thoughts of resignation. (N2)
	Increased interpersonal conflicts	Emergence of conflicts and friction	I have a relatively impatient personality. If I feel someone is holding me back, I get very agitated. Colleague relationships also become tense.(N14)
Theme 4: Coping strategies	Organizational coping support	Clear reward and punishment mechanisms	I think if changes are to be made, corresponding reward systems should be established. You can’t just let us give without any positive incentives.(N15)
		Ensuring a period of sufficient stability	I hope that after a major change, there will be at least a period of time for everyone to adjust and adapt we can’t expect everyone to accept the change immediately.(N3)
		Increase emotional support	Hospital changes lack humanistic care for frontline nurses, focusing only on results while disregarding the process … They overlook the efforts and feelings of frontline nurses during the change process. We are overloaded with work, both physically and mentally exhausted, yet the hospital provides no compensation or care.(N16)
	Self-coping support	Self-regulation and family support	I can seek help from family members, because I think family is a very strong backing. I can tell them anything. Although they may not be able to help, the comfort they can give makes people feel warm.(N6)
		Self-directed skill capital accumulation.	The best way to prevent change fatigue is to have sufficient personal capability. Therefore, to better adapt to social changes, I usually take the initiative to learn new skills. (N8)

### 4.2 Theme 1: Change-related physical and psychological experiences

At the same time, several nurses expressed concern that continuous organizational change could be physically exhausting, emotionally draining, or difficult to reconcile with their personal well-being and clinical work. These perceptions were closely linked to the profound physical and psychological distress they encountered amid ongoing healthcare reforms.

#### 4.2.1 Emotional experiences

Rapid and continuous changes require nurses to constantly adjust their pace to meet work demands. As direct implementers of clinical changes, nurses face multiple challenges when responding to changes, leading to emotional experiences of resignation and frustration, as well as guilt toward colleagues. One nurse pointed out: “When it comes to changes. I feel nothing but helplessness. But as frontline staff, we have no choice but to passively follow orders from above; we have no say in the matter.”(N17). In addition, Nurse N10 reported, “The change has already been implemented, and everyone else seems to know how to do it. Not knowing makes you feel guilty about falling behind the team’s pace.”

#### 4.2.2 Physiological reactions

Some nurses pointed out that while experiencing emotional reactions to hospital reforms, they also developed physical reactions, such as anxiety and insomnia. These changes affected nurses’ emotional health and work engagement. For instance, Nurse N2 stated: “This state becomes particularly strong when approaching work.For example, thinking about having to work the next day tonight makes me feel anxious, and the more I think about it, the more anxious I become, unable to sleep.

### 4.3 Theme 2: Multiple triggering factors

#### 4.3.1 Organizational factors

Organizational factors are diverse, mainly reflected in the hasty implementation of reforms, a lack of communication and feedback mechanisms, insufficient rationality in reform implementation, and extensive overlap of changes. When facing these issues, nurses are more likely to fall into an unmanageable, overloaded state, with fatigue continuously accumulating.One nurse summarized: “Previously, we used traditional methods at a slower pace, but after the new system came we were required to master it completely in a very short time, which gave me a feeling of haste and discomfort.” (N5). One nurse declared: “Previously, the hospital implemented a new nursing process change. The leader simply said there would be a change, but did not explain specifically how or to what extent due to poor communication, we didn’t know if we were doing it right, which made us anxious. Over time, fatigue set in.”(N12).

Some nurses also indicated that certain reform measures lacked scientific design and implementation basis, were inconsistent with clinical realities, and that reforms were intensive and overlapped with one another: “There are many unreasonable work arrangements in the changes, such as a serious imbalance in workload between night shifts and day shifts, which makes me more tired and dissatisfied.” (N10). Another nurse pointed out that: “The frequency of changes is too high. Before one change is fully adapted, new changes follow one after another. It feels like constantly chasing a goal that can never be reached. The spirit is always in a state of high tension, and both body and mind become very tired. “(N12). These changes not only increased nurses’ workload but also easily triggered dissatisfaction and resistance, leaving nurses physically and mentally exhausted.

#### 4.3.2 Personal factors

When facing new pressures brought about by healthcare changes, nurses may feel helpless due to personal factors, such as age, work experience, and fear of making mistakes, leading to significant fatigue. Nurses reported that: “In recent years, I feel my ability has declined a bit… (wry smile) To be honest, I feel my adaptability is declining.”(N13).

### 4.4 Theme 3: Negative impacts of change fatigue

#### 4.4.1 Occupational consequences

Nurses reported that their clinical workload had already reached a saturation point. Following the implementation of reforms, adapting to new regulations, work environments, and requirements made the perceived increase in workload even more pronounced. Consequently, nurses’ professional motivation was inhibited, and passive work behaviors and burnout became common. One nurse pointed out: “Although efficiency has improved for patients, the workload feels much heavier. One surgery that used to take over 40 minutes now has to be completed in 20, essentially doubling the workload.”(N5). Another nurse pointed out that: “For routine work arrangements in the department, everyone shirks and passes the buck, avoiding work as much as possible.” (N16). Some nurses even developed thoughts of resignation: “After being transferred to a new department the pressure of night shifts is so high that I have thoughts of resignation.”(N2).

#### 4.4.2 Emergence of conflicts and friction

The physical and mental fatigue caused by changes accumulates negative emotions among nurses. These emotions not only extend to family life but also project onto workplace collaboration, creating relationship and atmosphere problems both at work and at home. One nurse confided in us: “My husband said I was like a ‘powder keg’ during that time of change. When my child accidentally knocked over a water cup, I couldn’t help but shout at him, and I felt very sorry afterwards.”(N11). Some nurses also experience friction with colleagues at work as a result. Another nurse pointed out that: “I have a relatively impatient personality. If I feel someone is holding me back, I get very agitated. Colleague relationships also become tense.”(N14).

### 4.5 Theme 4: Coping strategies

#### 4.5.1 Organizational coping support

Nurses hope that when facing change fatigue, the organisation can provide certain coping measures, including clear reward and punishment mechanisms and ensuring a period of sufficient stability, so that nurses can better cope with various pressures. Some nurses called for: “I think if changes are to be made, corresponding reward systems should be established. You can’t just let us give without any positive incentives.” (N15). Another nurse also called for: “I hope that after a major change, there will be at least a period of time for everyone to adjust and adapt we can’t expect everyone to accept the change immediately.”(N3).

In addition to structural and policy-level support, nurses also strongly called for managers to provide more emotional support during periods of change. Nurses expect hospital decision-makers to recognise the psychological pressure brought about by reforms, to listen to their concerns with empathy, and not to focus solely on outcomes while disregarding employees’ feelings. As one nurse stated:”Hospital changes lack humanistic care for frontline nurses, focusing only on results while disregarding the process. They overlook the efforts and feelings of frontline nurses during the change process. We are overloaded with work, both physically and mentally exhausted, yet the hospital provides no compensation or care.”(N16).

#### 4.5.2 Self-coping support

Meanwhile, nurses also employ various self-regulation methods to alleviate stress and anxiety.Some nurses indicated that family members’ understanding, care, and support are important mitigating factors, providing emotional security and psychological support as people cope with changes.The nurse explained, “I can seek help from family members, because I think family is a very strong backing.” I can tell them anything. Although they may not be able to help, the comfort they can give makes people feel warm.”(N6). Another nurse pointed out that: “On my way home from work every day, I listen to soothing music, take a hot bath at home, and sometimes do a 10−minute meditation to help relieve anxiety.”(N11).

Beyond self−regulation and family support, some nurses attributed their change fatigue primarily to insufficient personal capability—feeling unable to keep up with reforms, which led to exhaustion. Consequently, these nurses also demonstrated initiative in proactively accumulating self−skill capital to strengthen their adaptability, viewing this as a long−term strategy for coping with change. As one participant stated: “The best way to prevent change fatigue is to have sufficient personal capability. Therefore, to better adapt to social changes, I usually take the initiative to learn new skills. “(N8).

## 5 Discussion

In this qualitative study, we aimed to understand nurses’ authentic experiences of change fatigue, its influencing factors, and their coping strategies. Overall, all participating nurses reported experiencing some degree of change fatigue. The findings were qualitatively summarised into four overarching themes and eight subthemes: on the one hand, participating nurses were able to identify specific emotional experiences, physiological reactions induced by changes, and triggering factors of change fatigue; on the other hand, they also pointed out the impact of change fatigue on their work and offered certain suggestions to facilitate the implementation of reform measures. The results indicate that clinical nurses commonly experience symptoms of physical and mental exhaustion during the change process. These symptoms tend to appear before other negative perceptions, serving as early signals of change fatigue. Subsequently, they are accompanied by disappointment and negative feelings. This dynamic result is consistent with research on job demands-resources outcomes, which emphasizes timeliness and process (10, 18), suggesting that change fatigue is not merely an emotional reaction but a multidimensional state of physical and mental exhaustion. Multiple domestic and international studies have shown that nurses’ change fatigue is moderate to high (9–11, 19–21). This is consistent with the findings of the present study, in which all participants reported experiencing varying degrees of change fatigue. Furthermore, this study additionally found that the emergence of change fatigue is not merely a matter of individual emotional change; according to the COR theory applied in this study, it actually serves as an early warning signal of the continuous depletion of psychological resources. The COR theory posits that psychological capital is an important psychological resource for individuals facing adversity, helping them replenish their resources and reduce job burnout (22, 23). Organizational change increases nurses’ workload, which can easily lead to distracted attention, subsequently causing adverse events such as needlestick injuries and medication errors and ultimately negatively affecting turnover rates, work performance, and job satisfaction (24). This is consistent with the findings of the present study, in which participants reported that after experiencing change fatigue, they became passive in their work, some even developed thoughts of resignation, and their engagement in nursing work was compromised. According to Wang, change fatigue is significantly positively correlated with job burnout and indirectly affects nurses’ turnover intention through job burnout (25). This is consistent with the findings of quantitative studies that, when nurses cope with changes, their internal psychological resources significantly affect their change fatigue and turnover intention, as reported by related domestic and international scholars (26–29).

Furthermore, this study also found that different nurses adopt different coping strategies when facing change fatigue. Some nurses respond passively, while others actively seek all available resources to make psychological adjustments. We hypothesise that this is related to the nurses’ psychological resources.Therefore, nursing managers should focus on cultivating nurses’ psychological capital and enhancing their adaptability to change uncertainty through psychological resilience training, mindfulness interventions, emotional management courses, and other methods (30). International scholars, including Abdi et al., have shown that nurses’ reduced organisational commitment and increased emotional exhaustion, in turn, lead to more severe fatigue (31, 32). Based on participants’ accounts in this study, managers may lack sufficient practicality when implementing change initiatives, thereby reducing nurses’ organisational commitment and, in turn, contributing to change fatigue. This process constitutes a dynamic influence mechanism: managerial deficiencies lead to a decline in nurses’ organisational commitment, which in turn depletes psychological resources, thereby exacerbating change fatigue and ultimately leading to turnover. This process validates the applicability of the loss spiral hypothesis of the Conservation of Resources (COR) theory in the context of change fatigue among Chinese nurses. The pathway from managerial deficiencies to decreased organizational commitment, resource depletion, and ultimately turnover intention aligns closely with the theoretical predictions of COR theory. The findings of this study reveal mechanisms of action adapted to the real−world context of healthcare reform, providing reliable empirical evidence for implementing related practices in the nursing population. Furthermore, the findings also indicate that organizational managers should, before implementing changes, first evaluate their own leadership capacity, the completeness of the change planning mechanism, and the frequency of change implementation, so as to reduce the influence of factors external to the nurses themselves and thereby ensure a more scientific and reasonable change implementation process. In summary, this study’s findings have two important practical implications. First, organizational managers should monitor and identify nurses’ levels of change fatigue. Although this is a small-scale qualitative study, the results suggest that change fatigue may be a common phenomenon among nurses amid continuous reform. Previous cross-sectional survey studies have found that clinical nurses generally report moderate to high levels of change fatigue. However, it should be noted that not all nurses experience the same level of fatigue; some nurses in this study still demonstrated high psychological resilience. Second, alleviating change fatigue requires multi-level interventions: at the individual level, efforts can focus on enhancing nurses’psychological capital and active coping strategies; at the organisational level, change management can be optimised through measures such as providing stable periods, establishing feedback mechanisms, and implementing fair reward and punishment systems.

## 6 Strengths and limitations

This study innovatively constructs a multidimensional framework of change fatigue for clinical nurses, integrating physical and mental experiences, triggering factors, negative impacts, and coping strategies into a systematic analytical model.It enriches theoretical understanding of change fatigue in nursing and provides a new perspective on the dynamic development of nurses’adaptive pressure amid continuous healthcare reforms. However, this study has several limitations. First, voluntary participation may have introduced self-selection bias, meaning the sample may not fully represent the overall characteristics of the clinical nurse population, particularly those with high levels of change fatigue.Second, all participants were recruited from Guangdong Province, China, which may limit generalizability due to regional variations, and it is not possible to infer dynamic changes in fatigue levels over time. Third, although the study sample included four nurses in management positions, the overall sample was relatively young.This homogeneity may influence the themes that emerge; for example, a predominantly female sample may obscure the unique experiences of male nurses. Entry-level nurses may be more sensitive to peer evaluations, which could amplify themes such as “guilt” and “anxiety”; in contrast, senior nurses may be more concerned with the loss of professional autonomy. The intention to leave among entry-level nurses may not fully reflect the broader range of reasons for turnover observed among senior nurses. Furthermore, the high proportion of unmarried participants may have resulted in “family factors” contributing to change fatigue not being fully explored. Therefore, our findings are most directly applicable to young, early-career, and unmarried female nurses. Caution is warranted when generalizing these conclusions to senior nurses, managers, or married nurses. Future research should recruit a more diverse sample to validate the stability of our thematic framework across different nursing subpopulations.Finally, in accordance with ethical protection principles, this study excluded nurses with a history of mental illness and those who were pregnant. This means that the findings cannot be generalised to these two groups. However, However, given that change fatigue may be more pronounced or manifest differently in these populations, future research should develop ethical protocols to safely include nurses with a history of mental illness and those who are pregnant. Such protocols should include: (1) pre-enrolment psychological risk assessment; (2) clear crisis intervention contingency plans; (3) enhanced informed consent procedures that explicitly describe the nature and scope of potential risks; and (4) the involvement of qualified mental health professionals to monitor participants’ well−being throughout the study. Admittedly, the above protocols may not be exhaustive, which also points to new directions for subsequent research. Future studies should, under the premise of well−established psychological support systems and emergency protocols, conduct relevant investigations in these special populations to generate more comprehensive evidence. And the findings mainly reflect perspectives in the context of ongoing healthcare reform in China. And their applicability in other settings remains to be verified.

## 7 Conclusion

This study found that change fatigue among clinical nurses during healthcare reforms is a multidimensional phenomenon, manifested as physical and psychological distress and professional alienation, and leading to serious consequences, including turnover intentions and interpersonal conflicts. Guided by the Cognitive Appraisal Theory and the Conservation of Resources model, this study provides context-specific empirical support for the pathway from resource threat and emotional exhaustion to eventual adverse outcomes and elucidates the underlying mechanism by which poorly managed organizational changes continuously deplete nurses’ psychological resources.

The findings of this study can be used to develop targeted interventions, including early fatigue screening, establishing a stable adaptation period, and responsive feedback mechanisms, thereby stabilizing the nursing workforce and providing a reference for advancing healthcare reforms in different contexts and populations. It should be noted, however, that the applicability of various interventions needs to be assessed on an individual basis, and the types of support required by nurses at different career stages may vary.Given that the sample in this study was predominantly young females, the results may be subject to some degree of homogeneity. Therefore, future research with more diverse samples is needed to further enhance the generalizability of the findings.

## Data Availability

The original contributions presented in the study are included in the article/**Supplementary Material**. Further inquiries can be directed to the corresponding author.
