# Supplementary material for: Nurses’ experiences of change fatigue during healthcare reforms: a qualitative study of psychological distress, impacts, and adaptation
Source: Front Psychiatry. 2026 Jul 22;17:1878913. doi: 10.3389/fpsyt.2026.1878913 (PMC13437614; doi:10.3389/fpsyt.2026.1878913)
Supplement: Supplementary file 1 [file DataSheet1.pdf]

Consolidated criteria for reporting qualitative studies (COREQ): 32-item checklist

From:*Nurses' Experiences of Change Fatigue During Healthcare Reforms: A Qualitative Study of Psychological Distress, Impacts, and Adaptation*

Please indicate in which section each item has been reported in your manuscript. If you do not feel an item applies to your manuscript, please enter N/A.

No.	Item	Guide questions/Description	Section #
**Domain 1: Research team and reflexivity**			
Personal characteristics			
1.	Interviewer/facilitator	Which author/s conducted the interview or focus group?	3.6 Data collection
2.	Credentials	What were the researcher's credentials? *E.g. PhD, MD*	3.6 Data collection
3.	Occupation	What was their occupation at the time of the study?	3.6 Data collection
4.	Gender	Was the researcher male or female?	N/A
5.	Experience and training	What experience or training did the researcher have?	3.6 Data collection
Relationship with participants			
6.	Relationship established	Was a relationship established prior to study commencement?	3.6 Data collection
7.	Participant knowledge of the interviewer	What did the participants know about the researcher? *E.g. Personal goals, reasons for doing the research*	3.6 Data collection
8.	Interviewer characteristics	What characteristics were reported about the interviewer/facilitator? *E.g. Bias, assumptions, reasons and interests in the research topic*	3.6 Data collection&<br>3.9 Rigour and reflexivity
**Domain 2: Study design**			
Theoretical framework			
9.	Methodological orientation and theory	What methodological orientation was stated to underpin the study? *E.g. grounded theory, discourse analysis, ethnography, phenomenology, content analysis*	3.1 Design
Participant selection			

10.	Sampling	How were participants selected? *E.g. purposive, convenience, consecutive, snowball*	3.3 Study Participants
11.	Method of approach	How were participants approached? *E.g., face-to-face, telephone, mail, email*	3.3 Study Participants
12.	Sample size	How many participants were in the study?	3.3 Study Participants
13.	Non-participation	How many people refused to participate or dropped out? What were the reasons for this?	3.3 Study Participants
Setting			
14.	Setting of data collection	Where was the data collected? *E.g. home, clinic, workplace*	3.6 Data collection
15.	Presence of nonparticipants	Was anyone else present besides the participants and researchers?	3.6 Data collection
16.	Description of sample	What are the important characteristics of the sample? *E.g. demographic data, date*	3.3 Study Participants&Table 1
Data collection			
17.	Interview guide	Were questions, prompts, guides provided by the authors? Was it pilot tested?	3.5 Development of the Interview Outline
18.	Repeat interviews	Were repeat interviews carried out? If yes, how many?	N/A
19.	Audio/visual recording	Did the research use audio or visual recording to collect the data?	3.6 Data collection
20.	Field notes	Were field notes made during and/or after the interview or focus group?	3.7 Data analysis
21.	Duration	What was the duration of the interviews or focus group?	3.6 Data collection
22.	Data saturation	Was data saturation discussed?	3.3 Study Participants
23.	Transcripts returned	Were transcripts returned to participants for comment and/or correction?	3.7 Data analysis&3.9 Rigour and reflexivity
**Domain 3: analysis and findings**			
Data analysis			

24.	Number of data coders	How many data coders coded the data?	3.9 Rigour and reflexivity
25.	Description of the coding tree	Did authors provide a description of the coding tree?	4.1 Identified themes& Table 2
26.	Derivation of themes	Were themes identified in advance or derived from the data?	3.2 Theoretical framework
27.	Software	What software, if applicable, was used to manage the data?	3.7 Data analysis
28.	Participant checking	Did participants provide feedback on the findings?	3.9 Rigour and reflexivity
Reporting			
29.	Quotations presented	Were participant quotations presented to illustrate the themes / findings? Was each quotation identified? *E.g. Participant number*	4 Results&Table 2
30.	Data and findings consistent	Was there consistency between the data presented and the findings?	Relationship to existing knowledge
31.	Clarity of major themes	Were major themes clearly presented in the findings?	4 Results
32.	Clarity of minor themes	Is there a description of diverse cases or discussion of minor themes?	4 Results
