# Supplementary material for: Nurses’ experiences of change fatigue during healthcare reforms: a qualitative study of psychological distress, impacts, and adaptation
Source: Front Psychiatry. 2026 Jul 22;17:1878913. doi: 10.3389/fpsyt.2026.1878913 (PMC13437614; doi:10.3389/fpsyt.2026.1878913)
Supplement: Supplementary file 2 [file Table1.docx]

Interview Transcript (N1)

Q: What types of changes have you experienced in your clinical work? For example, process optimizations, technology updates, adjustments in management models, etc. Please describe one or two events that left a deep impression on you.

A:I transitioned from a private outsourcing company to a hospital with formal management. The management had to align with the hospital's standards. However, since I have never been transferred to another department—I have always stayed in this psychiatric department—the changes for me have mainly been about moving from one employer to another. Previously I worked for a private organization, and now I have transferred to a formal hospital. Although the work model is still what we used to do, private management had different requirements—for example, we had to attend certain professional training sessions or assessments, but previously those demands were not as high. After aligning with the hospital, this change felt easy to accept given my professional background. The only issue is that I might not perform as well as the hospital's standards require.

Q: So the difference between your previous and current situation is that the work model is more formal and more detailed. Previously, due to the nature of the employer, management was outsourced, so it may have been less formal. After being acquired by the hospital, things became more meticulous and standardized.

A: Also, the hospital now sends us for training, academic conferences, lectures—things like that. Our previous employer did not place much emphasis on these. Since I have not been transferred to another department, I have not felt much internal turmoil regarding work changes. The model remains the same as our original psychiatric department model, so the work pattern hasn't changed much. Even after the hospital took over, many things still rely on those of us who stayed to gradually train our colleagues. The work model did not change overnight by transferring everyone away. Therefore, this change has not caused me much work pressure.

Q: So you can accept it, especially since you have been working in this department since the beginning.

A: Yes, always in this department. But before, we used paper medical records. Later, after coming here, we had to switch to electronic records—there was a transition period. We used electronic documentation for a while. It seems we changed systems twice; the first system was not very complete, then we had two more revisions, and now we have this current system. Recently I have been reflecting: I am 44 years old now, and I feel that with age, I cannot keep up as quickly with electronic and external knowledge. I can clearly feel that I am slower to accept new things, and it is somewhat exhausting. Previously at work, I learned things quickly and reacted fast. Now I gradually notice that, especially with the new system we are using, I learn more slowly, and my ability to absorb new information may be worse. So work feels more tiring, and I learn things more slowly. I need to jot down notes from time to time about what I need to do. With the current system, I feel that work pressure is greater than before. Coupled with my age, my ability to accept new things has declined, and what I need to learn now is different from before—I feel somewhat overwhelmed.

Q: So regarding changes, the two that impressed you most are: first, the update in department management (from outsourcing to hospital takeover); second, the update of the electronic information system, which you found difficult to adapt to quickly.

A: Yes.

Q: At the time, when information about these changes—whether the management model, electronic system update, or others—was conveyed to you, what was your first reaction? What did you feel?

A: I thought to myself: times are changing so fast, too fast, and sometimes I feel like I cannot keep up.I sometimes feel anxious, feeling that I am old and learn things a bit slower, which makes me a bit anxious.

Q: Given your age but also your many years of experience, how do you assess your ability to cope with these changes?

A: In terms of psychiatric work experience, since I have always been in psychiatry, I might be weaker in emergency care, but my ability to observe patients' mental status and related aspects is fairly good.

Q: So you mean it depends on the type of change. If the change involves more detailed professional skills within your area of expertise, you feel you have some ability to cope. But if the change is in an area you are not good at, you may feel helpless.

A: Yes, I would feel unable to cope. For example, internal medicine is my weak point. In psychiatric observation, I think I can manage.

Q: So your assessment of your ability to cope with changes is mainly based on the magnitude and type of the change.

A: Yes, and also my own work experience.

Q: When changes lead to increased work difficulty or adjustments in work content, what are your inner thoughts?

A: I definitely think I need to observe more and learn more. If you don't learn, you will be eliminated. So now, because I easily forget things, I write them down in a notebook. I have to keep up with the pace of change. Even though I worry that I might not be able to accept it, I still have to learn.

Q: So you have a positive attitude toward change.

A: Yes, positive. I wouldn't say I oppose it. But the progress of society demands this. Opposing won't help. I will learn as much as I can.

Q: In the process of coping with changes, do your personal resources—such as work enthusiasm and emotional energy—get affected?

A: Definitely they do. But on the other hand, this is our work environment. If we all think about not working, who will do the work? Since we have chosen this job, we must be worthy of our salary.

Q: In the process of coping with changes, how do you feel your resources, like your energy or work enthusiasm, are affected? Do they decrease, or does learning more increase your enthusiasm?

A: Excessive changes can cause psychological pressure, yes. But given the tough environment around us, if we don't work, we cannot survive.

Q: So, regarding the impact on your resources when coping with changes, can we understand that it is relatively diminishing? That is, too many changes too quickly can reduce your work enthusiasm—could there be negative effects?

A: Negative effects definitely exist, but we still have to work. Human emotions fluctuate. But when I think about my family and my life, I have to continue working.

Q: So external factors leave you with not many choices?

A: Yes.

Q: When facing these work changes, what methods do you usually use to cope?

A: Due to family or work reasons, when I feel anxious at work, I write—I practice calligraphy or listen to music to relax. I also go out with friends for tea, talk things out, share my work worries. That way we resonate with each other, and I feel better mentally. Actually, it's not that I dislike too much work or find it tiring. Work is work; you have to do your job.

Q: Did you figure out these methods on your own, or does the hospital provide regular support, such as recreational activities?

A: The hospital also provides activities that allow us to go out and relax, which is quite good. Nowadays we have outings. As a Party member, I participate in some rural free clinic activities, which let me see new things and also help me relax.

Q: So you feel that both the methods you discovered yourself and those provided by the hospital are quite effective.

A: Yes, effective. Sometimes you have to rely on yourself to relax your body and mind. I can also talk to my family. Going out with friends can also be relaxing; we are all in the same profession and can confide in each other. Chatting together and talking about work makes me feel much better. Coping with changes is similar.

Q: What thoughts come to your mind when the hospital introduces frequent changes?

A: If changes are too frequent, I will definitely feel anxious, and I may develop career worries. It mainly depends on the nature and direction of the change. If the change is in an area of my strength, then I'm not afraid—I can do better and better. But if it's in a weak area, I have to think about where to start.

Q: So the thoughts that arise are mainly based on the nature and method of the change.

A: Yes.

Q: If it is a positive change, then your thoughts are relatively positive as well.

A: Yes, positive.

Q: For example, our hospital recently went through the tertiary review. How did you feel about that?

A: After the tertiary review, our hospital standards certainly cannot be the same as before; they will be stricter. So at work, requirements may become more stringent, and I need to be stricter with myself to keep up with the tertiary standards. So there is work pressure.

Q: What mindset do you use to cope with this pressure?

A: Do as much as I can, do my best. I still need guidance from leaders on what to do and which direction is better.

Q: During the process of hospital changes, how do they affect your work behavior and work outcomes?

A: Everything becomes more detailed, more meticulous, and many aspects must be standardized.

Q: What is your view on such requirements?

A: It's for the good of the hospital, but we still need to see how they are implemented.

Q: If changes become too frequent and you find them hard to adapt to, what physical and mental reactions do you experience?

A: If I cannot adapt, I become anxious.

Q: What are your specific manifestations?

A: I feel like I cannot cope, and I worry that I might not do well or cannot manage.

Q: In the context of hospital changes, do you hope for a stable period?

A: A stable period would be best, so I can transition gradually. But the duration of this transition depends on the environment. Times are changing—does the stable period have a fixed length?

Q: If there were a fixed period, how long would you hope it to be?

A: That is hard for me to estimate.

Q: So it really depends on the specific type of change and the scale of the event, right?

A: Yes. Because our department is like a team. At work, we rely on mutual cooperation. If we cannot work together, it would be even harder to adapt. Team collaboration is very important. If everyone collaborates well, the stable period can be shorter. We would finish work happily together.

Q: Is there anything else you would like to add?

A: Since I have never been to other departments—I have always been in psychiatry, for 20 years now—I am quite happy working in this unit. I have never thought about jumping to another hospital. In my department, I always feel that our team collaboration is very good. Many of us have been working together for over ten years, which is rare. The colleagues who left before did so because of family reasons, not because they wanted to leave the hospital. Most of my colleagues have settled down here, started families, and have no intention of leaving. We have heard that other hospitals pay more for night shifts—for example, we used to get only 30 yuan per night shift—but we never complained about the hospital's compensation, because our team collaboration is very good and we work smoothly. So no one wanted to leave. Personally, after experiencing a change, I definitely hope for an adaptation period. Once I become familiar with the work and it goes smoothly, the pressure will decrease. I can cope, and I won't experience symptoms like anxiety or insomnia.

Q: So what you mean is that the changes you experienced were not things like department transfers, but rather changes stemming from hospital development. Change fatigue arises mainly from a lack of personal capability. If you have sufficient capability, change fatigue will be smaller; if your capability is insufficient, you will feel more anxious, and when coping with changes, you are more likely to have negative reactions, leading to fatigue.

A: The reason for negative reactions is also that I want to do better. Facing these changes, my main worry is that I might not do well or cannot manage. For example, now that my memory is worse, I need to write things down in a notebook. I don't understand electronic procedures very well, so sometimes I need to ask colleagues about the steps.

Q: That concludes our interview for today. Thank you very much for your cooperation.
