# Supplementary material for: Nurses’ experiences of change fatigue during healthcare reforms: a qualitative study of psychological distress, impacts, and adaptation
Source: Front Psychiatry. 2026 Jul 22;17:1878913. doi: 10.3389/fpsyt.2026.1878913 (PMC13437614; doi:10.3389/fpsyt.2026.1878913)
Supplement: Supplementary file 3 [file Table10.docx]

Interview Transcript (N10)

Q: Please describe one or two change events that left a deep impression on you, in which you were highly involved or that made you feel pressured or challenged.

A: When I first joined, I was a rotating nurse - I had to rotate through departments. Every six months I would be transferred to a new department. I joined in 2022, and just three months after joining, the COVID-19 restrictions were fully lifted. At that time, as a rotating nurse, I had to go wherever there was a shortage of staff. The hospital sent me to the ICU. That was really stressful for me, because I knew nothing -I had only been there three months and was still very inexperienced. Suddenly being sent to the ICU -that was really high pressure. That was the most stressful thing for me. Other than that, I was fine, because my adaptability is quite good, and the departments I rotated to later were relatively better.

Q: When you first heard that you were being rotated to the ICU, what was your immediate reaction?

A: Actually, my first reaction was happiness.

Q: Why were you happy if it was stressful?

A: Because before that I was in neurosurgery, which I hated even more. In neurosurgery, not only was the work intensity high, but the patients' conditions were complex, and my nerves were constantly tight. Some patients were very difficult, and their families had many demands, making things particularly troublesome. In comparison, although being transferred to the ICU was stressful and there was a lot I didn't know, it also meant a new challenge and opportunity. The atmosphere in neurosurgery was bad, and the leaders were very strict-I really wanted to escape neurosurgery. So although going to the ICU was stressful, I was still looking forward to it. I thought I could learn new professional knowledge and skills that I had never been exposed to before, and improve my work ability. I also felt it was a sign of the hospital's trust in me-even though I had only been there three months, sending me to the ICU was a recognition of my ability. At first, I was indeed flustered. Facing all kinds of advanced medical equipment and complex conditions, I felt like a headless fly. But escaping neurosurgery-I was really happy inside.

Q: As a rotating nurse, how long did you have to rotate for?

A: Previously, it was usually three years. But now, because the turnover rate is very high-especially in our hospital-some people are assigned to fixed departments early, so they might not have to do the full three years of rotation. But the departments they are fixed to are all the bad ones.

Q: If you don't want to be fixed to a particular department, can you continue as a rotating nurse?

A: That's unlikely. The hospital doesn't really ask for your opinion. A friend of mine was fixed to the respiratory department. He said he didn't want to be fixed, but the nursing department said no-you must be fixed. They gave him two choices: emergency or respiratory. So it looked like a choice, but it was actually aforced assignment, because basically no one chooses emergency.

Q: Can you describe, when you were rotating in the ICU and first arrived at the new department, what changes made you feel fatigued?

A: Actually,On my first day reporting to the new department, I knew nothing, and the department was a mess. At that time, I felt I couldn't take it anymore. When I got home from work, I really didn't want to do it anymore and wanted to quit. Because on the first day I was paired with a teacher, but that teacher was also new-he had never worked in the ICU before; he had been transferred from the cardiac surgery ward. It was also his first day, but because there weren't enough preceptors, he became my preceptor. But he also knew nothing. On the first day, we admitted a patient whose oxygen saturation was only 67%. That patient died the same day. I thought, oh my god, this job is so tiring, I don't want to do it anymore. That night we worked until 3 am, and I didn't get off until after 6 am. In terms of factors, the main thing was the change in work environment, which led to a change in patient conditions. Previously, neurosurgery patients generally did not have many acute emergencies, but in the ICU, every patient was critically ill. I really felt a sense of helplessness, and I was very tired.

Q: At that time, how would you rate your mastery of ICU professional knowledge?

A: I knew nothing at all.

Q: How many years of experience did your preceptor have?

A: Probably only three to five years.

Q: So he was also a relatively junior nurse, and he was assigned to guide you-a rotating nurse with only three months of experience-both of you facing a new environment, new patients, new diseases.

A: Yes, he was also very young. There was also a factor of change in the preceptor. In neurosurgery, my preceptors had over 20 years of experience. In the ICU, the preceptor had no experience either. If it had been a senior nurse, they could have handled the situation easily. But with junior nurses, he couldn't handle it, I couldn't handle it, and he couldn't really guide me. So that day when I got home, I really felt I couldn't do this job.

Q: After you had been in that department for a while, how did your feelings change?

A: Not much change-I just slacked off. The next day, the teacher who was guiding me disappeared. He went back to his own department because he also felt too much pressure. Later, another teacher replaced him.

Q: So your preceptor on the first day and on the second day were different.

A: Yes. The situation was very chaotic at the time-everyone was like that. On the first day, many people didn't know what was happening. Some places were still under lockdown. By the second day, the lockdown was lifted, and many teachers who had been-confined at home- could return to work. So the teacher I had on the second day was more experienced. Then I felt that the job was actually okay,because I disliked neurosurgery even more; the atmosphere there was terrible.

Q: In the process of coping with these changes, what personal resources do you think were more likely to cause you fatigue,for example, time and energy, professional skills, interpersonal relationships, work autonomy, professional identity, etc.?

A: I would say professional skills. After going to the ICU, there were many machines and devices, and there were many things I didn't know how to operate. Also, the work process was different. In the neurosurgery ward, everything was well coordinated for example, if we were about to admit a patient, someone would have already made the bed and prepared the nebuliser. But in the ICU, because of the environment and the special nature of the department, no one helped you with those tasks. You had to prepare the bed and supplies yourself. Then, professional skills because I didn't know those things, I felt doubly pressured. I wasn't familiar with the environment either- sometimes I had to look for various items. Every department stores things in different places. When you first arrive, you don't know where things are, so you have to search frantically, and sometimes you search for a long time and still can't find them.

Q: Did your preceptor give you any guidance?

A: Actually, when you are actually working, you feel embarrassed to ask someone to find things for you, and you feel embarrassed to repeatedly ask for help. Because the change has already been implemented, and everyone else seems to know how to do it, not knowing makes you feel guilty about falling behind the team's pace.You’re too embarrassed to admit what you don’t know, worried you’ll seem stupid and get judged.

Q: Later, which department were you fixed to?

A: I was fixed to cardiology. I had already been to more than ten departments. In the end, I chose cardiology.

Q: So your work environment has been constantly changing.

A: Yes.

Q: In the process of coping with these changes, what thoughts arise?

A: Actually, I don't have any particular thoughts anymore. My attitude towards any hospital arrangement or change is: whatever, I don't care. The main thing is that the shift schedules and work content are different in each department, and actually I look forward to that. Each department has different work requirements. For example, in the first department, certain tasks were required to be done on night shifts; in the second department, the same tasks might be assigned to the day shift. So that is a change in work environment and content.

Q: So you have to constantly adapt. What are your inner thoughts?

A: I feel annoyed, but I don't feel anxious. Because I think some departments are actually right-some tasks really are unreasonable to assign to night shifts. If a task is given to the day shift, there are more people and it might take only five minutes, but if given to the night shift, it might take thirty or forty minutes. But I don't really feel anxiety-if I am asked to work, I can work. I just feel really speechless. Sometimes, in some departments, day shifts are very idle while night shifts are very busy. So when rotating, if the previous department had unreasonable work arrangements, I do look forward to whether the next department might be better.

Because I can't change anything myself, I just have to accept the hospital's arrangements. My mentality is to slack off.My mindset is to slack off. I actually hate being a nurse-even now.

Q: Was that your mindset when you just graduated?

A: When I just graduated, I was very motivated.

Q: Then why did you become like this?

A: (Laughs) I was influenced by a senior. I had a good relationship with a head nurse. When I was rotated to a really bad department, he told me: "Don't perform too well here. If you perform too well, you will be fixed to this department." So I slacked off like crazy. Later, I found that slacking off felt great.

Q: So when you just graduated, you were not that resistant to nursing as a profession.

A: I really liked being a nurse at the beginning. I have strong communication skills,I have no problem communicating with patients, and I really enjoyed interacting with them.

Q: So your mindset changed over time, influenced by colleagues or certain events?

A: Actually, even without that senior, I would have slacked off anyway.

Q: Why is that?

A: (Sigh) We feel that our current pay is not proportional to our efforts. I have no say in the hospital's decisions, so I just want to slack off, just go through the motions.

Q: When you are already feeling change fatigue, how does that affect your view of subsequent new changes?

A: Actually, not much effect. Sometimes when I am in the midst of environmental changes, I still look forward to it. When I rotate to a new department, there is a fresh feeling at the beginning, and it is acceptable. Later, when I find unreasonable things in the department and can't accept them, I actually prefer to have a new change. For example, if I am rotating in Department A and after two months I really hate it, then in the fourth month the nursing department tells me to go to a new department. That new department is actually another change for me, but I am quite willing to go to a new changing environment, because a new setting actually improves my mood,I was getting very annoyed in the previous department.

Q: So you actually want some freshness in your work.

A: Yes.

Q: With a relatively slacking‑off mindset, how does that affect the quality of your implementation of new changes required by the leaders?

A: Actually, the leaders don't ask much of me now. They have communicated with me before. I told them: "I think whatever you say to me, I will forget as soon as I walk out the door." So now the leaders don't come to me for anything.

Q: So you have communicated with the leaders that you don't want to deal with these things.

A: Yes. I told him that, so now he basically doesn't come to me for anything. There was a change this year,they said we needed to make PowerPoints for ward rounds for patients hospitalised for more than a month. But because I told him that I don't know how to do any of that, he hasn't come to me about it this year. I don't want to deal with it either.

Q: When you feel fatigue or pressure from changes at work, how do you usually relieve it?

A: Go out drinking at night. I'm a guy those are the usual ways to relax.

Q: Does the hospital offer any psychological courses or relevant educational sessions to relieve nurses' physical and mental issues?

A: The hospital just makes us do some questionnaires. There are no real intervention activities. Probably just for writing papers. There are no real measures. In university, there were psychological surveys, and if your score was really bad, someone would contact you to provide psychological support. But in the hospital, there is nothing.

Q: During changes, how do you view a stable period?

A: Let me think… Actually, I don't think it matters whether there is a stable period or not. I won't actively complete things, and I won't actively embrace changes. But I do hope for some new changes. Because if you already hate a certain change, and the things that change bring are also annoying, then you hope for a new change. I probably won't last much longer in this job so tired, every day is so tired.

Q: So you feel that work pressure is high and you are very tired?

A: Work pressure is part of it, but mainly I really hate night shifts.

Q: What factors do you think could alleviate change fatigue?

A: For me, actually, when I go to a new environment and get rid of the old one, my work enthusiasm on the contrary increases.

Q: What factors do you think would further aggravate change fatigue?

A:I feel that if there are additional tasks but no compensation measures, I will feel very annoyed, and my fatigue will further increase. For example, if the hospital suddenly arranges some temporary overtime tasks, or requires us to attend various trainings and exams, but without corresponding time off, bonuses, or other forms of compensation. Also, if a new change only increases our workload without bringing actual benefits like improving work efficiency or the work environment that also makes me especially tired and fed up. There are many unreasonable work arrangements in the changes, such as a serious imbalance in workload between night shifts and day shifts, which makes me more tired and dissatisfied. Anyway, I hope that when the hospital makes changes, they would consider the actual situation of us nurses more, and give us more support and compensation-that might make us more accepting of the changes.

Q: Regarding nurse change fatigue, is there anything else you would like to add?

A: Nothing else to add. I feel that after being a nurse for a long time, my brain has become dull. My memory isn't as coherent as before.

Q: Thank you for your cooperation.
