# Supplementary material for: Nurses’ experiences of change fatigue during healthcare reforms: a qualitative study of psychological distress, impacts, and adaptation
Source: Front Psychiatry. 2026 Jul 22;17:1878913. doi: 10.3389/fpsyt.2026.1878913 (PMC13437614; doi:10.3389/fpsyt.2026.1878913)
Supplement: Supplementary file 4 [file Table11.docx]

Interview Transcript (N11)

Q: Please describe one or two change events in which you were highly involved, that left a deep impression on you, or that felt challenging and stressful.

A: There are too many. For example, last year's "Enhanced Recovery After Surgery (ERAS) nursing pathway standardisation" and the launch of the "Nursing Quality Sensitive Indicator Real-time Monitoring System" earlier this year. Infection prevention and control procedures have been adjusted at least three times in the past two years-especially the one at the end of 2023, which changed even the visiting policy and disinfection frequency. That had a big impact on our surgical department.

Q: With so many changes pushed forward intensively, that must have been hard. Which of these had the greatest impact on your daily work? Could you describe your most direct feelings during the process?

A: The biggest impacts were the campus integration, the electronic medical record (EMR) upgrade, and the ERAS pathway standardisation. They all happened between the second half of 2022 and the first half of 2023-almost overlapping. During the campus integration, our surgical department had to merge the two wards from the East Campus and South Campus into the surgical building of the main campus. As a teaching team leader, I first had to go with the head nurse to the main campus to coordinate the layout and bed allocation of the new ward, and also inventory patient information, medical equipment, and supplies from the two original wards. Just checking patient records and transfer risk assessments took a week of continuous overtime. Then came the EMR upgrade. The hospital required everyone to become proficient within three months. Not only did I have to attend weekend intensive training myself, but I was also responsible for one‑on‑one teaching of 12 junior nurses in the department. We stayed one hour after work every day to practice, and I had to correct their EMR homework. During that period, I didn't get home until after 9 pm every day. The ERAS pathway standardisation was even more complex – I had to memorise dozens of nursing procedures, such as preoperative fasting times and postoperative pain management steps, and coordinate with doctors, anaesthesiologists, and rehabilitation therapists. I also had to fill out specialised ERAS assessment forms every day, which added a lot of paperwork.

Q: With so many changes piling up, what was your first reaction when you received the notice? Did that attitude change later?

A: My first reaction was "overwhelmed" – even a bit resistant. At first I was particularly against the campus integration. The patients in the East Campus were long‑term cases I had cared for for years; I had a strong emotional bond with them. Suddenly being transferred to the main campus, I worried the patients wouldn't adapt. Also, my home was near the East Campus a 10 minute commute. After integration, I had to travel across the whole city, adding 40 minutes. That was definitely unpleasant. With the EMR upgrade, I felt lost at first. I had used the old system for six years and could operate it with my eyes closed. The new system was far too complex-three different modes for order entry, plus template use, data linkage, etc. After the first training session I was completely confused, afraid I wouldn't learn it and that it would affect my teaching and daily nursing. With the ERAS standardisation, I felt anxious at first-afraid of forgetting the procedures and making mistakes that would harm patients. But later I gradually adapted. After integration, patients were managed together, making consultations and tests more convenient. Although the commute was longer, the hospital added a shuttle bus, so it became acceptable. Once I became proficient with the EMR, querying patient histories and compiling nursing data became very fast, and automatic reminders for expiring orders saved a lot of trouble. After ERAS standardisation, patients recovered more quickly and had fewer complications. Seeing them discharged earlier gave me a sense of accomplishment. My attitude changed from resistance and confusion to acceptance.

Q: You mentioned working overtime continuously during the intensive change period while also teaching. Did you encounter any specific difficulties for example, poor handovers, inadequate training, or poor communication with other departments? Could you give a most memorable example?

A: Too many. The most memorable was when the campus integration and EMR upgrade overlapped. Once, we needed to transfer 15 postoperative patients from the East Campus to the main campus. I was responsible for coordinating. The plan was to leave at 10 am, but the night before, after the EMR upgrade, we couldn't retrieve patients' preoperative test reports or medication records. I went to the hospital at 5 am, manually checked the old paper records, and contacted the IT engineer for emergency help. It took until 11 am to sort out the patient information. After arriving at the main campus, the beds and equipment in the new ward hadn't been fully tested, which delayed patient placement by another hour. That day I worked from 5 am to 3 pm without even drinking water, and then back in the department I still had to explain the new system's operation to the junior nurses. I really felt I couldn't go on.

Q: When changes disrupted your work rhythm and added extra tasks, how did you feel inside?

A: I felt both tired and wronged, with a bit of irritation. Every day after work my whole body ached. I just wanted to collapse on the sofa I didn't even have the energy to cook. My child is only five years old. Previously I told him a story every night, but during that period my husband had to do it. When my child asked, "Mummy, why won't you play with me?" I couldn't help crying. The most devastating time was in January 2023 one month after the campus integration, before we had fully mastered the EMR, the ERAS training started, and I had to teach three new nurses. One day, an ERAS patient under my care had postoperative nausea and vomiting. I adjusted the patient's position and medication according to the new protocol, but when I tried to record it in the system, I was unfamiliar with the template and spent 40 minutes without finishing. The head nurse urged me to submit, the new nurses asked me EMR questions, and just then my husband called to say our child had a fever. I burst into tears at the nurses' station, feeling completely helpless. I even thought, "I can't do this anymore it's too much." Later, the head nurse gave me half a day off to go home and see my child, and my colleagues helped finish the remaining work. Gradually I recovered.

Q: Those feelings are very real. After experiencing so many intensive changes over a long period, did you notice any changes in your work state?

A: Yes, quite obvious. During the six months of most intensive changes, my work motivation visibly declined. Previously I would eagerly volunteer for new tasks. During that period, I dreaded seeing "new notice" or "new training" and would unconsciously procrastinate. For example, when the head nurse asked me to organise ERAS teaching materials, I put it off for three days and did a half‑hearted job. My sense of responsibility also slackened. Once, while recording electronic nursing notes, I was so tired that I wrote "blood pressure at 8 hours post-op" instead of "6 hours post-op". Fortunately, the head nurse caught it during review, so no serious consequences occurred. My work efficiency dropped significantly -a nursing note that used to take 15 minutes now took 30, and I often made mistakes. I had no patience when teaching new nurses. Sometimes when they asked repetitive questions, I would get a bit impatient and my tone was not good. Now I think I owe those girls an apology.My husband said I was like a 'powder keg' during that time, losing my temper over small things.But later, when the changes gradually stabilised and I adjusted my state, these problems improved.

Q: Apart from affecting work, did the fatigue from these changes affect your psychological state and daily life -for example, your mood, sleep, family relationships, social life? Could you be specific?

A: Huge impact. Psychologically, I was very anxious during that period and often suffered from insomnia. My mind was always full of thoughts: "Tomorrow I have to train on the new system", "Which ERAS step haven't I memorised?", "The new nurses' training tasks aren't finished". Sometimes I couldn't sleep until 2 am, and the next day I felt dazed at work. My mood was terrible-I lost my temper easily over small things. Once, my child accidentally knocked over a water cup, and I couldn't help but yell at him. Afterwards, I felt very sorry. In daily life, I used to take my child to the park on weekends and have meals with my parents. During that period, weekends were either overtime, training, or catching up on sleep. I didn't take my child out for several months. Physically, due to long‑term sleep deprivation and stress, I often had dizziness and stomach pain. A hospital check‑up diagnosed neurasthenia and gastritis. I was prescribed medicine but didn't have time to take it properly. Only when the pace of changes slowed down did I gradually recover.

Q: You mentioned that you and your colleagues helped each other. When you felt exhausted, apart from colleague support, what coping methods did you use? Were they effective?

A: At first I just toughed it out, thinking "everyone is busy, I can't hold them back". But the more I toughed it out, the more tired I became. Later I started to actively adjust. Several methods worked. First, complaining with colleagues in the department – we shared our daily frustrations, like "the system crashed again today" or "filling out the ERAS assessment form makes my hand sore". After complaining, I felt much better, and we also shared tips – for example, if someone discovered a shortcut in the EMR, they would tell everyone. Second, self‑relaxation – On my way home from work every day, I listen to soothing music, take a hot bath at home, and sometimes do 10 minutes of meditation to help relieve anxiety. Third, proactively taking time off – when I really couldn't cope, I would ask the head nurse for half a day off to sleep in or play with my child to reset my state. Fourth, asking family for help – I talked openly with my husband about my stress, and afterwards he took on more housework and childcare so I could rest properly.

Q: Based on the changes you experienced, what specific factors do you think make nurses more prone to change fatigue? For example, the pace of change, organisational support, personal resources – could you elaborate?

A: I think there are three main aspects. First, changes come too intensively with no buffer period. The campus integration, EMR upgrade, and ERAS standardisation were less than three months apart. Just as we adapted to one, the next arrived. There was no time to digest – we could only run along passively, and the fatigue naturally grew heavier. Second, organisational support was not precise enough. For example, the EMR training was uniformly delivered as "theory + demonstration". But surgical nurses have many surgeries and overtime; many didn't have the energy to attend fully. Moreover, the training was too general, with no specific guidance for surgical operations – such as how to use the postoperative nursing note template or how to quickly verify surgical orders. These key issues were not explained thoroughly, so we still struggled afterwards. Staffing didn't keep up either. Although the number of beds was reduced after integration, the surgical patient volume didn't drop, and changes added many extra tasks-like ERAS assessments and EMR backfill -but the number of nurses didn't increase. So we had to do the work of two people, doubling the fatigue. Third, personal resources were overdrawn. Time and energy were certainly insufficient-every day we were rushed off our feet, and after work there was training and teaching, with no rest time. In terms of professional skills, changes like the EMR and smart nursing systems were new challenges that required relearning. And I was responsible for teaching-if I didn't master it myself, I couldn't teach others, which added pressure. Although I had support from colleagues and family, sometimes during emergencies like system crashes or sudden changes in a patient's condition – I still felt isolated. Professional identity was also affected. During that period, I often felt that "every day is just coping with changes; I don't have the heart to care for patients properly". I even doubted whether I was suited to be a nurse.

Q: You mentioned that organisational support was not precise enough. Conversely, were there any organisational support measures that you found "particularly useful" in significantly reducing fatigue?

A: Yes, there were useful ones. For example, in the later stage of the EMR upgrade, the hospital stationed IT technicians in our department for a week to solve clinical problems. I reported that "the postoperative nursing note template is too complicated", and the technician customised a simplified template for our surgical department the next day, saving a lot of time. Also, for ERAS training, the head nurse later requested "small‑group, batch training" and invited doctors from anaesthesiology and rehabilitation to come to the department for on-site Q&A -much more effective than the large lectures. In addition, after the campus integration, the hospital provided a shuttle bus and travel subsidies. The head nurse also adjusted schedules reasonably ,for example, allowing us to come in one hour later the day after overtime. These made us feel valued and reduced fatigue. Factors that worsened fatigue included insufficient communication before changes,for the campus integration, we only received one week's notice, so many things couldn't be prepared. Also, when we reported problems,No one responded after we reported problems. We reported the issue of electronic medical record lag three times, and it was only solved after half a month. During that time, we faced crashes and lags every day, which was really heartbreaking. Moreover, the performance pay distribution was initially unclear. After ERAS was introduced, our workload increased, but how the pay would be calculated and whether there would be subsidies were not explained at first. People had no motivation, and fatigue was even worse.

Q: As a teaching team leader who also teaches new nurses, do you think nurses with different seniorities,for example, newly hired nurses versus those with over 10 years of experience,experience change fatigue differently?

A: New nurses mainly have a double overlay of "adaptation pressure + skill pressure". They are still learning basic nursing work, and on top of that they have to catch up with changes like the EMR and ERAS pathway. For example, one new nurse I taught had just learned the old system when it was upgraded-I worry every day about making mistakes. The fatigue mainly comes from 'not knowing how to do it' and 'fear of doing it wrong. For nurses with over 10 years of experience,for example, a colleague with 15 years she was very familiar with the old processes and methods, but slow to accept new things. After the EMR upgrade, she still wasn't proficient after two months and often had to ask junior nurses for help, which made her feel a loss of status. Coupled with the fear of being phased out, her fatigue came mainly from "can't learn it" and "not adapting". For those of us in the 5-10 year range,the backbone we have to adapt to changes ourselves while also teaching new nurses and helping older nurses. We are the "sandwich layer", with the most pressure and the most complex fatigue,both pressure from skill learning and pressure from teaching and work integration.

Q: When you already felt "change fatigue", how did that affect your view of subsequent new changes?

A: When I wasn't fatigued,for example, in my first three years of work–when the hospital introduced a new change, my first reaction was "I can learn something new". I would actively read the notice and ask the head nurse for details. I looked forward to changes. But after experiencing the intensive wave of campus integration, EMR upgrade, and ERAS pathway, I was completely exhausted. When I received a new change notice after that, my first reaction was "why another change? Can't we have a break?" I felt a wave of resistance, even irritation. For example, earlier this year when the "Nursing Quality Sensitive Indicator Real‑time Monitoring System" was introduced, seeing the notice in the department group chat, my first thought was not "how can this system help us optimise things", but "another new system to learn, another new form to fill out,I just got familiar with the EMR and ERAS process, why another one?" I instinctively wanted to procrastinate, not to learn about it. I thought, "put it off as long as possible". I couldn't help doubting: "Is this change again not considering the actual situation?" "Will it just be a fuss that fades away?" I would complain with colleagues: "Anyway, we'll just muddle through when the time comes." This sceptical mindset gave me no motivation to proactively adapt,I just wanted to passively cope. Only if the head nurse pushed for data submission or if not learning it would affect my work would I force myself to learn, and then only the "minimum requirement" -just enough to complete indicator reporting. I wouldn't explore how to use the system to improve efficiency. I thought, "it's enough to get by-no need to spend extra energy on it." I even developed a "chain reaction of resistance". Just seeing the words "new notice" or "new training" gave me a headache. No matter what the change was, I prejudged it as "another burden". Once, the hospital introduced a very simple change – "electronic nursing handover" – which was just a change in recording format. But at first I still resisted, thinking "another habit change". Only after the head nurse demonstrated it and I saw that it actually saved time did I slowly accept it. This conditioned reflex of "resistance as soon as I hear 'change'" was the lingering effect of previous change fatigue. Change fatigue made me shift from "actively accepting and adapting" to "passively resisting and going through the motions". Not only was my mindset averse, but my actions procrastinated, and I was filled with doubt and defensiveness. This effect doesn't disappear quickly; it takes a long stable period and full relief of fatigue to slowly recover my previous attitude.

Q: What aspects do you think should be focused on when assessing nurse change fatigue? Besides the emotional state and work behaviours we've discussed, are there any other easily overlooked but important dimensions?

A: I think at least four aspects should be considered. First, emotional state – such as anxiety, irritability, low mood, helplessness the most intuitive. Second, work performance,for example, decreased motivation, lower efficiency, increased error rates, cutting corners. Third, physical reactions insomnia, dizziness, stomach pain, exhaustion. Many people easily overlook physical fatigue, but it is a very important manifestation of change fatigue. Fourth, changes in attitude towards change itself – for example, shifting from support to resistance, from expectation to numbness, and decreased trust in the hospital's changes. This is also crucial if multiple changes have been poorly handled, when a new change is introduced, nurses will lack confidence or even resist. Another easily overlooked point is the impact on professional identity. When change fatigue is severe, nurses may doubt their professional value or even consider changing careers. That should also be included in the assessment.

Q: How do you usually adapt to the impacts brought by changes?

A: The adaptation process is really "crossing the river by feeling the stones" ,it doesn't happen all at once. It went through several stages, and my methods gradually changed. At first it was "tough it out and passive follow‑through". Later it became "actively find methods + use help". Eventually I found my own rhythm. For example, when I couldn't operate the EMR, I printed out the training materials and kept them at the nurses' station. Whenever I had a moment, I flipped through them. When I encountered something I didn't understand, I wrote it down in a small notebook and asked the IT people when I had several questions. Also very important is "leverage + sticking together". It's too hard on your own. I actively asked colleagues for help. For example, there was a junior nurse in the department who was very sensitive to IT tools. When the EMR first launched, she quickly mastered the shortcuts, so I often asked her. This kind of "sticking together" not only solved practical problems but also relieved psychological pressure. Knowing that "I'm not the only one carrying this burden" made my mindset more steady. At the same time, I also made sure to regulate my state – no matter how busy, I took half a day on weekends to be with my family, not letting work completely take over my life. That helped me maintain energy and avoid falling back into fatigue.

Q: You've talked a lot about the implementation of changes. What is your view on the "stable period" between changes?

A: The stable period is extremely important – it's literally a "lifesaver". If there is no stable period after a change and we just keep going non‑stop, even the toughest person will break down. I think at least 3‑6 months of stable period is needed. For example, after a change is implemented, give us three months to become proficient, straighten out the processes, and solve problems. Once everyone is fully adapted and the work rhythm is stable, then introduce the next change. That would greatly reduce fatigue. For example, after the EMR upgrade, it took us four months to become fully proficient. If another change had been introduced during that time, we definitely couldn't have handled it. But in reality, many changes are less than a month apart, with no stable period at all. We just have to grit our teeth and go through them. I really hope that in the future the hospital will introduce changes "step by step" and give us enough time to digest, rather than "rushing progress" and "piling up tasks".

Q: Apart from what we've discussed, is there anything else you would like to add? For example, other feelings related to change fatigue, or suggestions for the hospital's change implementation, or for my research?

A: One more thing,I hope that in the future, when the hospital introduces changes, they will listen more to the opinions of frontline nurses. For example, when developing new processes or new systems, invite us to participate in discussions to see which designs are suitable for clinical practice. That would reduce a lot of subsequent problems and make us more willing to cooperate. For example, if the EMR had been designed with our input from the beginning, there wouldn't have been so many features that didn't meet surgical nursing needs.

Q: Thank you so much. Thank you for your cooperation. Then we can end the interview here,please take a rest.

A: Thank you. I wish your project goes smoothly!
