# Supplementary material for: Nurses’ experiences of change fatigue during healthcare reforms: a qualitative study of psychological distress, impacts, and adaptation
Source: Front Psychiatry. 2026 Jul 22;17:1878913. doi: 10.3389/fpsyt.2026.1878913 (PMC13437614; doi:10.3389/fpsyt.2026.1878913)
Supplement: Supplementary file 5 [file Table12.docx]

Interview Transcript (N12)

Q: Please describe the types of changes you have experienced in your clinical work. Tell me about one or two events that made you feel most stressed and challenged.

A: In clinical work, it might be changes in job position, changes in the environment, or changes in colleagues.

Q: Could you elaborate on that?

A: Personally, after changing my work environment, I still need a long time to adapt. Actually, having clinical work experience already shortens my adaptation time, but I still need some time to adjust to a new position. Moving from one department to another requires re-adaptation. When you go to a new department and deal with new colleagues and new leaders, everyone has different personalities and work styles – that also requires adaptation. In this process, there can be both good and bad influences. The good side is, for example, if I move from a large hospital to a smaller one, I find it relatively easier to adapt. In a large hospital, the rules and regulations are more complete and requirements are stricter. When you go to a smaller hospital, requirements are not as strict in various aspects. The way leaders interact may be more like peers, which makes me feel somewhat relaxed. The stressful factor is the change of environment – mainly unfamiliarity with the environment and unfamiliar colleagues, which brings some pressure.

Q: Which department were you in before, if you don't mind?

A: Sure. I was in neurosurgery, and later I went to rehabilitation medicine.

Q: Had you been exposed to rehabilitation before?

A: Actually, in neurosurgery the focus is more on perioperative nursing. In rehabilitation, it is more about the gradual recovery process. So the characteristics of the two departments are different. Anyone who has worked in neurosurgery knows that it is quite difficult. Going to rehabilitation was a bit better, because rehabilitation has many stable patients, unlike neurosurgery where emergencies and complex conditions are frequent. However, rehabilitation has its own difficulties. Rehabilitation treatment is a long‑term process that requires sufficient patience and professional knowledge from healthcare staff. We need to develop individualised rehabilitation plans for each patient and continuously monitor and adjust them. Moreover, most rehabilitation patients have some degree of physical impairment and may also have negative emotions psychologically, so we need to focus not only on their physical recovery but also on psychological counselling. In neurosurgery, the main effort was on surgical cooperation and management of acute postoperative complications. In rehabilitation, the focus of work changed significantly, and I had to relearn many rehabilitation treatment skills and knowledge-such as various rehabilitation training methods, physical therapy techniques, etc. At the same time, collaboration with multidisciplinary teams-rehabilitation therapists, nutritionists, etc. became more frequent and important. We work together for the patient's recovery. During the process of adapting to this new department, although there was pressure, it also allowed me to learn many new things and broaden my clinical perspective.

Q: Did you choose the department yourself, or was it arranged by the hospital?

A: Arranged by the hospital. My first hospital was where I did my internship-they would ask for nurses' preferences. The second one did not; they made the arrangement directly. Actually, when I first learned that I was assigned to rehabilitation, I felt a bit nervous. After all, moving suddenly from a familiar neurosurgery department to a department I knew little about made me feel uncertain. I also didn't know how rehabilitation would affect my career development. But later, as I gradually adapted, I found it was actually a good opportunity.

Q: Why did you actively change your work environment?

A: For family reasons. My family had to move to a new place, so to facilitate taking care of my family, I changed my workplace, and thus came to a new hospital and a new department.

Q: When you made the decision to change jobs and came to the new hospital for the first time, what were your first feelings?

A: It was quite complex. Adapting to the living and working environment was not that stressful. But adapting to additional factors at the same time,such as a decrease in income,was a very real pressure. At the new hospital, due to differences in hospital size, grade, and compensation system, my income was lower than before. Moreover, in the new hospital, everything had to be restarted building relationships and work achievements from scratch, and career development became more uncertain. Although I faced many pressures at first–including lower income–over time I gradually adapted and discovered the positive aspects of the new work environment. So the emotions were quite mixed. On one hand, I felt the work was easier; on the other hand, my income decreased. Because I am married and have a family to care for, I care a lot about financial issues.

Q: In the process of coping with these changes, how do you assess your ability to handle them?

A: Actually, I am quite confident in myself. I think it is just a matter of time. I know that after three months things will gradually improve. I believe in my ability to adapt to new environments. As I said before about changing departments or hospitals – although it is difficult at first, with the clinical experience and learning ability I have accumulated over the years, I know I will eventually overcome it. I think I have strong psychological resilience when coping with changes. I don't easily give up or become overly anxious because of temporary difficulties and pressure. Moreover, every change is an opportunity for growth, allowing me to learn new knowledge and skills and expand my professional field. I regard each change as a chance to improve myself, constantly challenge myself, and believe that through my own efforts, I can achieve good results in the new job.

Q: With your rich work experience, you can learn and adapt well in any environment. After becoming familiar with the new environment, how did your attitude change?

A: I think coping with change is just a matter of time. With enough time, you become familiar, and after becoming familiar, things flatten out,you both adapt to the work and gradually accept the current environment and compensation.

Q: Compared with your previous work rhythm, is the new work environment slower?

A: Yes. In rehabilitation, the workload is relatively less, and there are fewer procedures compared with neurosurgery.

Q: In the face of change, what factors do you think are more likely to make you feel fatigued?

A: I think the frequency of changes and the communication during changes affect me the most. If rules change frequently and change happens very often, you feel you cannot keep up with the leader's thinking and pace, and what you do may not match what the higher‑ups want. Also, inadequate communication has the same effect – everyone works hard, but why are the results not good?

For example, during a change process, if the goals, requirements, and plans of the change are not communicated clearly and promptly, people have to cross the river by feeling the stones, and work becomes very confusing.Previously, the hospital implemented a new nursing process change. The leader simply said there would be a change, but did not explain specifically how or to what extent,When we nurses implemented it, we had to explore on our own. As a result, everyone did things differently, leading to low work efficiency and easy errors. Moreover, due to poor communication, we didn't know if we were doing it right, which made us anxious. Over time, fatigue set in.The frequency of changes is too high. Before one change is fully adapted, new changes follow one after another. It feels like constantly chasing a goal that can never be reached. The spirit is always in a state of high tension, and both body and mind become very tired.So communication and frequency have a big impact on my fatigue.

Q: These two factors are very important to you. When you already feel change fatigue, how does that affect your view of subsequent new changes?

A: It definitely has an impact. First, when I receive an instruction from a leader, my immediate reaction may be doubt,doubting whether I can do it well, and also doubting whether the leader's decision is correct. After experiencing previous change fatigue, I worry that this change will be like the previous ones–without clear planning and communication, causing everyone to do a lot of useless work. Second, I feel resistance. Because previous changes exhausted me physically and mentally, I instinctively resist new changes and am unwilling to invest energy and time to adapt to new changes. Third, I lack motivation. Even if I reluctantly implement the new change instructions, I will not have the same enthusiasm and drive as before. Instead, I will have a mindset of just completing tasks, rather than actively exploring and innovating.

Q: If the hospital still forcibly implements a change, how does that affect the quality of your coping measures?

A: Personally, my character is such that once I receive an order, I try to do it well within my ability. Even if I am dissatisfied with the change, I still have some standards for myself. Even though sometimes I feel I cannot cope, and there may be a sudden moment of collapse where I think "forget it, just submit these tasks", after careful thought I still try to do it to a level that I think is presentable.

Q: So regardless of whether the hospital's change is good or bad, out of self‑requirement, you won't be particularly perfunctory towards the change event.

A: Yes, I won't be particularly negative.

Q: In the process of coping with changes, how does it affect your psychological state?

A: As I just said, even though I still do it, I am definitely not as enthusiastic or creative. Overall, the quality of completion is definitely not as high. It also generates negative emotions, such as anxiety and frustration. Anxiety is the most common feeling, after all, every change is full of uncertainty. I don't know what the result of this change will be, or whether it will exhaust me again for some reason like before. Because I don't know what the result of this change will be, or whether it will exhaust me again due to poor communication or excessive frequency. Moreover, during the adaptation process, various difficulties and problems may arise. When I cannot solve them at once, I feel frustrated. Because I have doubts and resistance towards the new change, when carrying out change tasks,although my professional ethics and personal standards won't let me do a terrible job,I still feel somewhat distracted and worry about doing poorly, which aggravates the anxiety. Being in such negative emotions for a long time increases psychological pressure and may even affect daily life and family relationships.

Q: How do you relieve the negative emotions that arise when coping with changes?

A: I try to detach from the environment-persuade myself not to think about these things any more, or go out into nature, completely relax my mindset, or stay at home and binge‑watch dramas to distract myself.

Q: How do the impacts of changes affect your family?

A: There is definitely some impact on the family. For example, some changes require overtime, which reduces time with family. Also, family members' routines may be disrupted. For instance, if I work late because of newly added tasks, my family may delay dinner waiting for me, affecting everyone's biological clock. In addition, if I can't control my negative emotions from work changes and bring them home, it also affects the family atmosphere. I might lose my temper with my family over small things, making the relationships among family members a bit tense. Also, if work changes cause fluctuations in my income, that affects the family's financial planning.

Q: How supportive is your family of your work?

A: My family is very supportive of my work. My mother always says that girls should have their own careers. My husband respects me very much. He knows I have a lot of pressure at work, so he keeps the household affairs in order, leaving me with no worries. When I am upset, he listens patiently, gives me comfort and advice, and helps relieve my stress.

Q: What is your view on the stable period during changes?

A: Personally, I think the existence of a stable period is very good. It allows everyone to gradually accept and adapt to new things. Most importantly, it allows people to understand the change, then slowly accept it, and gives themselves time to adapt. It is like a stabiliser very necessary.

Q: In your work, has there ever been a moment when you felt you simply couldn't cope with the changes?

A: Definitely. For example, when there are so many tasks that I feel I cannot handle them, I will certainly collapse for a moment, but eventually I have to compose myself and continue.

Q: Could you elaborate?

A: For example, being given a task that must be completed in a very short time at such times I feel a lot of pressure, very anxious, and I doubt myself: am I incompetent or what? But I have to do it, and inside I am extremely tense and tormented. Once, the hospital introduced a new information system change, requiring nurses to become proficient and apply it in daily nursing work within one month. During that period, in addition to my regular nursing work, I had to spend a lot of time learning the new system and ensuring no mistakes during operation. Moreover, in the early stage of the new system, various problems occurred unstable data transmission, system lags which greatly reduced our work efficiency and extended working hours. Adding other things family trivial matters, friction between colleagues – all this pressure piled up. And nursing itself is already a high‑pressure profession with a very saturated workload. Everyone has an emotional bottom line. Whether it is hospital changes or other things, if everything piles up at once, I feel like I cannot breathe.

Q: In your previous work, did the hospital provide any corresponding support when implementing changes – for example, training courses or psychological counselling targeting nurses' mental state or abilities?

A: Generally, yes. Nowadays hospitals emphasise various forms of humanistic care for both employees and patients,so they definitely do something. However, emotional support may be weaker, but it exists.

Q: In what forms?

A: Both online and offline. Offline, for example, giving comfort gifts during holidays, and sometimes leaders have regular heart‑to‑heart talks, asking about recent problems and trying to help solve them. Online, there are knowledge popularisation sessions. I think offline is more effective.

Q: Have you participated in psychological counselling or training?

A: Training exists. In terms of effectiveness, if you are willing to listen, you definitely gain something. It also depends greatly on personal factors. If a person actively absorbs the training content, they can get a lot of help from it – relieving psychological pressure and improving their ability to cope with change. But if someone attends with a perfunctory attitude, it may not have much effect. So the effectiveness of training is closely related to the individual's participation and learning attitude. The hospital is also continuously improving, hoping to provide more targeted and effective training and psychological counselling to help us better cope with changes at work.

Q: Regarding the timing of activities – does the hospital just notify everyone, or do they ask for opinions to set a time?

A: Generally, the hospital considers it and takes everyone's time into account. But it is probably unrealistic to ask everyone's opinion.

Q: When you attended those training sessions or lectures, was it during your rest time or during working hours?

A: Mostly during rest time. If you attend, it counts as overtime,counts as work hours, and there may be opportunities to arrange time off.

Q: Based on your experience, when assessing the concept of nurse change fatigue, what aspects do you suggest researchers should focus on more – for example, nurses' emotional state, work behaviours, coping styles, attitudes towards change, etc.?

A: I think we should pay more attention to nurses' emotional fluctuations. Nowadays there is a constant emphasis on caring for patients, but I think more attention should also be paid to nurses' emotions. Because clinical work is busy and stressful, I hope nurses' mental state can be given more attention. Also, coping styles towards change – if nurses are unwilling to face change, it may lead to failure of the change, since nurses are often the primary participants in many changes. Moreover, the nurse population is relatively large compared with other healthcare groups. If nurses do not adopt appropriate coping styles, implementing changes will be very difficult. Attitude towards change is also crucial – whether nurses actively embrace or passively resist change directly affects the speed and final outcome of change implementation. So when assessing nurse change fatigue, many aspects need to be considered together to more comprehensively and accurately understand nurses' state and needs during the change process.

Q: Is there anything else you would like to add regarding nurse change fatigue?

A: Nothing else.

Q: Thank you for your cooperation.
