# Supplementary material for: Nurses’ experiences of change fatigue during healthcare reforms: a qualitative study of psychological distress, impacts, and adaptation
Source: Front Psychiatry. 2026 Jul 22;17:1878913. doi: 10.3389/fpsyt.2026.1878913 (PMC13437614; doi:10.3389/fpsyt.2026.1878913)
Supplement: Supplementary file 6 [file Table13.docx]

Interview Transcript (N13)

Q: Please describe the types of changes you have experienced in your clinical work. Tell me about one or two events that made you feel most stressed and challenged, or that had a big impact on you and in which you were highly involved.

A: (Ponders for a moment) If we talk about the highest frequency and most direct impact, it should be the "Smart Operating Room Management Platform" that started at the end of 2021 and is still being continuously improved. It integrated several independent systems instrument management, staff scheduling, and patient information.

Q: You mentioned the "Smart Operating Room Management Platform". Could you describe the main differences between it and the old system? Which aspects of your daily work did it change?

A: Huge differences. Before, we were in a "semi-manual" state: surgical schedules were on paper shift lists plus department group notifications; instrument counts were done by two people reading a list aloud and recording manually; patient information had to be checked on a separate computer system. The new platform claims "one‑screen management": the screen at the operating room entrance displays all information in real time; instruments are counted with a scanner; every operation must be confirmed on a tablet.

Q: When the platform was first launched, how did the department notify and arrange things? Do you remember the situation when you first used it?

A: (Recalling) The notice was very sudden. Monday morning meeting, the head nurse said the hospital required a full trial run the following week. Two training sessions were given, each one hour, delivered by someone from the software company – very general. The first time I used it for real was during an emergency appendectomy. The instrument nurse and I had to assist the surgeon while also keeping an eye on the tablet. The scanner sometimes wouldn't read the barcodes, and when we got anxious it became even more chaotic. Instrument counting took nearly 15 minutes longer than usual. The lead surgeon didn't say anything, but the waiting atmosphere... (shakes head) very stressful.

Q: You used the phrase "very stressful". Back then, what was your first reaction?

A: For the first month or two, it felt like a threat. Because it directly threatened the two most core things in our operating room: safety and efficiency. A mistake in counting is a big deal, and disrupting the surgical rhythm affects the whole team. We complained a lot privately, feeling that the leadership was just chasing a smart‑hospital image and creating extra trouble for us frontline staff.

Q: You used the strong expression "threatened safety". Did any specific event happen that made you feel strongly that "safety was threatened"?

A: Yes. During an orthopedic surgery, a set of non-routine instruments was marked as “Issued” in the system, but we couldn’t find any record of them being “Used” on our operating room computer, no matter how hard we looked. According to protocol, we were required to suspend the surgery. However, the day’s surgeries had already been scheduled, and postponing even one procedure would have disrupted the entire schedule. As a large Grade A tertiary hospital with a heavy surgical workload, our operating rooms are typically fully booked throughout the day, leaving no gaps in the schedule. Therefore, we had no choice but to set this issue aside and proceed with the surgery. After work that evening, we turned the entire operating room upside down and finally discovered that the barcode label on the instrument pack had been affixed incorrectly. Although it turned out to be a false alarm, those of us on duty spent the entire day worrying about it, and it forced us to work several extra hours—an unnecessary increase in our workload. Fortunately, the item wasn’t actually lost. If it had been, regulations stipulate that the cost of the lost instrument would be deducted from our performance bonuses. Living in a first-tier city, I’m very sensitive to financial issues—after all, isn’t all this exhaustion every day just for the money? If my performance bonus were deducted because of this, I’d be really upset.After this incident, everyone trusted the new system even less. I do understand the hospital's original intention–our hospital is quite famous in Shanghai, and information technology is the only way forward. The hospital needs to develop, society moves forward–change is inevitable. What I resist is not the change itself, but the process. It feels like the decision‑makers are too focused on speed, have underestimated the operating habits of frontline staff and the potential problems, and have given too little preparation and buffer time. It's like... (searching for a metaphor) ordering soldiers to switch to a new rifle immediately, without enough live‑fire training, and sending them straight into battle.

Q: In the process of coping with these changes, what personal resources of yours were more likely to cause you physical and mental fatigue?

A: First, I feel that my time is heavily occupied, especially non‑working time for learning. When the system was first launched, I often stayed in the department after work to get familiar with it, afraid of making a fool of myself on the operating table the next day–the chief surgeon has a very bad temper and will scold you directly if you are not competent. Second, my sense of professional identity and meaning. I chose nursing, especially operating room nursing, because I love the sense of value that comes from precision, collaboration, and creating life-saving miracles at critical moments. But now, I feel that a large amount of my energy is consumed in adapting to processes, filling in the system, and coping with inspections. I start to doubt: how much of this effort is truly contributing to patient care itself? When ensuring correct system entry becomes almost as important as ensuring patient safety -and is even more intuitive and easier to assess-the sacredness of the work is diluted. This split in professional identity is the deepest and most dangerous kind of fatigue. Finally, I feel that my emotions are also drained. Anxiety has become the norm. It's not fear of hardship or tiredness-it's fear of making mistakes. Not nursing safety mistakes, but mistakes of not following the new process. Even if my traditional method is more efficient and safer, as long as it doesn't comply with the rules, it may be flagged for quality control. After work, my mind is still thinking: did I forget to scan some step today? Will my report have problems? This kind of continuous, low‑intensity anxiety is, I think, harder to recover from than the tension of a major resuscitation. Tension is short‑lived, but this anxiety is pervasive and persistent. I have worked in the operating room for so long-I should be skilled and confident. But in front of the new system, I feel like a novice. This gap in self‑worth is very tormenting.

Q: Do you bring this anxiety and frustration home? How does it affect your family life – for example, your relationship with your girlfriend and children?

A: (Expression slightly heavy) Yes. During that period, I spoke very little after coming home and was very tired. My girlfriend is a paediatric nurse; she understands, but she also has complaints. (Pause) I think what consumes me most is not physical energy, but this sense of indebtedness to my family. This is a deeper emotional resource overdraft, making me feel that I have not fulfilled my responsibilities as a husband and father.

Q: In your work environment, what kind of support do you think helps you cope with these consumptions?

A: I think colleague support is the most critical. Our operating room team has a very good atmosphere. Everyone helps each other. The atmosphere of a department is extremely important. If the atmosphere is bad, and then you encounter a difficult change, the whole person feels very tired. Second, leadership. The leader is the core of a team. How the change is done, how it is implemented, why it is done – all of these depend on the leader. The leader's ability is also very important for whether a change succeeds. A good leader can give us direction during the change, understand the difficulties of frontline staff, coordinate relationships, and make the change progress more smoothly.

Q: In the process of coping with changes, how do you deal with the negative emotions that arise?

A: First, since I know change is inevitable, I shift my mindset – I "brainwash myself". I keep telling myself that information technology is the trend; resisting is worse than mastering it early. The earlier you learn, the easier it becomes. Once my mindset changes, acceptance is less painful. Second, I also appropriately slack off. I prioritise tasks, ensuring that patient safety and core operations are flawless. For non‑critical things, I choose not to do them urgently and give myself proper rest time. Also, I complain with colleagues – that feels very useful, because emotions need to be vented. When I have time, I exercise. After all, as a male, my hobbies are basically gaming, working out, drinking, etc. Exercise is good for the body, and sweating helps me temporarily forget work worries, which is also good for emotional release.

Q: When you hear a new change directive, what psychological process do you go through?

A: First, my heart tightens – a sudden sinking feeling. Then I quickly estimate how much time it will take and whether it will cause new problems. Finally, I accept it reluctantly. In recent years, I feel my ability has declined a bit... (wry smile) To be honest, I feel my adaptability is declining. When I first joined eight years ago, I learned everything quickly and was full of energy. Although I have more experience now, I always feel like my phone battery is aging, and the charge runs out quickly. I still have confidence in my learning ability, but I am a bit afraid of the frequent, intensive pace of change. It feels like being pushed along, with no chance to catch my breath.

Q: When you already feel "change fatigue", how does that affect your view of subsequent new changes?

A: How to put it... To be honest, At my age, I have a family and children. I just want to live a stable life. Because there are many things in life, I don't have strong enthusiasm for changes now. Even if a change might really be a good thing, I can't muster any enthusiasm to believe in it or look forward to it. Now I just do my job well, get my salary. Isn't it just earning a living? I don't think too much about anything else.

Q: Has this state affected your work behaviour, work quality, or even your professional identity?

A: In terms of ensuring treatment safety without errors, I still choose to operate in areas I am familiar with. Even if the old way is slower but safer, while the new way may be faster but has uncertainties, I tend to use the old way. In terms of professional identity... (pause, thinking) I still love the nursing profession, especially operating room nursing. The sense of achievement from successfully assisting in a complex surgery is irreplaceable. However, my sense of identification and loyalty towards the hospital as an organisation has indeed been worn down. Sometimes I suspect that when leaders make decisions, they may not start from reality at all, so some decisions are very detached from the thinking and needs of the grassroots. I think the nursing profession is sacred, professional, and warm, but from the leadership's perspective, it may just be miscellaneous work. For example, when salary reduction reforms happen, nurses are the first to be targeted. But nurses do the hardest and dirtiest work. This invisible sense of hierarchy has greatly weakened my professional identity as a nurse. Now I just do my job well, get my salary–isn't it just earning a living? I don't think too much about anything else.

Q: How has this state affected your career planning?

A: To be honest, about half a year ago, I considered job‑hopping. There was a high-end private hospital with an operating room position, offering 30% higher salary and less workload. I almost went there-after all, I work for money. The hospital has various reforms, but my performance pay hasn't increased. However, I thought it over carefully and ultimately didn't go. Because I have a strong sense of belonging to my current department-the military hospital background gives a certain expectation of stability,and my family values the stability of my job. I think a public hospital is more reliable. Also, I did my internship at this hospital, have worked here for 8 years, plus the internship nearly 1 year-that's almost 10 years. There is some emotional attachment. But the thought did arise.

Q: What is your view on the stable period during changes?

A: To be honest, being able to stabilise for a while is certainly good. At least I don't have to worry every day about the process changing again, the system updating again. During that time, the work at hand can finally become muscle memory, and cooperation with colleagues can regain some of the old tacit understanding. Overtime might also decrease a bit. It's like giving an always‑high‑speed machine a slight cooling‑down. But the problem is, my feeling has changed now. Before, the stable period was a time for accumulation and growth – I could think about how to do my work better. Now, for me personally, the stable period is more like a time for psychological and emotional recovery. If there really were a stable period, I would use it to quickly repair the family relationships that were damaged before, to sleep back some of the overdrawn energy, or even just to do nothing at all and let my mind go blank. I am not preparing for the next leap forward ,I am building up some resistance for the possible turmoil to come.

Q: Based on your experience, what types of nurses are more prone to this kind of fatigue? Who is relatively more resilient?

A: I think there are two types of people who are prone to fatigue. One is nurses with a very strong sense of responsibility and perfectionism,because the initial stage of change is inevitably accompanied by chaos and errors, and they suffer terribly. The other is colleagues with heavy family burdens and little external support,they have too few personal resources to allocate. Both of these types are already under considerable pressure even without change. When a change event arrives, it is an overlay of new pressure, so they are more likely to feel change fatigue. Relatively resilient people are also of two types. One is technically oriented nurses who are very flexible in mindset and like to tinker with new things,they treat change like playing a game, levelling up, and are naturally curious about new things. The other is nurses who have strong hobbies or family emotional support,work is not their only source of energy and value. Their emotions have a huge outlet, so they generate fewer negative emotions.

Q: I understand your pressure. Is there anything else you would like to add about change fatigue?

A: I think the pace and density of change are very important. Actually, we are not afraid of change,we are afraid of non‑stop, overlapping change. Before one change is digested, another is piled on. That gives people no chance to breathe or feel a sense of achievement. Also, the evaluation criteria for change need to be revised. We should not only look at data reporting rates and system usage rates, but also assess the potential impact of change on frontline workers' mental health, job satisfaction, team cohesion, etc. Some changes sacrifice people's sustainability for short‑term improvements in management metrics – that is not worth it.

Q: Thank you for your answers. The interview is now concluded.
