# Supplementary material for: Nurses’ experiences of change fatigue during healthcare reforms: a qualitative study of psychological distress, impacts, and adaptation
Source: Front Psychiatry. 2026 Jul 22;17:1878913. doi: 10.3389/fpsyt.2026.1878913 (PMC13437614; doi:10.3389/fpsyt.2026.1878913)
Supplement: Supplementary file 7 [file Table14.docx]

Interview Transcript (N14)

Q: Please describe the change events you have experienced at work. What changes make you feel stressed or challenged?

A: I think administrative changes stress me out more. Clinical work changes are actually acceptable.

Q: Could you elaborate on that?

A: For example, the work style of a new leader and their new requirements–I find that more stressful.

Q: Can you give a specific example of something that made you feel stressed?

A: Actually, our department is fine. To give an example, it might be a bit inconvenient to say.

Q: OK, I understand. Are you still in neurosurgery?

A: Yes, still in neurosurgery.

Q: During previous hospital changes, what were your thoughts and feelings?

A: I just thought I would try my best. If I can do something well, I will do my best.

Q: So your attitude towards change is to do what you can.

A: Yes, that's right,also a bit of wait and see.

Q: From what you hear online or from people around you, it seems the biggest change recently is performance pay reform, right?

A: Yes.

Q: Could you share your views on that?

A: The biggest impact of performance pay reform on us was probably in 2019 and 2020. Both medical and nursing underwent major reforms, but there was a strong bias towards the medical side,nursing was affected more than medicine.

Q: Do you mean that some of nurses' benefits were shifted to doctors, making nurses' work benefits less favourable?

A: Yes, that's right.

Q: How long did this pattern last?

A: After 2019 and 2020, the reform has been like that ever since.

Q: When changes in your work experience increase difficulty or require adjustments, what is your first thought?

A: At first, I definitely feel some resistance and anxiety. After all, the work model and rules change, and you have to re‑adapt to new requirements and pace. For example, with performance pay reform, when nurses' benefits are affected, you feel unbalanced. But after calming down, I tell myself I have to face it,just try to learn, because I have no choice. I can't decide whether to reform or not, so I stop struggling and force myself to accept it. Actually, the main problem is being unfamiliar. Once you become familiar, it feels better and you can solve some problems.

Q: As the reform progresses and you become more familiar, how does your inner feeling or your view of the reform change?

A: As the reform advances, I gradually adapt. In this process, we nurses also grow and improve. After adapting to the new work model, work efficiency improves. Still, I hope that in future reforms the hospital will consider the nursing group more after all, nursing is an indispensable part of the hospital. Now, I am not as resistant to reform as I was at the beginning. I hope it can keep improving, so the hospital develops better and our work becomes more comfortable.

Q: What are your thoughts on the nursing work environment, model, or work content?

A: I think we spend too much time on nursing documentation and various assessment scales.

Q: So other tasks increase nursing workload and burden. In the process of coping with these changes, what personal resources are more likely to cause you physical and mental fatigue?

A: I think interpersonal relationships and professional identity affect me the most.

Q: Could you elaborate?

A: I feel that if you work with someone you get along with comfortably, and your relationship with colleagues is harmonious, your mood at work is different. Also, recognition from patients or colleagues is very pleasing–it makes me happy. For me, happiness is quite important.

Q: So these two things directly affect your mood, and when you are in a bad mood, you are more prone to fatigue.

A: Yes.

Q: If you already feel change fatigue, how does that affect your view of subsequent new changes?

A: If I already feel change fatigue, I would be more cautious and worried about subsequent new changes. After all, having gone through so many changes, I am already somewhat exhausted physically and mentally. I would worry that new changes would bring new work model adjustments, benefit changes, etc. I might take a more conservative attitude at first, not as active in trying and adapting as before. I would pay more attention to the impact of new changes on my work and life. If it feels like it might add more pressure and burden, I would be more resistant. Still, I would try to be rational. If the new change truly has a positive effect on the hospital and department, I would still try to cooperate, but with more concerns and unease, not as optimistic and fearless as when I first faced changes.

Q: When you are stressed at work, how do you relieve it?

A: Generally, I go hiking, bask in the sun, or get a massage.

Q: How effective are these methods for you?

A: I think they work quite well–I can forget work worries and relax physically and mentally.

Q: Does the hospital provide any support to relieve nurses' physical and mental health?

A: Yes.

Q: Have you participated?

A: Yes, but rarely. Mainly because I am too busy with work to find time.

Q: How did you find it?

A: Because team‑building activities are with hospital people, I don't feel as relaxed. The forms of team-building are relatively fixed, so it's hard to truly unwind. Sometimes people unconsciously talk about work during the activity, which brings the pressure back. I hope the hospital could give us more autonomous adjustment time and space in daily work, so we can do some relaxing activities we enjoy during breaks, rather than always being in a high‑tension work state.

Q: In the process of coping with changes, what emotions do you experience?

A: The emotions are quite complex. At first, as mentioned, resistance and anxiety. After all, change brings a lot of uncertainty. Suddenly changing a familiar work pattern makes anyone feel unsure. Sometimes I worry that I won't be able to adapt to new requirements. If I don't do my work well, it will not only affect my performance but also cause psychological burden. As the reform progresses, during the effort to adapt, I feel a sense of confusion. For example, after the performance pay reform, nurses' benefits and work models changed – I don't know where my career direction is going; I feel a bit lost.

Q: Facing these emotions, how do you assess your ability to cope?

A: I think that since change comes from the top down, the leaders will analyse it first. If we are under a lot of pressure, the leaders will analyse it. If it's really not working, we will give feedback to the leaders to see how to solve or better optimise things.

Q: Do the leaders listen to your feedback?

A: Our head nurse does take our opinions. If everyone feels uncomfortable with a change, our leader gives us positive feedback.

Q: What is your view on the stable period during changes?

A: I think if there is a stable period, does that mean the reform can be sustained? If the reform can stay in a stable period, then we nurses can have more stable work and feel more at ease.I don't have to worry about sudden new changes that would require us to adapt again. A stable period also allows us more time and energy to improve our professional skills and better serve patients. Moreover, in a stable environment, colleagues' cooperation will be more tacit, and work efficiency will be higher. I also hope that during the stable period, the hospital will pay more attention to nurses' needs, further optimise some problems left over from previous reforms, so that the reform truly benefits every nurse – then we will have more motivation to work.

Q: For you personally, what is the impact of having a stable period?

A: I think if everyone can accept it, it seems reasonable.

Q: From your perspective, could you give us researchers some suggestions–for example, when assessing nurse change fatigue, what aspects should we pay more attention to?

A: I think we should pay attention to workload saturation. If the reform increases workload, nurses' physical and mental burden will certainly increase, making change fatigue more likely. We could look at nurses' daily working hours, number of patients cared for, time spent on non-direct nursing tasks like documentation and assessment scales. Also, changes in salary and benefits are key. As mentioned earlier with the performance pay reform, nurses' benefits were affected, which greatly dampens work motivation and easily triggers change fatigue. So we should examine changes in nurses' income, bonuses, subsidies, etc. Another aspect – interpersonal relationships should not be underestimated. The cooperation atmosphere among colleagues, relationships with patients and families, all affect nurses' work mood. A harmonious work environment relieves pressure, while tense relationships make nurses more prone to fatigue.

Q: Have past hospital changes caused you negative emotions?

A: I take it easy now,if I can't change the environment, I change my mindset. I understand that the hospital is trying to move in a positive direction. If a change is positive or moving in a good direction, and there is an adaptation period for everyone, then after that period, if everyone's development is moving upward, I think the reform is useful.

Q: OK. Before implementing a change, does the hospital provide training to relevant staff?

A: Yes, someone provides training. For example, if we need to learn something new or a new system, someone in our department learns it first and then explains or trains everyone.

Q: What situations do you think would further aggravate nurses' change fatigue?

A: I think if a change is implemented hastily without sufficient preparation and transition time, nurses have to adapt to new work models and requirements immediately–that makes everyone feel huge pressure, and change fatigue becomes worse. Also, if changes occur frequently without enough stable periods, nurses are constantly adapting to new changes, never able to form a stable work rhythm and pattern–that also aggravates change fatigue. Additionally, if a reform is carried out without clearly explaining its purpose and expected effects, nurses just blindly follow the requirements. Over time, they feel the work is meaningless, become bored with change, or communication is not smooth, or when problems arise, no one solves them ,I think these are all problems.

Q: Have you experienced such situations?

A: Yes. The previous system upgrade was like that very rushed, the new system went online with many bugs and unfixed issues. Nurses frequently encountered problems and didn't know how to solve them. Without enough time to familiarise ourselves, we had to cope hastily during work, greatly reducing efficiency and increasing error rates. Moreover, smooth work coordination with colleagues became bumpy, and relationships became tense. The hospital did not explain the purpose or expected outcomes of the upgrade, so everyone had to grope their way through, full of confusion. Many nurses complained privately, some even felt it was just causing trouble. Change fatigue increased sharply. Later, after a period of debugging and fixing, the system gradually stabilised, but it left a shadow in everyone's hearts, making us more worried about future changes. Also, sometimes with system maintenance or material updates, if problems arise, clinical nurses find it hard to find the right person to handle them,but now it's okay.

Q: How did that affect your mood at the time?

A: My mood was particularly bad,already under a lot of pressure. Every day at work felt like a battlefield. The hardware couldn't keep up, causing low efficiency.I have a relatively impatient personality. If I feel someone is holding me back, I get very agitated. Colleague relationships also become tense. The workload in the department is already very saturated, everyone is holding back anger at work, and now new changes make everyone unfamiliar, so the work atmosphere is particularly tense. I also felt confused. As frontline staff, we don't really know what all this turmoil is for. I felt trapped in anxiety and irritation every day, my enthusiasm for work hit rock bottom, and sometimes I even doubted whether I was suited for this job.

Q: What is your mindset when facing change?

A: I'm actually okay. As long as the head nurse doesn't cause me trouble and families aren't too naggy, I think nursing work is okay for me – I believe I am competent.

Q: Do you like nursing work?

A: I wouldn't say I like it, but I am confident in my ability. I have worked for many years, and I feel that if I want to do something, I can do it well. I have a relatively strong personality,I think if others can do it, I should be able to do it too.

Q: Is there anything else you would like to add about this topic?

A: Not for now.

Q: Thank you for your cooperation.
