# Supplementary material for: Nurses’ experiences of change fatigue during healthcare reforms: a qualitative study of psychological distress, impacts, and adaptation
Source: Front Psychiatry. 2026 Jul 22;17:1878913. doi: 10.3389/fpsyt.2026.1878913 (PMC13437614; doi:10.3389/fpsyt.2026.1878913)
Supplement: Supplementary file 8 [file Table15.docx]

Interview Transcript (N15)

Q: During the hospital or work changes you have experienced, which changes have made you feel pressured or challenged? Can you give one or two examples?

A: Recently we had a nursing quality reform. Some specific documents were issued. Through these documents, we were required to attend meetings to study, then convey the content to the department, and then we were asked to memorise it. After memorising, we have to take exams three times a week on the content of this reform. We have to keep memorising, and leaders also come to spot‑check us. So I think this change is quite stressful for me.

Q: What you memorise--is it something like a nurse professional handbook?

A: Yes, similar. It's a document issued by the Health Commission to the hospital.

Q: This didn't exist before the change, right?

A: No, it didn't. This is a completely new change.

Q: How long has this change been implemented?

A: About half a month since it started.

Q: What was your first reaction when you received this change?

A: At first, my heart sank. I thought, isn't this just causing trouble? We're already tired enough from work, and now we have to memorise all these rules and regulations – three exams a week–who are they trying to kill? I'm not good at memorising things. We nurses are running around after patients all day–how can we find so much time for rote memorisation? It felt like they were just adding to our frustration. I was quite annoyed. And it requires us to keep taking exams, and after the exam we have to review–a repetitive cycle. Sometimes after a busy day at work, I am so tired that I just want to fall asleep as soon as I lie down, but I still have to force myself to stay awake and study–it's really painful. And before every exam, I am very nervous, afraid of failing and being criticised by the leader. Also, when leaders come for spot‑checks, I feel even more panicked. Work pressure is already high, and having to deal with these extra study and exam demands makes my whole spirit tense. I know it might be about improving nursing quality, but this approach feels really hard to accept. Many of my colleagues share the same feeling – we complain privately. If you fail the exam, your salary will be deducted.

Q: The reform has been implemented for half a month now. As it progresses, how has your attitude changed?

A: At first I was quite resistant, because I really didn't want to memorise. And if I memorised wrong or failed the exam, it would affect my whole year's evaluation,things like performance pay, excellence awards, etc. That would impact me. Then as time went on, I became more proficient with the content. As I became more familiar, I felt less anxious.

Q: Is this a hospital‑wide change or just for your department?

A: It's a hospital‑wide change. So no matter which department I go to, I have to go through this ,there's no escape.

Q: In the process of coping with these changes, what personal resources are more likely to cause you physical and mental fatigue?

A: To be honest, these changes do improve my professional skills or competence, because they are applied clinically, so they are quite practical. But the time to learn them is very tight. On one hand, the workload itself is already quite heavy; sometimes I can't even finish my core tasks and have to work overtime. Then after work I have to worry about this. And the exams are never during working hours–they are after the nurses finish their shifts. So it consumes a lot of my personal time. Under the double pressure of work and study, I become especially prone to fatigue. Sometimes I have to stay up late to study after work, then get up early the next day to continue working. This long‑term daily schedule leads to severe sleep deprivation and very poor mental state. And I can't change this situation, so I feel a sense of helplessness. I just try to adjust myself and adapt to the change.

Q: That really does take up a lot of personal rest time. Before the reform was implemented, did the hospital give any notice?

A: No. It was very sudden,they announced the change, then started implementing it, then made demands on nurses. Then we had to start preparing for exams. And there was a required passing score,you had to reach a certain mark to pass. If you fail, either you retake or you lose money.

Q: From the first announcement of the change to the first exam, how long was the interval?

A: About one week.

Q: That is indeed rather rushed.

A: And it's not just online exams,we have enterprise WeChat. After the online exam, there is also an offline exam.

Q: Is the content the same?

A: No, it's different. Because the knowledge is updated, the way questions are asked changes ,the hospital uses various question formats.

Q: Is there any interval between exams?

A: No interval. It's quite random,you might be notified and then the exam starts half an hour later.

A: Not only this – because at the end of the year there are many things, like annual reports and year‑end summaries, and then the hospital adds this exam,it feels like too much to prepare for.

Q: What thoughts arise,for example, do you feel anxiety?

A: At first I was quite resistant, but I had no choice, so I memorised while feeling resistant memorising while depressed, memorising while anxious. Because I knew that if I didn't pass the exam, it would affect my performance pay and awards. Before every exam, I feel particularly anxious, worried that I might fail. Also, the uncertainty of exams that could happen any time keeps me in a constant state of tension, my nerves always tight. Sometimes I even dream about exams, and when I wake up my mood is very heavy.

Q: When you already feel change fatigue in the process of coping with changes, how does that affect your view of subsequent new changes?

A: It's like the story of the boy who cried wolf you keep being told that change is coming, and eventually you get used to it. You lose any freshness. You become indifferent "whatever, do whatever you want, I don't care, I'll just follow orders". I don't even care how much real effect the change will bring. Even if there are new changes later, I probably won't have the same strong emotional reaction as at the beginning. I won't resist or feel anxious as before,I'll just mechanically complete the requirements. I might lose some of the proper enthusiasm and initiative towards work changes. It's not just me ,my colleagues have similar feelings. Everyone has been worn down by these frequent changes. This overall negative attitude spreads in the department. Sometimes I wonder is this state really good for the hospital's development? The original intention of the hospital's continuous reforms must be good to improve the quality of medical staff and services. But if the implementation methods are unreasonable, leading to change fatigue, it might backfire. It might even cause talent loss, because no one wants to keep working in such a high-pressure and exhausting environment.

Q: So it's a bit of a slacking-off mindset?

A: Yes.

Q: How does this mindset affect the quality of your implementation of reform measures?

A: Because there are too many exams, everyone generally goes through the motions. People just aim for a passing score,no one thinks about excellence or being the best. Just passing is enough.

Q: How do you usually relieve the negative emotions that arise?

A: I usually watch TV dramas. I blank out I try not to think about these things, or do something else to distract myself.

Q: Does the hospital provide any psychological support to help nurses relieve physical and mental problems?

A: Our hospital has dedicated psychological counsellors,you can make an appointment by yourself. But I have never made an appointment or participated. Because I feel that if you go, your inner thoughts will be exposed. It's not just telling one psychologist I think the whole hospital would know. Because if you really have a problem, they would probably report it to the leadership. So I'm not willing to go. I prefer to find my own ways to regulate my mood. Besides, I feel that even if I go for counselling, it might not solve the real problem. I can't guarantee the effectiveness, and I would also risk privacy leaks.

Q: During hospital changes, what is your view on a stable period?

A: From my understanding, I think everyone looks forward to a stable period. Stability means a relatively calm state. Then you can just complete your daily work without having stress reactions from ad-hoc tasks. Your body and mind can relax. And you can use that time to do things you want to do,for example, learn new knowledge to improve your professional skills, or cultivate hobbies to enrich your life. During the stable period, the work rhythm is relatively regular, and you don't have to keep your nerves tense like during change periods, worrying about new assessments and tasks at any moment. You can get enough sleep, and your mental state will be much better. The atmosphere among colleagues will be more relaxed and harmonious. I think the stable period for us nurses is like being able to stop and rest during a long run adjust your state, then plunge into the next round of work or change with a better attitude. I think it's a good phase.

Q: How long do you think the stable period should be?

A: Personally, of course I hope it is as long as possible. I don't like changes very much. I think work is for a better life work should cover my expenses. I prefer to focus more time on myself.

Q: What situations do you think would further aggravate your change fatigue?

A: Let me give you a simple example. Suppose we are implementing a system reform, but before this task is completed, a leader suddenly comes for an inspection. Then we have to make corresponding preparations. At that time, you are required not only to manage this but also to practice your clinical skills and consolidate your theory. Because the leader will not only check the implementation of the system but also test your skills and knowledge. When multiple things are happening at the same time, I feel overwhelmed, with a heavy sense of fatigue. I feel very tired overall. If this situation occurs frequently, it makes me more and more resistant to changes.Because you already need time and energy to adapt to new systems and requirements, and before you've recovered, you have to cope with inspections.I feel like I'm always being pushed along with no chance to catch my breath.Also, if new changes keep emerging and the content and methods of changes lack rationality and scientific basis, it will further aggravate my change fatigue. I will feel exhausted and my enthusiasm for work will be worn away.

Q: What factors do you think make it easier for you to feel fatigued when facing changes?

A: I think the frequency of changes affects me more. For example, a system is promoted for a month, and then after a month it is overturned and replaced with a new system. You have to keep adjusting. Sometimes a habit has already formed, and it's very hard to change. I often forget the new requirements. If you forget, your salary is deducted. No choice – you have to adjust your habits very reluctantly.

Q: As a clinical nurse, what aspects do you think researchers should pay more attention to when studying nurse change fatigue?

A: I think we should pay more attention to the psychological state and emotions of healthcare staff. I think if changes are to be made, corresponding reward systems should be established. You can't just let us give without any positive incentives.For example, if I do well under the new regulations, I should be rewarded with something, or if I actively cooperate with the leader and successfully get through the change period, should I be given a period of time to rest? You can't just let us give without any positive incentives.

Q: During the implementation of hospital changes, how has adapting to these changes affected your daily life?

A: The most obvious is that my rest time has decreased. I used to get up at 7 am every day, walk a few minutes to work, and leave at 5 pm. But because of the hospital's changes, I now have to get up at 6 am to prepare for the day's work and exams, and sometimes I can't sleep until 12 pm.

Q: Is there anything else you would like to add about change fatigue?

A: That's all from me.

Q: Thank you for your cooperation.
