# Supplementary material for: Nurses’ experiences of change fatigue during healthcare reforms: a qualitative study of psychological distress, impacts, and adaptation
Source: Front Psychiatry. 2026 Jul 22;17:1878913. doi: 10.3389/fpsyt.2026.1878913 (PMC13437614; doi:10.3389/fpsyt.2026.1878913)
Supplement: Supplementary file 9 [file Table2.docx]

Interview Transcript (N2)

Introduction: Let's begin this interview. First of all, this is a semi-structured interview, so please feel free to say whatever comes to mind.

Participant:OK.

Q: What types of changes have you experienced in your clinical work? For example, process optimizations, technology updates, adjustments in management models, etc. Please describe one or two change events that left a deep impression on you.

A: A new orthopaedic specialty group has recently been established. What kind of change would you call that?

Q: That would count as a readjustment of the work model.

A: I suppose so. A new specialty group was set up—a limb tumor specialty group. Previously, orthopaedics did not have this specialty group; there was only spinal tumors, but limb tumors were not offered. This time, a limb tumor specialty group was added.

Q: Do you currently work in that department? Or did the surgical department just open this specialty group without involving you much?

A: The entire orthopaedic department added a new specialty group, and this limb tumor specialty group is located in the ward where I work.

Q: So essentially, your original work environment has changed. You had never been exposed to this area before, but because the specialty group was established, you are now required to care for patients with limb tumors.

A: Yes. Since it is newly established, and the chief physicians were recruited from other hospitals, everything is new. We must follow the requirements of these limb tumor chief physicians and constantly learn a great deal about limb tumors.

Q: How do you feel during this learning process? After all, the work model is undergoing a new adjustment.

A: First of all, this field is certainly unfamiliar to me. Everything—including the colleagues I work with and the direction of the work—is completely unfamiliar. Previously, I had never been exposed to this. For other orthopaedic specialty areas, such as trauma, I had a lot of exposure and already knew how to care for those patients. Now with limb tumors added, I feel: first, it is unfamiliar, I lack knowledge in this area, and I am still in the learning process; second, it is a rather special disease type. Moreover, the chief physicians' requirements are certainly very different from those of previous specialty groups. So at work, first, because it is unfamiliar, you feel that work is not as smooth as usual. Second, whether in terms of nursing or attention to chemotherapy drugs, there is much more to focus on, and more things to worry about and pay attention to. As a result, work feels more burdensome and troublesome.

Q: When work becomes more burdensome and troublesome, how do you assess your ability to cope with these changes?

A: I just push through. The department is also organizing some educational activities—for example, specialist teachers from other hospitals' tumor units, or from our own hospital. Since it also involves the care of PICC lines and implantable ports, specialist nurses are invited to give small lectures. The hospital has also held relevant lectures on chemotherapy drug knowledge. Everyone is learning through these means.

Q: So for the newly added work, you cannot really assess it well. It is more like learning as you go and solving problems when they arise.

A: Yes. Right now we are completely at the starting stage from zero to one, still exploring and learning.

Q: In a completely unfamiliar environment—and your colleagues are also being assembled subsequently—there may be issues of building rapport with new colleagues, whereas you had already worked well with your previous colleagues. How does this affect you personally?

A: Possibly because after this new specialty group was added, the number of beds in the ward also increased, so nurses like us came from various wards to support. The result is that everyone is no longer doing their original work. Moreover, it is a newly opened ward, so the work model is not the original one either. Everyone—from leaders to colleagues—is slowly building rapport at work. Whether it is the care of patients under this new specialty group or the mutual building rapport among new colleagues, it is a gradual building rapport process at a new stage.

Q: Does this make you feel very stressed? You were working smoothly in another department, and now in a new department—leadership, professional knowledge, collaboration with colleagues—everything has to start over.

A: Yes, the pressure is very high, and in some aspects I feel helpless. After all, everyone has different work habits, and each department has its own requirements and work habits. I constantly need to adjust myself, depending on what habits emerge from the building rapport among colleagues.

Q: When you feel stressed, how do you relieve it?

A: First, minor frictions at work are inevitable. Then, it takes time—gradually building rapport through time and work interactions. Personally, I usually participate in some recreational activities, such as watching dramas and singing, to relieve my stress.

Q: So you take some recreational activities to release stress.

A: Yes. Actually, the hospital also organizes psychological courses, but they are scheduled according to the hospital's overall timetable. For example, several sessions of psychological adjustment courses are offered, but that does not mean all clinical nurses have the time to attend.

Q: Have you attended such a course?

A: I think I attended one online, but I didn't listen for many minutes.

Q: So you feel that some measures taken by the hospital are not very practical for you. One reason is that the timing is not suitable; another is that if it is an online course, the effectiveness is somewhat compromised.

A: Yes. The hospital does not make it mandatory, and the timing is often inconvenient—for example, during rest time or at lunch. If you are on a consecutive shift and working, or if you are on a regular shift and have a lunch break, you are already tired from work and really want to rest. Since online courses are not mandatory, the effectiveness may be poor.

Q: So, for you personally, when negative emotions arise, the methods you choose mostly rely on your own self-regulation.

A: Yes. Basically, it is just internal digestion through personal mindset and time.

Q: Does it have any effects on your physical health?

A: I feel tired and feel job burnout. I think the professional term is "burnout period."

Q: So you feel very exhausted. Can you describe it specifically? How does it manifest, and how do you realize that you are indeed in a job burnout period?

A: First, I definitely reject the work. I don't want to go to work; I feel afraid and resistant to going to work, especially resistant to working the shifts in this new limb tumor specialty group.

Q: Anything else? Do you have tendencies toward anxiety or other emotions?

A: Yes. This state becomes particularly strong when approaching work. For example, thinking about having to work the next day tonight makes me start to feel anxious, and the more I think, the more anxious I become, unable to sleep.

Q: So these emotions may lead to some physical and mental reactions, such as insomnia, right? How long does this condition last? Is it persistent or intermittent?

A: It is persistent. Now, whenever I think about going to work—looking at the schedule and seeing that I have to work—this situation occurs.

Q: How long have you been in this new specialty group?

A: The specialty group officially started in November, let me check when exactly, because it is newly established.

Q: So you have actually been working in the new work environment for less than two months, and you have already developed obvious resistance, as well as some anxiety and negative emotions.

A: Yes, less than two months. The specialty group only began admitting patients in early November.

Q: Apart from this change, have there been any other changes in your work?

A: Perhaps changes between departments. Our orthopaedics department has eight wards. I was originally in one ward and was rotated to another ward in September, where the leadership and colleagues were also different.

Q: So you were transferred to a new department in September, and then in October you were transferred to another new department?

A: No. I was rotated to a new ward in September, and then in November, within this new ward, a new specialty group was added.

Q: I see. So that means you experienced continuous changes over a short period. During such frequent, continuous changes, what thoughts come to your mind?

A: I want to quit. I don't want to do this anymore.

Q: When did such thoughts of quitting roughly appear? Did you have such thoughts in your previously familiar work environment?

A: When I was in the department three months ago, it was okay—no particular thoughts like that. After being transferred to a new department, especially after the new specialty group was established, the pressure of night shifts is so high that I have thoughts of resignation. Every time I have a night shift, this thought pops up.

Q: So after you arrived at this new department, thoughts of leaving appeared and became increasingly intense and obvious.

A: It may also be because the newly opened hospital campus has more beds, but fewer nurses. The workload has increased sharply, pressure is high, and on top of that, a new specialty group has been added. Everything is unfamiliar, and I have to worry about learning new knowledge. So I am very anxious. Now, every day, all I think about is whether I can leave work on time.

Q: Do these emotions and thoughts affect your work?

A: Sometimes they may affect my emotions at work. Because I cannot suppress my negative emotions, I may exhibit some unprofessional behaviors.

Q: So they do have an impact on the quality and outcomes of your work.

A: Yes. Bad emotions create a vicious cycle. Because you care for patients with emotions, and with heavy workloads, when busy and unable to control emotions, patient feedback is not good, which in turn makes your own emotions worse, forming a vicious cycle.

Q: During the process of hospital changes, do you hope for a period of stability?

A: Of course, certainly. Of course, whether the hospital adds a new specialty group is something we cannot decide. But I hope that after gradual building rapport and familiarity, things can be more stable. I hope that every day's work is routine, so that I generally know what to expect.

Q: The broader environment, such as the hospital's future plans, is something ordinary employees cannot control. But you hope that after you have worked in an environment for a while and become familiar with it, you will not be transferred to an unfamiliar environment again.

A: You could say that. If it is stable, and your work goes smoothly, and colleagues have built rapport well, of course you hope it stays stable. Frequent transfers require frequent adaptation, especially for nursing work.

Q: If there were a stable period, how long would you hope it to be?

A: I would hope for at least one year. Otherwise, with such frequent transfers in a short period, personal absorption and learning take time. Learning new things also takes time.

Q: Did you estimate this stable period based on your own capabilities?

A: Probably based on my previous work experience—the time it takes to become relatively familiar with and master the work situation when going to a new place to learn a new specialty group. That is based on my past experience in learning specialty knowledge.

Q: So it is an estimate based on past experience. In the above conversation, you repeatedly mentioned increased work content and difficulty. How do you view the changes in your work caused by hospital changes?

A: First of all, in nursing work, adding new things is unavoidable, and it is also a good development, because nursing is a profession that requires lifelong learning—you certainly need to learn new content. However, it also brings increased workload and more clinical requirements. Sometimes when you are too busy, bad emotions arise, which certainly have an impact and affect your work. I am not opposed to accepting new things or new knowledge; what I resist may be the increased workload or other negative emotions from patients or doctors. Increased workload leads to inadequate physical rest, and the body becomes very tired. That may be what I resist, not simply rejecting the new things or new knowledge themselves.

Q: So what you resist is not the change itself, but the negative impacts that change brings. If it were a positive change—for example, making your work processes simpler and more convenient—then you would welcome that kind of change.

A: Yes. If it truly reduces some cumbersome or unnecessary processes at work, I would certainly accept it gladly.

Q: But during the process of change, it is hard to truly evaluate whether the outcome is positive or negative.

A: That probably requires frontline clinical staff, through real hands-on practice, to evaluate whether a change truly brings benefits or whether its negative aspects actually outweigh the positives.

Q: Is there anything else you would like to add regarding this topic?

A: Not for now.
