# Supplementary material for: Nurses’ experiences of change fatigue during healthcare reforms: a qualitative study of psychological distress, impacts, and adaptation
Source: Front Psychiatry. 2026 Jul 22;17:1878913. doi: 10.3389/fpsyt.2026.1878913 (PMC13437614; doi:10.3389/fpsyt.2026.1878913)
Supplement: Supplementary file 10 [file Table3.docx]

Interview Transcript (N3)

Q: What types of changes have you experienced in your clinical work? Please describe one or two events that left a deep impression on you.

A: There was one change. In my previous unit, there was a shift from a functional system to a primary nursing responsibility system. At first, the change was not very noticeable, but later it gradually became evident. Previously, we worked in a surgical department with a requirement for adverse event reporting. Under the functional system, reporting was like in the ICU—the entire hospital reported as a whole. When an adverse event was reported, the names of all nurses in the department would appear on the adverse event report form. But under the primary nursing responsibility system, only one name appears—the name of the responsible nurse. With this system, the ultimate responsibility falls on an individual, so there is no finger-pointing—no one can say "it was also his fault" or "he didn't do it." I think the primary nursing responsibility system is better. Second, regarding surgery in our department, the previous process was: the operating room nurse would notify us, then we would notify the patient, take the patient to the operating room, conduct the handover, and then the operating room nurse would notify the doctor. But a problem arose: the operating room nurse forgot to notify the doctor. Our department had followed the initial process correctly, but the patient waited in the operating room waiting area for a long time, and no doctor came. After this issue occurred, the process was optimized: the operating room nurse now notifies the doctor, and we also notify the doctor when we send the surgical patient up. This created a double safeguard, and after that, the situation of patients waiting in vain never happened again.

Q: Based on these two changes, do you think the hospital's process optimization changed things in a positive or negative direction?

A: Definitely a positive direction. At first, I did not understand why we needed to go the extra mile and call the doctor ourselves. But in fact, it provided a double guarantee, ensuring patient safety and preventing patients from waiting so long. At the very least, it increased patient satisfaction.

Q: But it did add an extra step to your work. Previously, you did not communicate directly with the doctor, but after the change, you also need to notify the doctor, increasing your workload.

A: But for the sake of patient safety, it is not a big problem—just one more phone call.

Q: So for positive changes, even if they increase workload, they are still acceptable.

A: Yes, that's right.

Q: When these changes were about to be implemented, how did you assess your ability to cope with them?

A: I accepted them very easily. After all, it optimized the entire process and brought benefits to patients, so I was quite receptive.

Q: When a change brings increased work difficulty or adjustments in work content, how do you assess your ability to cope with it?

A: First, before such a change, there must be some advance notice, so you have to mentally prepare. Then, based on your own abilities, if you are not particularly strong in a certain area—for example, if your theoretical knowledge is lacking—you study from textbooks. If you feel your clinical skills are not good enough, you use your rest time to practice more. For things you do not understand, you ask more competent or experienced nurses for guidance. Gradually, your personal ability will improve, or you will accumulate experience in the new work over time. First, you need to be confident and have the right mindset: "I will definitely become a very capable nurse." You also need to combine your own experience and abilities to comprehensively assess whether you have the capacity to cope. If not, then you choose to continue learning. Having a good attitude is very important—mindset matters.

Q: In the process of coping with changes, how do they affect different aspects of yourself?

A: I think that through cumulative learning over time, my own abilities have improved significantly. Although it may take up most of my rest time, at least I can see my progress. Sometimes the process may be painful, but the outcome makes me happy, and it also counts as experience.

Q: So you feel that no matter what the change is, it can be a good opportunity for personal growth.

A: Yes, it is like an experience card.

Q: What about negative changes for you personally?

A: In my previous unit, we experienced a salary adjustment. The most direct impact was staff turnover. For example, previously you might have done the work of one person; now the work of two people is assigned to one person, or the work of three people is assigned to two people. The pressure and intensity are quite high. People's psychology also changes. For example, your colleague may feel very tired and unable to adjust. They might have been supposed to finish the work, but because their mindset has become slack, they only do 80% of it, leaving hidden problems in inconspicuous places without telling you about them. Then, when you take over the shift, if you are not in a good state, you might not notice, creating potential risks. Second, because the salary has decreased, people's spending may be affected. This is not very obvious for unmarried staff, but it is obvious for married ones. For example, there was a nurse in our previous unit who, after getting married and having a child, changed from renting a two-bedroom apartment to a three-bedroom apartment. Her monthly rent increased by about 1,000–1,500 yuan just for rent. So she worked night shifts very actively to earn more night shift allowance to make up for the salary gap. This led many people to no longer want to work shifts with her, because some felt that with such a mindset, her work quality could hardly be guaranteed—people have limited energy. Working with her required extra caution and worry, making them feel more exhausted. Others felt that since their salary was adjusted, everything else should be adjusted accordingly. One nurse who left used to say before leaving: "I want the hospital to know that cheap goods are not good. You only pay me this much, so I will only do this much work," or she would do nothing at all. This created great psychological pressure for those working with her. So I think this is also a negative emotion. First, there is staff turnover. Second, even among those who stay, their internal thoughts are quite significant.

Q: So if a change has negative effects, it may affect not only you personally but also the atmosphere of the entire department, leading to decreased work motivation.

A: Yes. And that in turn affects you personally. For example, your own emotions may fluctuate. Previously you could buy a 580-yuan perfume, but now with the monthly salary reduction, your purchasing power or desire to consume gradually declines. Moreover, as you age, you get married and have children, so your expectations for the job may not be as high. For example, when payday came on the 7th or 8th, you might have been excited from the 1st. But when you know your salary has been reduced every month, your expectations for payday are not as high, because you feel it is just that little amount anyway—it doesn't matter whether it is paid or not. This greatly affects your mood. Some people may come to hate payday, because after getting paid, you take home so little money, your parents may be unhappy, and your family may say: "You work so hard, you have no rest time, and you only get paid so little." Some may even feel that not getting paid would cause fewer conflicts.

Q: What thoughts do frequent hospital changes bring to your mind?

A: First, to give a simple example: when I left my previous unit, a nurse who started at the same time as me also said he wanted to leave. As far as I know, he planned to work until the end of the year and then leave. Before I left, a very good work partner of mine also left. So, when people you work with leave, it may affect your good friends or also give rise to such thoughts.

Q: Did they leave because of the previous salary adjustment?

A: Not entirely. It may also be due to age and personal circumstances. After all, in a first-tier city where housing prices are so high, people are nearly 30 years old. It may not be only the employer's fault; environmental factors also contribute to staff turnover. But a large part of the reason is likely the salary change.

Q: After they left, did that give you other thoughts?

A: Yes. Our profession is already very stressful and oppressive; we are busy every day. After they left, their social media posts were all about traveling and enjoying themselves—going here and there, spending money here and there. That gives you certain thoughts. It feels like after leaving, they become so free and happy—not just confined to one place. They can go home, live with their parents, live with their friends and relatives.

Q: So their behavior leads you to have such thoughts as well. How does that affect your work?

A: It does not affect my work. This comes down to basic professional ethics. We still have a work handover. If you do not hand over clearly and do not complete your own responsibilities, you cannot leave.

Q: How does it affect your work mindset?

A: It does. I might secretly smile to myself: "I am going to resign; I feel happy." But work is still related to personal professional integrity. In our previous unit, you had to submit a resignation application one month in advance. After submitting it, you still had to work until the same day next month. I could not just stop working for a whole month.

Q: OK, understood. During the hospital's development process, do you hope for a period of stability?

A: Yes. I hope that after a major change, there will be at least a period of time for everyone to adjust and adapt. People need to work together and coordinate with one another; we can’t expect everyone to accept the change immediately—there needs to be at least a period of stability. A major change is fine, but you cannot expect everyone to accept it immediately. At least half a year, or for a particularly major change, one or two years of building rapport may be needed. For example, if my salary is reduced by a certain percentage this month, then reduced again next month, and then again the month after, I would be working for nothing. Whether in terms of frequency or magnitude, it is hard to accept. Adjustments can be made as times require. For example, in our previous hospital, the educational requirement for nurses was only a junior college degree. Later, with the development of the times, a bachelor's degree became acceptable. But you cannot say that this year you require a junior college degree and next year only a bachelor's degree, and not only stop recruiting junior college graduates but also dismiss existing nurses with junior college degrees. That would certainly not work. You can give people a period of time to upgrade their education, which takes three or four years—a transition period.

Q: So you think change is inevitable, but during the process, you hope that major changes proceed more slowly and that there is a buffer period.

A: Yes. It needs to be acceptable to people. Everyone's ability to accept change is not the same.

Q: How do you define the size of a change? For you personally, what is a major change and what is a minor change?

A: A major change would be transitioning from a second-class hospital to a third-class hospital, undergoing the hospital grade assessment—I consider that a major change. Or a sudden change in work hours would also be a relatively major change. In my previous unit, we had a 12-hour shift system divided into two shifts. If it suddenly changed from two shifts to three shifts, or from three shifts back to two shifts, that would involve manpower cost factors. Some changes, such as those that are implemented immediately after being announced and do not require a long adaptation period—maybe just a meeting to explain, and everyone quickly adapts, or later someone who missed the announcement is told privately, and after that they adapt—these are minor changes.

Q: OK, understood. Is there anything else you would like to add about nurse change fatigue?

A: Not much to add. Change is certainly necessary. The goal is always to make things better—to make the environment, human resources, and work atmosphere positive and upward, not worse. Leaders need to consider the thoughts of frontline staff when making changes.

Q: So you are not resistant to change itself, but to some of its impacts. If the impact is positive, you will have a positive attitude. If the change makes you less able to adapt, you try to adjust your mindset and work hard to adapt.

A: If I cannot adjust, I might reformulate other career plans. I would first see if I can adapt to it. But with age, family factors, or the social environment, if I cannot adapt, I might consider whether there is another job where I could be happier. With income not too low, I might choose a happier lifestyle.

Q: In the process of coping with changes, what kind of emotions do they evoke in you?

A: When you cannot cope, negative emotions certainly arise. Everyone has emotions. For example, if a sudden problem arises at work, or something difficult to solve—where before the change, you could handle the problem better, but due to some hospital decision, the work becomes more complicated, harming without benefiting—after the change, things are not optimized but become more cumbersome. That makes me feel quite irritated. And these are things you have to face every day. For example, in my previous unit, there was a regulation that medical staff could not take the elevator within three floors up or down. In the morning when you are in a hurry, even without patients, you cannot take the elevator for three floors. That is somewhat unreasonable. It takes you three minutes to get there, but you have to walk up three flights of stairs. That is certainly tiring. After a night shift, I am already dizzy and blurry-eyed, yet I am not allowed to take the elevator for three floors down. That feels unreasonable, and I feel very resistant to such a newly issued regulation.

Q: What kind of emotions does that produce?

A: For us young people, it might be okay. But for a director in their fifties or sixties, not being able to take the elevator for three floors—how is that reasonable? So you complain. Some retired experts are in their sixties or seventies, and they cannot take the elevator either. What if they fall? It is completely unreasonable. They are sixty or seventy years old and may have worse physical condition than some patients.

Q: Indeed. So your attitude toward change depends on the type of change and its impact.

A: Yes. Change is acceptable, but do not make chaotic changes—do not come up with strange, unreasonable things.
