# Supplementary material for: Nurses’ experiences of change fatigue during healthcare reforms: a qualitative study of psychological distress, impacts, and adaptation
Source: Front Psychiatry. 2026 Jul 22;17:1878913. doi: 10.3389/fpsyt.2026.1878913 (PMC13437614; doi:10.3389/fpsyt.2026.1878913)
Supplement: Supplementary file 11 [file Table4.docx]

Interview Transcript (N4)

Q:What types of changes have you experienced in your clinical work? Please describe one or two specific change events that left a deep impression on you.

A:Do you mean changes promoted by the hospital or changes promoted by the department?

Q: Either is fine. As long as it changed your previous stable work state, it can be included in the scope of this study.

A: My hospital is relatively stable with few changes. Some time ago, we got a new leader who required us to keep records of admissions, patient conditions, department problems, and related plans. One person was assigned specifically to work on these plans. More recently, we have been promoting an internet-based service—home visit nursing services arranged online.

Q: So after the new leader took office, there has been a clear change in the division of labor between you and your colleagues.

A: Yes. And with the recent promotion of this internet service, we have moved away from manual record‑keeping. Now everything must be archived on the computer, and we have to attend offline training sessions each time.

Q: So after introducing the internet service, you changed from paper records to electronic records.

A: Yes.

Q: What specific impact have these two changes had on your work?

A: The workload has increased, and with one less colleague, the burden is heavier. Second, record‑keeping has become more troublesome—you have to write things down manually first, then enter them into the computer. The work process has become longer.

Q: So compared with your previous way of working, the workload has increased and it is more troublesome, right?

A: Yes. Also, with the offline service, we need offline training, so we have to attend training sessions.

Q: Could you elaborate on the training? Are you responsible for the entire training?

A: No, I am not responsible. I am a learner, and there is a dedicated trainer.

Q: So this also increases your workload?

A: Yes. The training has to be done offline.

Q: It must be offline; online is not acceptable? So it may take up your personal rest time.

A: Yes, yes. For online training we also have to attend sessions. So I have less rest time than before.

Q: After the leadership change, we also have weekly operation exams and theory exams.

Q: Every week? That did not exist before, right?

A: Yes, it did not exist before.

Q: So that adds an exam every week, which is quite a high frequency.

A: Previously we did not care much about such things. Some hospitals do not have these exams, so we did not pay much attention to skills training. This year, the workload feels heavier and the training has become more professional.

Q: So these changes have brought adjustments in both workload and work difficulty. In the process of coping with them, how do you assess your ability to handle change?

A: I certainly know that my ability is insufficient. So I take time out, sacrifice my rest time, and work hard to get things done. Because there is an assessment system—if you fail an operation or theory exam, you may face fines or criticism.

Q: So it is linked to your performance pay?

A: Yes.

Q: So even if the change is difficult for you, you still have to solve the problem, adjust your ability, and adapt to it; otherwise, there are punishment mechanisms.

A: Yes. I have no choice but to complete it.

Q: Had you heard of or been exposed to internet services or similar work before this?

A: Our current exposure to internet services is basically making PowerPoint slides and filling out forms. The internet service started being promoted in the second half of this year. At the beginning, I did not have much exposure, but the requirement was just to input and output some forms, which is relatively simple.

Q: So you had little prior exposure, but the difficulty for you personally is not that great.

A: Yes, that is right.

Q: When you received this work adjustment, what was your first reaction? What was your first thought?

A: My first thought was resistance. Then I gradually adapted. After several rounds of meetings and discussions, I finally had to compromise.

Q: So first resistance, then gradual adaptation, and finally passive acceptance.

A: Yes. No other choice.

Q: Regardless of emotional changes or workload adjustments, how do these affect you personally?

A:Rest time has significantly decreased, I feel very tired, have no more time to accompany my family, and no time for leisure and entertainment.

Q: So it makes you feel physically exhausted?

A: Yes.

Q: How do these reactions affect your daily work?

A: Nursing work is already quite tiring. It requires a lot of responsibility. With these changes, negative emotions will certainly arise.

Q: How does it affect your daily communication with patients?

A: It does have some effect. Sometimes when things are urgent and work is busy, I may not be able to pay enough attention to patients' attitudes. If someone comes to ask something, I might not explain it very clearly, and that then affects my own work mood.

Q: During these hospital changes, do you hope for a stable period? For example, within half a year, you have added internet services, added training, added exams—in this process of continuous, frequent changes, do you ever wish the changes would slow down so you could adapt first?

A: Our hospital is quite democratic. We can discuss things with the leaders and talk about how things should be done. It may not be implemented directly; there might be some negotiation. But the time required for implementation is quite fast—for example, the leader may ask for completion within half a month or one month. Although our opinions are not very important, being able to speak up makes me feel slightly better psychologically.

Q: If there were a stable period, how long would you personally hope it to be?

A: At least half a month to one month—15 to 30 days.

Q: How did you estimate that timeframe?

A: Based on my own emotional adjustment and communication with my family. Because if work takes up more time, naturally I have less contact with my family.

Q: Do you feel that after about a month, your family would gradually adapt to your new work pattern? And after you communicate with them and receive their understanding and care, you personally would find it not so hard to accept?

A: Yes. With family support, it becomes better.

Q: So for you, family care and support are quite important.

A: Yes, that is right.

Q: If you were placed in a completely major change—your workload increases, the patient population you care for is completely unfamiliar, and your colleagues are all new so you need to build rapport from scratch—how would you assess your ability to cope with such a major change?

A: Being in a new environment makes me feel a bit scared. If the building rapport does not go well over time, work will not be very efficient. If colleagues' attitudes are not very friendly, the hospital environment is unfamiliar, and I do not understand the procedures well enough, I may develop resistance. But deep down, I want the change.

Q: So internally you also want to grow more, but during implementation there are inevitably things you do not understand, areas where you are weak, and that leads to negative emotions.

A: Yes. Personally, if there are technical aspects I do not understand or operations I do not know, I can ask others—that is fine. But the unfamiliarity that comes with not knowing colleagues well creates worry and fear.

Q: When such negative emotions arise—worry, fear, relatively negative feelings—how do you adjust and relieve them?

A: I talk to my family, or I lie awake at night thinking about these things—just digest them myself.

Q: What recreational activities do you use?

A: I go out with friends, chat, have some barbecue skewers, play games to vent.

Q: Regarding the changes promoted by the hospital, what measures are in place for psychological support of employees?

A: There is no psychological support. Maybe a bit more money—if the hospital suddenly introduces a change that increases the workload, the salary might go up a little.

Q: So there is some material compensation.

A: Yes. Maybe the salary is two or three hundred yuan higher than before.

Q: So whether material or spiritual, if some compensation is given, as long as it is within what the individual can accept, the change is acceptable.

A: Yes. But nursing work is already too busy. No matter how much material help you give, the body cannot take it. It is really too busy. Our department is relatively better. In other departments, people simply cannot leave on time. They say it is an eight‑hour workday, but in reality, every day it turns into ten or twelve hours. Then we have meetings every day after work.

Q: So your hospital is relatively busy. The workload is already high. Then the hospital implements some changes, adding even more workload. For you personally, even if some material compensation is given, your inner thought is: "I would rather not have the extra money and just have a lighter workload."

A: Yes, exactly.

Q: For example, if a change does not increase your workload but actually reduces it, making your work processes more efficient—what would be your personal attitude toward such a change?

A: I would very much hope for that.

Q: So you would accept it willingly, and you hope for positive changes, not changes that increase your workload and make you more tired.

A: Yes. I very much hope for such changes.

Q: In all your years of work, have there been more positive changes or negative changes in the hospital?

A: More negative changes. Positive ones are not many.

Q: Before a change is actually implemented, you do not know whether it will be positive or negative. Then during implementation, you assess what impact it brings, right?

A: Yes. Sometimes when a policy first comes out, it may have a negative impact. But after you become skilled at it, you feel it is a bit better than before—more convenient, easier.

Q: So at the beginning, for some changes, you cannot tell whether they are positive or negative. During implementation, they may be troublesome at first, but as you become familiar with the process and more proficient, you find that they are indeed better than before.

A: That is true for a small number of changes.

Q: During this adaptation process, how does it affect your emotions?

A: It creates some anxiety and stress. Later, when you become skilled, looking back at the measures before the change, you realize that the outcomes and convenience after the change are indeed better.

Q: So at first you may not be very optimistic and may feel forced to accept it, but later you find that it actually reduces your workload, and it becomes a positive change, so your attitude shifts.

A: Yes.

Q: OK. Regarding the topic of nurse change fatigue, is there anything else you would like to add?

A: I think that is about it. Your summary matches what I meant.
