# Supplementary material for: Nurses’ experiences of change fatigue during healthcare reforms: a qualitative study of psychological distress, impacts, and adaptation
Source: Front Psychiatry. 2026 Jul 22;17:1878913. doi: 10.3389/fpsyt.2026.1878913 (PMC13437614; doi:10.3389/fpsyt.2026.1878913)
Supplement: Supplementary file 12 [file Table5.docx]

Interview Transcript (N5)

Q: What types of changes have you experienced in your clinical work? Please describe one or two change events that left a deep impression on you.

A: First, our hospital promoted the process optimization of the trauma center. The emergency department launched an integrated treatment process for severe trauma patients. Before the change, such patients had to wait in turn for consultations from surgery, orthopaedics, and radiology. After the change, it became a multidisciplinary team collaboration. Basically, the team is in place within 15 minutes and provides one-stop emergency treatment. So that left a deep impression. For example, for a patient with a severe car accident, the total time from admission to the operating room was only 20 minutes. In the past, it would have taken at least 40 minutes. So after this alert system, the rapid establishment of intravenous access, team coordination for puncture and blood transfusion-the overall efficiency has improved significantly. The second is the nursing informatics part. For example, the nursing system-all handwritten medical orders have become electronic, and this system is integrated in real time with the laboratory and pharmacy. After informatization, work efficiency has greatly improved, I feel.

Q: Did you directly participate in both of these change events? You work in the trauma center, right?

A: Yes.

Q: When you first learned about these changes, what were your inner feelings?

A: In terms of my inner feelings, because the emergency department already has high work pressure, at the initial stage of the change I felt a bit uncomfortable. Previously, we used traditional methods with a slower pace, but after the new system came, it was indeed a bit difficult to adapt initially. However, we were required to master it completely in a very short time, which gave me a feeling of haste and discomfort. Not only my department was changing; other departments were the same. Everyone had to complete these tasks in a very short time. So at the start, it felt a bit rushed and I was not quite adapted. But after about half a month of operation, although we were still in a running‑in period), everyone felt we had entered a stable phase.

Q: So at first you were not very positive about accepting it, but after gradual running-in you found that the change truly optimized things, and after becoming familiar with the whole process, your attitude became relatively more positive.

A: Yes.

Q: During the change process, what resources of yours were consumed, leading to feelings of fatigue?

A: First, my work content changed. Although efficiency has improved for patients, the workload feels much heavier. One surgery that used to take over 40 minutes now has to be completed in 20 minutes, which essentially doubles the workload. So with the shorter time, the overall work pressure or workload has increased a lot.

Q: The new process doubled your workload. How does that affect your view of future changes introduced by the hospital?

A: I think it does have an impact. I worry about other changes in the future. As I just mentioned, changes in systems and upgrades in nursing informatics do improve work efficiency, but efficiency essentially means you do more things in the same amount of time. So I do have some concerns about future information‑related changes. I worry that, since our department already has heavier workload and pressure than other departments, if the system is updated again or a new system is introduced, it may affect my work time and rest time-especially my rest time.

Q: So you are mainly worried about rest time. If a new change comes and increases the workload even more, then rest time will decrease. Your baseline workload is already high, and the emergency department is quite special with high pressure.

A: Yes, very worried.

Q: When these changes first arrive, how do you assess your ability to cope with them?

A: First, I assess what abilities the change requires. For example, the intelligent diagnosis system I just mentioned requires relevant computer knowledge-which system to use, how to operate it, how to use electronic assessment forms, etc. For improvements in the diagnostic process, you have to complete examinations for patients in a very short time, so you need to adapt to that rhythm. Previously, handling one patient might take 15 minutes; now it might be compressed to 8 minutes. Within those 8 minutes, you have to finish all the work without making mistakes. So at such times, I need to practice more actively-improve my professional skills and other abilities-to avoid errors during these very tight timeframes.

Q: So you assess the situation based on your own abilities and the abilities required by the change itself. If your abilities are insufficient, your choice is also to actively learn and work hard to adapt to the change, right?

A: Yes.

Q: During these hospital changes, how do they affect your own psychological state or work state?

A: First, regarding work state-as I mentioned earlier-there is a short term increase in fatigue. At the initial stage of a change, workload increases, and you have to adapt to the new work rhythm. That state may improve after entering a balanced phase, because you find the pattern or team collaboration runs in better. Second, personally, I have changed from passive execution to more active anticipation. For example, after the trauma process reform, I may prepare the required instrument kit in advance, rather than waiting for the doctor's order before preparing.

Q: So the change process has also trained your personal ability.

A: Yes.

Q: How does the process of adapting to changes affect your psychological state?

A: I think the main emotions in terms of psychological state come from work pressure. After the change, first, workload increases; second, with increased workload, it also involves team collaboration. Sometimes in the emergency department, we have colleagues transferred from other departments. When working together, there may be different rhythms in collaboration, which also has some psychological impact. But I think it is still mainly the emotional impact from work pressure itself.

Q: Does the emergency department have high colleague turnover? Are people frequently transferred from other departments to support?

A: Yes, that happens often.

Q: So that means your colleagues are also constantly changing, and you need to keep building rapport with them. So for you, is it like continuously adapting to the change of colleagues?

A: Yes. For example, you may have just adapted to the team's work rhythm, then after you master it, a colleague is replaced, and you need to find the rhythm again. So you have to constantly adapt and adjust your mindset.

Q: So in the process of continuously adapting-whether to colleagues, hospital processes, or hospital development-if changes happen frequently and are difficult for you personally, what thoughts arise?

A: I think if changes are too frequent in a short period, I feel like I am being pushed along. It is not that I voluntarily learn and then become proficient. It is more that I am pushed. As I just mentioned, whether it is a system or colleagues, just when you get familiar with the rhythm, something new or someone new arrives, and you have to readjust-that feels helpless. That is the negative aspect. The positive aspect is that new system changes definitely represent progress in healthcare, and higher work efficiency is certainly better for patients.

Q: Regarding the new system update you mentioned, does that change make you feel pressured?

A: Yes, I feel some pressure.

Q: Was the entire system completely replaced, or was it updated and improved based on the original system?

A: For the trauma part, it was a complete renewal-completely different from the previous waiting for consultation model. After the change, it is one-stop treatment. I think it is a relatively thorough renewal.

Q: Taking the system change as an example you have been talking about it this change makes you feel pressured. What physical and mental reactions do you experience?

A: I feel a bit anxious. Because the system is completely new and has a lot of content, the time cost of learning is quite high. But the hospital gives you very limited time to adapt. So within a limited time, you have to learn and adapt to the new system. Basically, there is no adaptation period after the change is announced, it is put into use immediately, so your workload increases sharply. Second, it also requires higher professional competence. As I just mentioned, you have to complete tasks in such a short time without making mistakes.

Q: Regarding these changes, does the hospital regularly offer courses or organize group training to help you adapt?

A: Yes, but the training time is not very long. Because the emergency department is different from other departments it is always in a tense state. So when we attend training, it mostly takes up our own rest time. For example, you are on a day shift or night shift, or when you are not resting, they call you to training. So I think for us, basically we have no rest time at all.

Q: Is attendance mandatory?

A: Basically yes, because if you don't attend, you will fall behind.

Q: So that has a big impact on you. The emergency department is already very busy, and then you have to use your rest time to study it feels like working every day.

A: Yes.

Q: During these changes, what emotions or thoughts arise in you?

A: I think if changes are too frequent, I may feel that mentally and physically I cannot bear it – that the change has exceeded my capacity to cope.

Q: Do negative emotions affect your work state?

A: Not really. When we are in a work state, we don't have time to think about that much. Only when we stop do we have thoughts like "I am so tired." So such emotions do exist.

Q: Because you work in the emergency department, which is already high‑pressure, and the hospital introduces various changes, your pressure increases. When you feel stressed, what methods do you use to relax and relieve it?

A: When stress is high, I basically rest and distract myself. Actually, the hospital understands the special nature of emergency nursing work. The hospital also knows that the nursing workload has increased after the reform, so they give a certain increase in income, which makes me feel a bit better.

Q: So the hospital provides some material compensation, which makes you feel more comfortable – not in a situation where workload increases but income does not. Besides material increases, are there other measures?

A: Also, priority consideration for excellence awards – one or two spots per department – and opportunities for疗养 (recuperation) trips.

Q: During these hospital changes, do you hope for a period of stability?

A: I certainly hope for a stable period, because emergency work itself is high‑risk, so it needs a stable period to form memory.

Q: How long would you hope it to be?

A: I think stability should be at least half a year. Major changes should not be too frequent. But minor changes – such as the format of nursing documentation or small departmental adjustments like how to reorganize and classify supplies – those are acceptable.

Q: How did you estimate the length of this stable period?

A: I think based on the磨合 (running‑in) time – when the work rhythm is fully adapted and熟练 (proficient), and the work rhythm no longer has a significant impact on your work or your life outside work – that is how I estimated it.

Q: So it depends on the specific change, how long it takes to磨合 (run in) well, and then you can define the time?

A: Yes.

Q: Regarding nurse change fatigue, is there anything else you would like to add?

A: Yes. I think there are also hidden costs of change, like the process I just mentioned. In the initial stage, it may require training, which takes up our rest time. This hidden cost affects us, and from the hospital management perspective, it may not be particularly considered.

Q: What factors do you think make nurses more likely to feel fatigue when facing changes?

A: First, if the change is a complete, thorough system overhaul, it makes nursing staff feel uncomfortable, especially in the initial stage of learning to use it. Second, for new things, not only do you need to be familiar with them, but you also need to master them – this process is quite difficult.

Q: So over time, these feelings of fatigue gradually decrease, right?

A: Yes. Only after磨合 (running‑in) can the fatigue diminish.

Q: In the process of coping with changes, if you already have negative emotions and feel fatigued, how does that affect the quality of your implementation of coping measures?

A: I think if negative emotions arise, the quality of completion may be somewhat compromised. But not very obvious. It may just lead to lower energy or poorer concentration at work, and the efficiency of nursing care may be slightly slower than the normal process – something like that.

Q: Anything else to add?

A: Nothing else for now.

Q: Thank you for your cooperation.
