# Supplementary material for: Nurses’ experiences of change fatigue during healthcare reforms: a qualitative study of psychological distress, impacts, and adaptation
Source: Front Psychiatry. 2026 Jul 22;17:1878913. doi: 10.3389/fpsyt.2026.1878913 (PMC13437614; doi:10.3389/fpsyt.2026.1878913)
Supplement: Supplementary file 13 [file Table6.docx]

Interview Transcript (N6)

Q: During your work, what types of changes have you experienced? Please describe one or two events that left a deep impression on you and in which you were actively involved.

A: OK. The first change during my work was the launch of our hospital's new system the HIS system. It is a computer‑based work system; all tasks are completed on that system. At the time of the change, I happened to be rotating in another department. I think I was quite involved in this change, because the department I had just joined was a relatively small one not many staff, and most were older nurses. There were not many young people in that department, so I became a kind of main force. I was the department trainer, responsible for training everyone on the system upgrade, how to use and maintain it, and the use of computers and PDAs. After the training, this task was assigned to me. During quieter moments at work, I helped everyone, showed them how to use it, and told them how to operate when they encountered problems. For example, when the system lagged or had bugs, there was a group chat where I could contact and communicate with the hospital's IT group to solve the problems in our department. Another change was related to nursing handover. Many hospitals still have a large handover where doctors and nurses hand over together. Initially, it was a verbal handover. Our three wards used to hand over together orally, with each ward's nurse using their own handover sheet. Nurses handed over, then doctors handed over, and after the leaders listened, they raised any questions; if there were none, the meeting ended. Later, the handover format changed from paper‑based oral handover to PowerPoint presentation. The format changed quite a lot we had to shift from a very traditional method to something very formal and process driven.

Q: Regarding the handover format from paper to PowerPoint presentation did you have to make a PowerPoint every day for the handover?

A: Yes. It had to be very complete and very detailed, including routine discharges, new admissions, critically ill patients, departmental dynamics, resuscitations, etc. All the content had to be made into a very structured PowerPoint for presentation.

Q: Handovers usually take place after a night shift, right? So that means during your night shift, you have to make the PowerPoint and then present it in the morning?

A: Yes. But our model is that the night shift nurse makes the PowerPoint. Then during the handover the next day, if there are no emergencies, the night shift nurse presents it. We also have a team leader shift every day; if there is an emergency, the team leader presents it, and the night shift nurse stays in the department to handle urgent matters.

Q: So that means the night shift nurse has changed from a traditional paper handover to now making a complete, relatively content‑heavy PowerPoint for the handover presentation.

A: Yes.

Q: Did these changes the system change and the handover process change cause you any pressure or challenge?

A: Yes, they did. On one hand, the pressure came from worrying about my own learning ability. In the beginning, the paper handover was very simple. Making a PowerPoint requires a lot of detail it is quite tedious and has certain requirements for the process, format, and use of templates. For example, for a patient who has had major surgery and has many tubes, wounds, etc., typing all that into the PowerPoint takes a lot of time and also demands learning ability. Previously, we only made PowerPoints for case reports or nursing rounds. Suddenly changing to night shift handover also requiring PowerPoints the handover process became very different. So learning and making PowerPoints required more refinement. Another pressure was time. Anyone who has worked in clinical nursing knows that both day shifts and night shifts are very hard, especially in critical departments like surgery, ICU, or emergency. Night shifts rarely have free time. Sometimes you are busy all night without a chance to sit down, constantly running in the wards to provide nursing care, resuscitation, changing IV fluids, taking blood glucose even hourly blood glucose tests. So we have to compress work time while improving speed and efficiency. At the same time, if there are emergencies, the pressure is even greater. You have to handle emergencies you cannot ignore patients. On the other hand, you worry about the PowerPoint and have to find time to make it. It feels like there is not enough time, and the PowerPoint has to be very detailed.

Q: These changes brought you pressure, increased work difficulty, and adjusted work content. How do you assess your ability to cope with these changes?

A: I feel challenged and pressured. After the leadership announced the change, first we had to accept it, then continuously learn according to the leaders' requirements, to better complete the handover and PowerPoint making, so that the new task could be done well. On the other hand, it is about learning ability. I think since these things exist, they must have a purpose and meaning. If leaders want to make changes, from their perspective, they probably think that the traditional paper handover ends as soon as it is read out. After the change, a PowerPoint is retained. Displayed on a large screen, it allows leaders and all handover participants to see the previous day's departmental dynamics more clearly and intuitively. So I think the change has benefits. It also allows non‑night‑shift nurses to better understand changes in patients' conditions and the nursing measures taken the previous day, helping everyone plan their day's work better. For example, knowing in advance about critically ill patients or those needing special care allows for better time management and prioritisation. That is a positive aspect. Another ability required is time management. Since we have to make the PowerPoint on top of our regular duties, we need to adjust our time according to the night shift situation for example, complete urgent and complex tasks first, then less important ones like some nursing documentation, which can be postponed a bit. Finish the PowerPoint first, then after handover, complete the less urgent documentation. This requires good organisational ability. Also, the ability to handle emergencies for example, if a patient has a morning gastroscopy but has not finished their laxative, you need to prepare them for that, and prepare materials for enema. Being well‑prepared allows you to handle things calmly. So for me, learning ability and organisational ability have greatly improved.

Q: We all know that basic nursing work is already very busy. After the change, additional content was added. When you first learned about these changes, what was your inner feeling?

A: My first feeling was that work would become more tedious and harder in the future, and I would have to keep facing these challenges and changes. On one hand, I needed to learn more about department work; on the other hand, I needed to improve other abilities to better cope with these changes. Perhaps I also needed to learn from colleagues in other hospitals that had undergone similar changes, to borrow their better ways of handling challenges, so as to better complete my basic duties and adapt to the change.

Q: As the change progressed, how did your attitude change?

A: From a bit of confusion and unfamiliarity at the beginning, to gradually adapting and learning, becoming proficient, and then feeling more confident and skilled.

Q: During the process of adapting to these changes, what resources of yours do you think were consumed? For example, time spent focusing on patients, trust between colleagues, sense of control over work, or personal rest time these can all be considered your resources.

A: The biggest consumption was probably time. On one hand, you have to spend energy and time learning and coping with the change, and also handling clinical emergencies. When the change was first introduced, everyone was constantly making PowerPoints, requiring manpower for that. At the same time, manpower was also needed to handle departmental situations such as admitting or discharging surgical patients, providing patient care. This could lead to uneven or unfair allocation of staff, which might cause small frictions between colleagues. For department leaders, like the head nurse, they might feel they have done their best in staff allocation, but from colleagues' perspectives, they might be dissatisfied. So small frictions can arise between colleagues, and between leaders and colleagues. Leaders feel they have tried their best but cannot satisfy everyone; colleagues feel that they are always assigned heavier or more demanding tasks, leading to a sense of imbalance. Over time, this may increase departmental conflicts and may not be conducive to the smooth operation of departmental work or harmonious relationships among colleagues.

Q: So in coping with these changes, your emotions are also affected. Could you elaborate on those emotions for example, your psychological state at the time and how they affected your work state?

A: My first reaction was a bit of resistance. I felt that clinical work already left no spare time to meet more demands. On the other hand, I felt a lot of pressure – resistance to change. The second emotion was anxiety. I often suffered from insomnia at night. At the beginning, I felt helpless and even a bit collapsed. For example, when busy, a lot of things would pile up at once, which was very overwhelming. When facing colleagues, I felt I had not done well and was holding them back. Although when things really happen, colleagues still help each other and support each other, those emotions still existed. I just wished there could be fewer changes. Some changes are necessary, but some changes, for us nurses, are just extra burdens. For the whole clinical setting, some tasks do not necessarily have to be done by nurses changes could also happen at other levels, such as medical technology or medical treatment. I feel that changes in nursing might not have much significance. So I would prefer more stability. Regarding changes, I hope there won't be too many just maintaining the status quo would be good.

Q: So you feel that during hospital development, nursing changes are too frequent. You hope for a period of stability to gradually adapt. For you personally, how long would you hope that stable period to be?

A: Yes. I hope for two to three years. On one hand, that gives us enough time to learn and cope with changes, and to apply them better in clinical practice. On the other hand, I think it would also relieve nurses' physical and mental stress a bit, so we are not constantly under enormous pressure. Nurses are the ones with the closest clinical contact, especially with patients. If nurses are constantly in a state of emotional upheaval from changes, it may affect their psychological and physical health for example, insomnia may lead to low work efficiency the next day. If nurses have problems, the first victims may be the patients, because patient care may not be thorough enough. So I hope the period is longer, more stable, and more lasting that would benefit nurses, clinical work, and the entire healthcare industry. On the other hand, if changes are too frequent, it can only mean that each change is not successful enough. If a change is truly successful and can be applied well for a period of time, it shows that it genuinely improves clinical practice. If changes happen too frequently, I feel that each change is meaningless changing again and again, then there is no need for it to exist.

Q: You mentioned that during hospital changes, you may feel anxious, stressed, and experience insomnia. What coping measures do you have?

A: Coping measures from not understanding to understanding, there is a process. At the beginning, you learn more. First, learn together with department colleagues. If there is something you don't understand, ask colleagues for help. If there are unreasonable staffing arrangements, also ask leaders for help and make suggestions for example, how to arrange staffing more reasonably, how to balance workload, so that everyone's workload and psychology are considered, which helps clinical work. On a personal level, practice makes perfect. If you really find yourself in a bad psychological state, such as severe insomnia, you can go out with colleagues on weekends or rest days. On one hand, you can talk about your difficulties; on the other hand, it helps build relationships, so you can cooperate better clinically. smoothly.Also, for example, when dealing with a new system, you can look up new literature online. During a system upgrade, there are many things for example, documentation writing. If we are not proficient ourselves, we can read literature to learn and make our vocabulary more professional. Another thing is self‑overcoming. If it is really hard to cope, you can take measures to help with sleep for example, massage, smelling flower or fruit scents, using essential oils, or listening to soft music to increase the desire to sleep, calm your mood, and fall asleep better, so that you have abundant energy for work the next day.

Q: If you already feel fatigue while coping with changes, how does that affect the quality of your implementation of coping measures?

A: If the change is truly large and has no promoting effect for us, we will first report it to the leadership level report to our direct leader. If possible, hope they can raise it with higher management to see if it can be optimised and improved to make the change more streamlined or more effective. But if, after raising the issue, it still cannot be effectively resolved, then the quality of completion may not be as good. Because I think reforms should promote clinical practice. If they do not promote clinical practice, then their quality may be somewhat compromised.

Q: When you already feel change fatigue, how does that affect your view of subsequent new changes?

A: If previous changes were all successful, then we will gladly accept new ones. If they were all unsuccessful, then we may wonder whether this change will also be ineffective and meaningless. If they were mixed some good, some bad then I will view them with a critical mindset. We may first evaluate internally, then try to adapt. If after adapting we find the change good, we will fully cooperate with it, meet various requirements, continuously learn, so that the change better promotes the hospital and optimises the entire clinical setting. If after evaluation we feel it may not be effective for clinical work or for nursing, then our acceptance will be lower, and the outcomes may not be as good. So we evaluate first, then decide our level of cooperation.

Q: So it depends on whether previous changes were successful. If they were successful, you assume the new change will also have a positive impact and will cooperate. If previous changes were unsuccessful, you wonder whether the new change will be the same increasing workload and pressure. Mainly, you scrutinise and evaluate the change comprehensively. In the process of developing change fatigue, what factors do you think make nurses more likely to feel fatigued when facing changes?

A: I think first, frequency. If changes are too frequent, fatigue will certainly arise. Second, individual adaptability is also important. People with strong adaptability will feel less fatigue; those with weak adaptability may not even accept a small change, so their fatigue will certainly be stronger.

Q: Does the hospital have any measures such as psychological courses or counselling to help nurses relieve change fatigue?

A: Yes. On the annual National Mental Health Day, psychological doctors are invited to the hospital conference room to hold some courses or warm up training. Nursing staff are invited to participate, to express psychological problems, confusion, and obstacles encountered in clinical work, and they provide us with guidance.

Q: Have you attended such a course?

A: Not yet. The times I remember, I was on duty. I saw the information about these courses in the group chat.

Q: So it conflicts with your work schedule, preventing you from attending the lectures.

A: Yes. Usually, these lectures are held on Mental Health Day itself, and staff who are off duty can attend. But if you are on duty, there is a conflict, and you cannot attend.

Q: Is there anything else you would like to add?

A: No.
