# Supplementary material for: Nurses’ experiences of change fatigue during healthcare reforms: a qualitative study of psychological distress, impacts, and adaptation
Source: Front Psychiatry. 2026 Jul 22;17:1878913. doi: 10.3389/fpsyt.2026.1878913 (PMC13437614; doi:10.3389/fpsyt.2026.1878913)
Supplement: Supplementary file 14 [file Table7.docx]

Interview Transcript (N7)

Q: First, please describe one or two change events in which you were actively involved and that left a deep impression on you.

A: Our hospital recently added a new oncology department. I previously worked in orthopaedics, mainly doing orthopaedic surgeries. This new chemotherapy unit is essentially a bone tumour internal medicine department. I had no prior exposure to some of the specialised knowledge in the new department.

Q: When were you transferred to the oncology department?

A: About a month ago. Apart from the unfamiliar specialised knowledge, there were other difficulties. For example, with chemotherapy drugs, I worried about whether they might harm my own health. Although we take various protective measures during operations, because I was not very familiar at the beginning and sometimes very busy, protection might have been inadequate when changing or preparing drugs. Once, after taking off my gloves, there was some drug liquid on my hands. I was very scared. Especially if there were wounds on my hands, I would be even more afraid of the liquid getting in.

Q: You had not been exposed to these chemotherapy drugs before, and suddenly being exposed to them made you feel fearful about the drugs themselves, worrying about potential negative effects on your body.

A: Yes. Besides that, there was also the issue of manpower in the new department. There were already very few nurses. Everyone was transferred from other campuses to support this new department. The number of beds in the new department increased, but the increase in staff was insufficient. The new department has ten beds. If we were only caring for those ten patients, four nurses would be enough. However, because the new department is merged with another department and it is still the beginning, there is no good staffing structure. Only the four of us are in the new department, and we all have relatively low seniority with little say. So there are many difficulties when communicating with the other department. For example, if our department lacks supplies for treatment and needs to borrow from them, the other department's leader feels that using their supplies costs their department, but the performance pay belongs to our department. This creates communication problems.

Q: Does your new medical oncology department not have a head nurse?

A: No. The head nurse from the other department makes our duty schedule. The four of us are in a situation where it seems like every head nurse can manage us, but also like no one really manages us. In principle, we four nurses belong to orthopaedics, but our scheduling and other small matters are managed by the head nurse of the department we are merged with.

Q: So you are managed jointly by two departments, which makes you feel less sense of belonging.

A: Yes. Because the head nurse from the other department makes our schedule, it feels uncomfortable. For example, if I were in my original orthopaedics department, I would communicate directly with the head nurse about which day I want to take off. But under this model in the new department, we rarely request time off. After all, she is not our real leader, so it is hard to communicate many things. And the department is very busy sometimes we need to help the merged department so it is hard to ask for time off. Also, there is the distance issue. After being transferred here, the commute has become much longer. We were all transferred from the main campus, and our rented apartments were near the main campus. I like living close to work because I need to ensure my sleep to maintain a good mood. Now, after transferring from the main campus to the branch campus, the distance has become too far. So in the first few days here, I was very unhappy. When I first arrived, because everything was unfamiliar, I had to work overtime every day. In my previous department, I could basically leave on time, but after transferring to the new department, I had to work at least three or four hours of overtime every day, and my commute increased by more than an hour, so I couldn't rest well at all.Later, I moved to an apartment near the branch campus.

Q: So after the job transfer, you initially had some resistance, right? You said you were very unhappy. After becoming more familiar, how did your attitude change?

A: Yes. Actually, I feel okay about nursing work. The main reason I was unhappy was that I used to live very close to the hospital. After the transfer, the distance became much longer, so I was unhappy. But I had no choice work was already here. So I eventually found a new apartment and moved.

Q: Before your department transfer, did the hospital give any notice for example, a month in advance to allow a buffer period?

A: No. At first, there were rumours, but the hospital officially said nothing. By the time the news came out, it was already very close to the actual transfer date. Then, less than a week before the transfer, I was suddenly told that I would be coming to this new campus, but it was not yet specified which department. It was less than two days before the transfer that I found out which campus and department I would go to. There was so much uncertainty. The news came too suddenly, with no time to think or evaluate. I was forced to accept it and just had to grit my teeth and get through it. It was quite annoying. As an unmarried woman from out of town, I rent an apartment. If the contract is not yet expired and I terminate early, I have to pay a penalty a significant financial loss.

Q: So it was quite sudden, with no time to prepare to do advance work on housing and other aspects in the new environment.

A: Yes, very sudden.

Q: When facing these changes, how did you assess your ability to cope?

A: The news came too suddenly there was no time to think or evaluate. I was forced to accept it and just had to grit my teeth and get through it, solving problems as they arose. For example, with the housing issue, I quickly found an apartment nearby. At work, I continuously learned and adapted. Because the new beds were added in a rather chaotic manner, I kept working overtime, trying to find simpler methods – like copying other people's nursing record templates. Also, for IV preparation, I had not been very exposed to chemotherapy drugs before. Some chemotherapy infusions have strict timing requirements, and there are requirements for the order of drug infusion. Since everyone was newly transferred and not very familiar, our efficiency was very low at the beginning. Later, with time and familiarity, our speed increased, and it felt a bit better.

Q: What were your inner feelings during the learning process?

A: Inner feelings? (Pauses) There was really no time to feel. I just wanted to finish work quickly. All the learning was aimed at working more efficiently so I could leave on time. If overtime is counted, it is somewhat acceptable. But our leaders do not count non‑emergency overtime, so we are unwilling to work extra hours. I just learn in order to finish quickly and leave on time.

Q: So you are emphasising that rest is very important. If you cannot rest properly, your work efficiency may suffer.

A: Yes.

Q: How did learning and coping with these changes affect your psychological state and work state?

A: I am quite easy going and not an overthinker. I do not dwell on these things too much. I learn mainly to finish work early. As long as I can leave on time, as long as the hospital allows me to achieve that goal, I feel that any change is acceptable. I do not feel particularly pressured or overwhelmed.

Q: So for you personally, work changes are not extremely difficult to cope with. Your own ability can handle them, provided your rest is guaranteed.

A: Yes. I value my rest and a happy mood. As long as those two are satisfied, I can learn in various ways to cope with changes whether by improving work speed or adapting to changes and try to meet the hospital's goals. I do not care much about other things; those two are what I focus on.

Q: You have experienced a department transfer, leadership change, and work content adjustment. How do you feel about change in general?

A: I feel that changes are really too frequent. At first, there is a period of difficulty accepting them. Later, after becoming familiar and adapted, it becomes acceptable.

Q: So at the beginning, you found it hard to accept. How did that affect your coping measures and the quality of completing the change requirements?

A: It felt like my motivation gradually faded as I worked. Does that count?

Q: Yes, please elaborate.

A: Regarding quality I care very much about treatment safety. Safety is extremely important in this profession, so it cannot be compromised. For other things, I might not be as meticulous.

Q: So you focus on ensuring safety and avoiding errors, but you do not push yourself to pursue higher work quality.

A: Yes. Actually, there is not much time to demand more of myself, because clinical work alone is already very full.

Q: What thoughts do frequent hospital changes bring to your mind?

A: I feel tired, exhausted. Sometimes I feel emotionally uncomfortable.

Q: How do those states and thoughts affect your work state?

A: When I am at work, my mood is not so pleasant, and I feel that I do not love this job very much.

Q: When you feel unhappy while coping with changes, what measures do you take to relieve it?

A: I call my family, go out with friends to eat, play on my phone, or exercise.

Q: So you use recreational activities for relief. Does the hospital offer any training courses related to these changes? Have you attended any?

A: Yes, there are some. I attended one, but it was an online course. At that time, I was still at the main campus, and there was some training there, but I was on duty, so I attended the courses online.

Q: How many times did you attend online? How did you feel?

A: Only once, I think. It was okay. When I saw the training content, I realised I did not know any of it it felt very difficult. But it was also a way to improve myself, because I did not know the content. However, I learned about it quite late our department was notified last so I only caught one training session. I did not attend others, such as those on protective knowledge, because they were not held again.

Q: Does the hospital organise regular recreational activities to help nurses relieve psychological stress?

A: Yes, but they take up rest time. There have been free clinics, hiking events, and sports meets. I have attended some.

Q: How did you feel after attending?

A: Hiking was okay it is a relaxing activity, getting fresh air. But I prefer to go with my friends people I know so I can enjoy myself. If I go with people I do not know, from other departments, it does not feel relaxing.

Q: If you already feel change fatigue, how does that affect your view of subsequent new changes?

A: As a nurse, I feel that we cannot decide anything about these changes. Whatever the leadership says, we do. It all depends on our own adaptability.

Q: So you still feel forced to accept the situation? When facing a new reform, what thoughts arise?

A: Yes. If another new change comes, I would want to curse I would feel very irritated.

Q: During these hospital changes, what is your view on a stable period?

A: I hope for a stable period giving people some time to adapt is better.

Q: For you personally, how long do you think that stable period should be?

A: I think it depends on the specific situation. For something like learning, it is relatively less hard to accept. I am still young, and my ability to accept new things is okay.

Q: So it depends on the type of change and the specific event, combined with your own learning and adaptability? What factors do you think make nurses more likely to feel fatigued when facing changes?

A: I think first, the leader the leader is very important. Second, the colleagues in the department.

Q: So leadership and team collaboration are the two factors you find important?

A: Yes. Also, commuting distance and rest are very important to me.

Q: Compared with your previous department, has the number of night shifts changed in your new department? Please elaborate.

A: The number of night shifts has increased. Before, there might have been one or two night shifts a week, about six to eight a month. Now it's at least eight, sometimes more.

Q: So after coming to the new department, the number of night shifts has indeed increased.

A: Because there are only four nurses in our new department, the schedule is not very flexible. I can understand with only a few people, if someone needs time off, it may be impossible to arrange the schedule. Someone might have to work continuously, which is quite tiring.

Q: Due to the special nature of the department with insufficient staff, the head nurse cannot arrange the schedule flexibly, so you have to take turns on night shifts.

A: Yes. In other departments, no matter how busy, if you really need time off, you could at least ensure your rest. But here, if you take time off, it means someone else has to work continuously, and the work intensity is already high. So it is very tiring, and you feel embarrassed to request leave.

Q: So due to the complex staffing situation of the new department, your rest time has been greatly reduced?

A: Yes, yes, exactly! The new department is very special.

Q: Regarding nurse change fatigue, is there anything else you would like to add?

A: Also, salary it is gradually decreasing.

Q: Compared with the previous department, or compared among colleagues?

A: Comparing people in the same department. For example, a colleague who joined exactly one year ago, with the same education level, earns less than I did when I had one year of seniority. There is a decrease. Also, the quality of centrally procured drugs is not as good as before. Actually, we are not very happy to accept the centralised procurement because the quality before felt better. For example, with IV catheters, insertion was noticeably smoother than after centralised procurement. Now it is not as smooth, and the products are not as good. Once, when squeezing out a drug, there was something else in it. Before centralised procurement, such things rarely happened. Now, happening two or three times feels very frequent – either something is broken, or a tube is leaking.

Q: So before centralised procurement, such incidents were rare. This adds extra workload to nursing.

A: They did happen, but rarely. After the centralised procurement reform, they became more frequent. In clinical work, for example, when drawing blood from a child, you insert the needle into the vein your technique is fine but the collection tube leaks, and you need to poke again. The parents feel sorry for the child, and we also feel quite speechless.

Q: What were your thoughts when facing these changes?

A: Although it does not feel as good as before, considering it is now a national requirement, I can understand because it is true that accessing medical care is expensive and difficult. I can understand. But the quality has indeed declined. Some centrally procured drugs are also very ineffective they seem to have no effect.

Q: Is that feedback from patients?

A: Yes. Both patients and healthcare staff can feel the quality change. Patients say: "I am willing to pay for my medication, whether expensive or cheap but at least it should work." For antihypertensive drugs, the same drug the imported version effectively lowers blood pressure, but the centrally procured one does not. No matter how cheap, it is useless. Patients are quite unhappy. Moreover, you cannot buy imported drugs in the hospital because the hospital is not allowed to prescribe them only centrally procured ones. If patients want them, they have to buy from pharmacies, but then they cannot get reimbursement very troublesome. Some patients feel that taking centrally procured drugs is pointless they would rather pay more for a truly effective drug.

Q: So you feel that although these changes are nationally advocated and understandable, you still hope for truly meaningful reforms that start from the grassroots level of the hospital.

A: Yes. Whether as nurses or as patients, we all hope for truly meaningful reforms.

Q: Does your hospital implement DRG health insurance payment reform?

A: Yes. The cost difference for surgeries compared with before seems quite large. But we nurses follow doctors' orders I have not paid much attention to this aspect.

Q: So for you personally, your involvement in the health insurance payment reform is not very high?

A: Yes. The surgeons probably know more about it. But after the reform, the checks have indeed become stricter, which is good for patients they save money. However, sometimes the insurance only allows doctors to prescribe a limited amount of medication. Some patients with chronic conditions want to get more at once, which creates conflicts. Patients do not understand why doctors do that, but doctors have no choice the insurance has regulations, and if the prescription exceeds the limit, they may be fined.

Q: How do you view these changes?

A: They need to be viewed dialectically. At the national level, such reforms prevent over treatment. But for patients, especially those with chronic diseases, it is a bit troublesome.

Q: Is there anything else you would like to add?

A: No.

Q: Then this concludes our interview. Thank you for your cooperation.
