# Supplementary material for: Nurses’ experiences of change fatigue during healthcare reforms: a qualitative study of psychological distress, impacts, and adaptation
Source: Front Psychiatry. 2026 Jul 22;17:1878913. doi: 10.3389/fpsyt.2026.1878913 (PMC13437614; doi:10.3389/fpsyt.2026.1878913)
Supplement: Supplementary file 15 [file Table8.docx]

Interview Transcript (N8)

Q: Please describe what types of changes you have experienced in your work, and describe one or two events in which you were highly involved and that left a deep impression on you.

A: First of all, for me over the past three years of work, I think the first change at our hospital is the uncertainty of the workplace. Right now, tertiary hospitals in this city seem to be in a period of expansion constantly expanding into various districts, or even within the province or outside the province, continuously opening new campuses. This means that one hospital has many campuses, which creates great uncertainty for nurses' workplaces. The hospital will irregularly assign you to support different campuses. It is quite different from what we imagined work would be like. Everyone thinks: since I am assigned to this department, to this campus, I should be able to work here steadily. When renting an apartment, you sign a one year lease. Then suddenly one day, the leader tells you that another campus is short of staff and you need to go there for support. The biggest impact on me was last year when the hospital sent me to a faraway city for six months not even in the same city, but to another city.

Q: Did the hospital give you advance notice? For example, a month or even a few days in advance, so you could prepare because it is far away and you would need to find housing.

A: No. Our hospital's personnel arrangements are very rigid, not that humane. I remember it was the 29th, after my night shift. The head nurse came to my ear and said, "Pack your things, you are going to another campus for a while." She told me to leave on the 31st and report to that campus on 1 January. But I was still on a P shift on the 31st. That meant after my late shift, I got home at 10 pm, packed my luggage at 11 pm, and caught a high‑speed train to Shanwei early the next morning at 6 or 7 am to report in.

Q: That is indeed very sudden it is not close. Shanwei is quite far from Guangzhou.

A: Yes. No choice. Our hospital has a campus there, and they were short of staff and needed support.

Q: What were your inner feelings at that time?

A: My first feeling was shock very unbelievable, so sudden. And I thought it would be another campus in the same city, but it turned out to be a completely different city.

Q: The news came too suddenly.

A: Exactly. I felt very surprised. First, the timing was very tight. At that time, 50 of us who joined in the same cohort were suddenly assigned to go.

Q: Did the hospital arrange transport for you, or did you have to arrange your own travel?

A: No transport was arranged. We were told to take the high speed train ourselves.

Q: Did the hospital provide any compensation, such as reimbursing your train fare?

A: Yes, they did. But one thing I think the hospital did poorly in the initial notification was that there was no voluntary principle it was compulsory. The nursing department just issued a list without any survey of employees' opinions. They did not consider things like your rental situation or family relationships. Some people are married or have pets. If you have a pet and have finally found a landlord who allows pets, and then suddenly you are told you have to leave it is very hard to find another landlord who allows pets in such a short time. Regarding family situations, some people have parents who are unwell and brought them to Guangzhou to live together, but then they are suddenly assigned to Shanwei, separating the family again. So the most important issue is that the hospital did not conduct any survey the personnel arrangements were very rigid.

Q: So they do not respect individual wishes.

A: Because the hospital probably feels that if they respected individual wishes, no one would want to go.

Q: That is true it is quite far. How long did you stay there?

A: I stayed for six months from 1 January to 30 June.

Q: Did they tell you how long you would be there when they sent you?

A: They said six months. Actually, we were very scared at the time scared that we might not be able to come back. Although it is the same hospital, we were afraid we might not return.

Q: After staying for six months, how did your attitude change?

A: I adapted to the work there. When I came back, I actually felt a bit not adapted to the original campus. The campus there was newer, colleagues were younger, and the management system was less perfect. Relatively speaking, communication was easier because everyone had similar seniority there was not so much hierarchy. The management system was less strict. When I returned to Guangzhou, I found the management very strict. The head nurse and colleagues were very serious about work, with large gaps in seniority and obvious superior‑subordinate relationships. Also, the patient volume in Guangzhou is huge, and work pressure immediately increased. Not only is the patient volume large, but the severity of illnesses is also higher because Guangzhou receives patients from all over the country. So when I came back, I actually felt a bit not adapted to the original campus.

Q: So during those six months, your workplace kept switching, requiring you to frequently adapt to new work environments.

A: Yes.

Q: Were the departments you worked in and the patient populations you cared for the same as in your original campus?

A: The departments were different. But I am male, and male nurses are usually rotated through fixed departments. My fixed department is emergency, and I rotated through the general ICU. When I went to the new campus, I was in the CCU, which is similar to the cardiac ICU both are critical care. It felt okay. I adapted relatively quickly there because I had already rotated through the general ICU, so rotating through a specialised ICU was much easier.

Q: When you first arrived at the new campus, how did you assess your ability to cope with these changes?

A: I think my adaptability is okay maybe because I am male and more casual. If you are going to be there, the first thing you need to adapt to is your basic living needs – food, clothing, housing, transport. It tests your ability to live independently. The hospital does provide accommodation, but it is a shared dormitory. If you want to live better, you need to rent outside. For fresh graduates, it means jumping from collective university life to individual living from living in a dorm to renting alone and then suddenly from renting alone back to a shared dorm. That can be a bit uncomfortable. For us males, it is still okay. But some female colleagues would tell the nursing department that they are not used to sharing a room with four people. For me, that was fine I could adapt well to living in a shared dorm. Second is the work environment. Although each campus belongs to the same hospital, there are differences between departments such as leave policies and scheduling systems. For example, some departments never had A shift plus N shift, but at the new campus, because of staff shortages, you have to do A shift plus N shift. That totals 16-22 hours quite intense.

Q: Did you work such shifts at your old campus?

A: No. The reason for such scheduling is staff shortage. If they need us for support, it definitely means that campus is very short of staff. They schedule these shifts because they do not have enough people. The A shift ends at 3 pm, then you sleep in between, and come back at 10 pm for the N shift, working until 8 am the next day the intensity doubles compared to before. But it was only occasional if the head nurse scheduled you for such shifts too often, your body could not take it. Another thing to adapt to was that communicating with patients there was very difficult, because the Chaoshan region speaks Teochew dialect. It is really hard. During nursing care, when chatting with patients, we simply could not understand each other they did not understand what I said, and I did not understand what they said. So we had to find someone to translate. When you give them an injection or provide care, they do not understand. Some patients do not want to stay in the ICU because they cannot see their family or get out of bed. At such times, you need to use words to comfort them, but you cannot because of the language barrier. That was a difficulty after the work environment changed.

Q: At your original campus, did you have language barriers when communicating with patients?

A: At the original campus, my language communication was okay. I went to university in Foshan for four years, so I can understand Cantonese, and I can speak it fairly well now after two or three years of work. Speaking of language, there is another internal factor my own initiative to learn and enhance my professional skills. I now work in the emergency department. There is an electronics market near our hospital, so many foreign traders people doing international business come there. Sometimes they feel unwell and come to see a doctor. The first step for them in the emergency department is to register and triage, and there is a language barrier. As you know, there are many foreign traders in Guangzhou from France, Arab countries, Africa, all kinds but the main language is certainly English. When foreigners come to see a doctor, you need to guide them through triage. If your English is not good, communication takes time, causing a long queue behind them, reducing work efficiency. It wastes the foreigners' time, and the local people waiting in line complain: "Why are you so obsequious to foreigners? You talked to them for so long while we waited. Why do you treat foreigners differently from Chinese?" So, to better meet social needs, we usually learn some common medical English phrases for communication.

Q: Is that required by the hospital?

A: No. It is our own initiative to work better, to improve efficiency, and to avoid unnecessary misunderstandings between nurses and patients. I think the best way to prevent change fatigue is to have sufficient personal capability. Therefore, to better adapt to social changes, I usually take the initiative to learn new skillsThat is the main reason an internal change factor. Also, our hospital's Huadu campus was completed this year, and they are again transferring people to support there. Although I have not been chosen yet, it is possible. In summary, the external factor is workplace uncertainty, requiring nurses to constantly face change, accept change, and adapt to change.

Q: Do you worry that you might be the next one to be transferred?

A: Definitely. If I were transferred to Huadu, I have finally rented an apartment with a one year lease. I would have to terminate it early, lose the deposit, and rent again there.

Q: When facing such changes, what thoughts arise?

A: First, I feel particularly insecure. You have already worked here, but you keep being transferred around. Maybe the hospital itself doesn't want to fire you, but if you are transferred too frequently, you feel like quitting yourself.

Q: Because you actually want more stability.

A: Yes. I want more stability. Otherwise, you have to constantly adapt. When you go to a new campus, there is a new leader, new colleagues you have to adapt to those relationships. On one hand, mentally, you have to re-adapt to various systems, leaders, and colleague relationships. On the other hand, materially, you have to spend more money on rent.

Q: So there are negative impacts both economically and psychologically.

A: Yes. Another external pressure I face now comes from changes in the promotion system. Our hospital's promotion system basically updates every few years, and it becomes increasingly strict.

Q: Does promotion refer to moving from staff nurse to nurse practitioner, and then to nurse in charge?

A: Yes, especially from nurse practitioner to nurse in charge. I have not experienced it yet because my seniority is not enough, but my senior colleagues keep telling me: "You need to write papers quickly, otherwise you will be very anxious when you reach our age." Previously, our hospital had a very old promotion system. The requirement for promotion to nurse in charge was simply that you had reached the required seniority, and the hospital would appoint you. Then it changed you needed one patent and one case study to be allowed to apply for promotion. Later, the difficulty increased again it became one paper, one case study, one patent. Now the requirement has increased further you need two papers to be promoted.

Q: Is there a requirement for the quality of the papers must they be in core journals or SCI?

A: No special requirement, but they cannot be too poor. This change in promotion criteria creates pressure. For example, I am only 25 years old now, but I already have to think about promotion issues at age 29 or 30. I might not want to actively think about it, but my senior colleagues tell me: "You need to write papers quickly, otherwise when you actually need to apply for promotion, you will not be able to because you have no papers." But regarding the nursing education system, although we studied courses like nursing research and medical statistics in our undergraduate degree, it was only surface level. To truly apply that knowledge and write papers is still not enough. And for us clinical nurses, work is already very tiring especially in a tertiary hospital in Guangzhou, because the patient volume is huge. After work, I really do not want to move. I used to think about studying for a part‑time master's degree, but now after work I just want to lie down. Work has already consumed most of my energy I am really tired and I do not want to read or study at all.

Q: Regarding this promotion system has it been changing in the past two years, or does a new regulation come out every few years?

A: I think it changes about every four or five years. One of my senior colleagues told me that she wanted to apply for promotion to nurse in charge last year, but because she was not feeling well she had a minor surgery and wanted to rest she did not apply. Then this year, when she went to apply for the promotion, the nursing department's rules had changed. They now require two papers. She has no choice she is now rushing to prepare her second paper.

Q: So before applying, she did not know that the nursing department's regulations had changed. She only found out when she submitted her materials.

A: Yes. The nursing department does not give advance notice that the rules have changed. Only when people who are about to apply for promotion submit their materials do they realise they do not meet the standards. And our hospital's promotion is now very strict you need to meet the required number of night shifts, seniority, number of students taught many requirements.

Q: When these changes bring increased difficulty or adjustments in work content, as you cope with them, what factors do you think are more likely to cause you to feel fatigue?

A: Actually, no matter what factor changes, it all feels quite tiring. I do not like being in a changing environment. Some people might like it, but I prefer stability.

Q: So you do hope for a period of stability, right?

A: Yes.

Q: If there were a stable period, for you personally, how long would you hope it to be?

A: I hope for a stable period, but I have not really thought about how long. I would like it to be stable forever but I know that is impossible, because things are always developing and progressing. If you do not change, you cannot improve. How long? I think at least five or ten years. If policies change too frequently, it makes people very tired.

Q: In the process of coping with changes, how does it affect your psychological state?

A: The main psychological states are anxiety and a sense of uncertainty.

Q: Is the anxiety constant, or does it appear occasionally?

A: How to put it? Work intensity makes me forget the anxiety I am not anxious all the time. When I am busy with work, I forget many things because work requires focus.

Q: When you already feel fatigue while coping with changes, how does that affect your view of subsequent new changes?

A: My view mainly depends on whether my own ability has improved. For example, the reason people feel discouraged by changes in the promotion system is actually that the pace of their ability improvement has not kept up with the pace of the nursing department's rule updates. If your ability becomes very strong, then you do not care how the nursing department's rules change you would not be anxious. Most people's anxiety about this change is because, after long periods of clinical work, they really do not have the energy to do research, so it is hard to produce outputs and hard to meet the hospital's new requirements. Some may have the ability but no energy; others may truly lack the ability. Long-term clinical work indeed leaves no time for study, and they really do not know how to write papers.

Q: When you are stressed, what measures do you usually take to cope and relieve it?

A: As a male, I play games, listen to music, and spend money usually these methods.

Q: Does the hospital organise related activities for example, team outings or psychological courses?

A: Yes, but because work is very tiring, and team activities mean everyone has to be free at the same time on the same day. If you happen to be off that day, you do not actually want to use your own time to participate. I would rather have more freedom and do what I like.

Q: Have you ever participated?

A: No, I have not. I do not want to participate. I do not want to use my rest time to join a group activity it feels meaningless. I would rather sleep more at home. So I do not want to participate. When I am resting, I just want to rest.

Q: When coping with these changes, you do experience negative emotions such as the anxiety you mentioned, because work feels full of uncertainty. How do these emotions affect the quality of your implementation of coping measures?

A: I think the impact on the quality of clinical work is not very great. No matter what, I first need to ensure treatment safety without errors. The work I need to do must still be completed just done in a different place. The changes affect me more internally, such as the need to learn English that improves my ability. Adapting to promotion requirements mainly happens after work. Actually, these changes mainly change my life more I have to take time out of my personal life and put it into work and my profession. I also need to spend more time arranging life matters. These changes increase the annoyances in life time after work needs to be consumed.

Q: Regarding the topic of change fatigue, is there anything else you would like to add?

A: No.

Q: Thank you very much for your cooperation.
