# Supplementary material for: Nurses’ experiences of change fatigue during healthcare reforms: a qualitative study of psychological distress, impacts, and adaptation
Source: Front Psychiatry. 2026 Jul 22;17:1878913. doi: 10.3389/fpsyt.2026.1878913 (PMC13437614; doi:10.3389/fpsyt.2026.1878913)
Supplement: Supplementary file 16 [file Table9.docx]

Interview Transcript (N9)

Q: Please describe one or two change events that left a deep impression on you and in which you were highly involved.

A: When it comes to change, I think performance pay reform is the most prominent a change related to income. It started after 2023, gradually throughout 2023. This has been a major impact, not only in nursing but across many industries. Secondly, with the national development of 5G hospitals and the emergence of various electronic information systems, under the trend of big data and AI intelligence, the change towards electronic work management models is also a major reform for healthcare. These are the reforms I personally feel are large and have had a significant impact on me.

Q: Did these changes cause you pressure or challenges?

A: Both pressure and challenges exist. Performance pay reform has impacted economics and work motivation. The reform of medical models under electronic systems or AI big data has also brought significant changes all of these come with some pressure and challenges.

Q: Under big data and AI driven healthcare, what kind of work are nurses required to do?

A: It depends on the specific context and individual. Some hospitals aim to become 5G hospitals and may place greater emphasis on the use of information technology for example, documentation, record keeping, and recording patients' vital signs become more electronic and intelligent. This brings corresponding changes in data handling. Previously, records were paper based or manually recorded; now there is more intelligent collection and push of data. Many document signatures that used to require handwritten paper signatures are now electronic or even paperless. These are all changes to current work. The specific impact varies by individual and hospital, but the main point is that the era of AI and big data imposes stricter requirements on information and data collection, and makes us more dependent on electronic devices.

Q: When you first learned about these changes, what were your inner feelings?

A: At the beginning, many people especially those with longer work experience and higher seniority felt somewhat uncomfortable in this era of AI and big data. Many operations require computers or mobile terminals and demand proficiency. They may not be skilled at collecting data. In fact, current AI systems are not perfect; they have bugs and drawbacks, and there is a running in period that can be quite long. Many nurses feel that these new things are inconvenient they do not know how to use them and are not familiar with them. For younger nurses or those with higher education, they may see them as modern tools that can facilitate work, because many tedious paper signature processes have been eliminated, replaced by more convenient electronic data collection. The impact on work has both advantages and disadvantages different people have different feelings. Some feel that electronic documentation and data collection have become increasingly detailed, which actually counterproductively adds to their workload. After all, there is a saying: "More work, more mistakes." Is it necessary to collect such redundant and frequent information or write up so much documentation? That is an issue for healthcare management to study and consider. Regarding performance pay reform this is truly about people's livelihood. After the reform, many people's work and life have been greatly impacted. The old idea of a "iron rice bowl" has changed. With the healthcare reform and piece rate or task based compensation, the traditional "big pot rice" and "iron rice bowl" model has been overturned. After the reform, some people's income has decreased, creating a sense of disparity and disappointment. Others may see it positively under the new system, if you want higher pay, you work harder more pay for more work. But some people become passive and slack, feeling that since their salary has dropped, it does not matter how well they work or whether they work at all. Both attitudes exist among healthcare workers.

Q: As the changes gradually progressed, how did your attitude change?

A: At first, I think most people felt resistance more resistance than trying or compliance. Once a fixed mindset is formed, when new things impact you, I think over 70% of people initially lean towards the traditional side. Of course, some choose to embrace and try new things, feeling that change might bring positive new developments. Most people, for various reasons, find the performance management or electronic work model inconvenient at first because the system is not yet mature, or our coping mechanisms are not perfect. Later, gradually, like a frog in slowly heated water, through running in and practice, once you adapt and it imperceptibly integrates into your life, you can accept it and adjust yourself to the environment it is a gradual process of transformation.

Q: In the process of coping with changes, how do you assess your own ability to adapt?

A: I think this is related to one's educational background, age, and years of experience. Young people accept new things faster they are more direct and quicker to embrace and try new things. Moreover, new things are often not entrenched; they represent progressive reform. Social trends move in that direction, and what we can do is to become familiar with the rules and master them. No matter how things change, the most important thing is to understand the rules of the change. When you truly understand the rules, you can comprehend and master the development of the thing. So the ability you need is to understand the change itself, then learn and accept it.

Q: Based on your many years of work experience, what factors make nurses more likely to feel fatigue?

A: Personally, I think performance pay reform brings the greatest impact especially on work motivation, sense of achievement, and satisfaction. As everyone knows, nursing is very hard work with heavy tasks. It requires high concentration and a strong sense of responsibility. Due to the nature of the job, many people experience high work pressure sometimes facing critically ill or challenging patients. If their income level is sharply reduced, normal people will feel unfairness and negativity. Some senior nurses who have been smooth in the workplace may have enjoyed high income for decades. A sudden reduction in performance pay makes them feel that their "iron rice bowl" has been shaken, that their "little cake" has been taken by others. This certainly creates negative emotions when dealing with change. Will these negative emotions affect work? I think the answer is yes. A task that was 90% complete might now only be 70-80% complete. If the job used to pay well, and suddenly the pay is reduced, you work hard every day and then feel: does this income deny your efforts? Or is it a sign of distrust as if the leader thinks you are not busy enough and therefore deducts your pay? People are creatures of belief. When that belief is hit, work motivation is inevitably affected.

Q: Regarding the above, can we summarise that the reward mechanism being misaligned is one factor? What other factors are there?

A: Many have already been mentioned for example, the completeness and rationality of the change, the individual nurse's initiative, the management style and communication skills of the leadership, and also the nurse support system whether it is sufficient to cope with the change, and whether the overall direction suits the current unit or the individual's career development. If the current reform does not match the unit or the nurse's career development plan, it will certainly create negative emotions. For example, if some nurses' career direction is to serve ordinary people at the grassroots level, but the unit's reform is aimed at high-end populations in big cities that clearly does not fit the objective reality of current development. Also, some people have low work motivation to begin with, and some leaders make impulsive decisions. Then there are middle-level leaders who may be inactive, distort the original intention during communication, or alter the objective facts. This creates a series of misunderstandings and conflicts in the transmission and communication process, which greatly affects work motivation and recognition of the reform. Perhaps the higher-level instruction was 123, but by the time it reaches the middle leaders it becomes 23, and by the time it is implemented it becomes 3. This easily leads to misunderstandings about the reform and negative emotions.

Q: So the change itself may not be that bad, but communication problems make people resistant.

A: Yes.Some changes should not be rushed; they should be gradual. Sometimes when implemented too quickly, not everyone has strong adaptability. It takes time for people to become familiar with and master the new rules.

Q: When you already feel fatigue from coping with changes, how does that affect your view of subsequent new changes?

A: Sometimes constant changes do cause fatigue and burnout. But because of my years of experience, I still look forward to new changes and developments. Things cannot remain unchanged; they are constantly in motion and development. Whether a new change suits the current situation can only be determined through actual implementation after all, practice is the sole criterion for truth. Whether a new change promotes the existing work model and methods you only know after trying it. If it is bad, you can discard it; if it is good, you can embrace it.

Q: So you feel that maybe the next new change could bring something good "what if the next one is good"?

A: Yes. Looking at the development of healthcare and nursing, there have been many changes, and some have indeed been good, promoting the industry's progress. For example, previously we needed large paper medical records; now with various electronic systems, it is indeed much more convenient. Previously, diagnosis relied entirely on human judgement; now with AI diagnostic assistance, the error rate has truly decreased. For things that are uncertain, machines can assist in diagnosis that is an objective fact and truly helpful. Not all changes are beneficial; only through practice can we know whether a change promotes development.

Q: Before the hospital implements changes, are there any support measures such as pre-job training or courses targeting nurses' psychological emotions to relieve anxiety and fatigue?

A: There are very few psychological measures. Training certainly exists for example, on how to use AI and technology assisted medical diagnosis. Those courses are definitely offered, because if you are going to be exposed to new things, they will provide course training to explain what the new thing is. But when it comes to every small detail, there may be gaps.

Q: When you feel stressed or have negative emotions at work, what methods do you usually use to relieve them?

A: Personally, I think rest is a very good way to relieve stress staying away from work for a period,keeping distance from your unit, completely relaxing, and devoting yourself to a period of time that belongs to you. You can go on holiday to a place far from your unit. During those days, completely detach yourself from the unit. I think this is a very good way to relax.

Q: Does the hospital provide any related activities to help employees relax physically and mentally?

A: Every year, the trade union organises spring and autumn outings tourist activities. But there are not many humanistic care activities. Mainly, they are group activities organised by the trade union.

Q: During frequent hospital changes, when you already feel fatigue from coping, what are your views or impacts on subsequent changes?

A: You feel that money is hard to earn, and life is hard. But this is reality no choice. You have to go along with the tide of development. Whether you accept it actively or passively, you will ultimately have to accept it. So you might as well relax, accept the change, try to accept the positive parts. But I think the bad parts should also be opposed you cannot accept everything indiscriminately. People should have their own sense of logic and subjective awareness. If it is good, I accept it; if it is bad, I raise my opinion. That is the correct way to cope with and accept change. You cannot blindly accept everything you need to analyse change dialectically with a critical mindset.

Q: In the process of coping with changes, how do you view a stable period?

A: A stable period is certainly necessary. Ultimately, all reforms will have a stable period including performance pay reform. At first, people may resist strongly. When you realise you cannot change the situation, you have to subjectively accept the change. When you accept it, that is essentially the arrival of the stable period.

Q: For you personally, how long would that stable period be? How do you estimate that time?

A: I think the average time for a person to accept something new is about one to three months. It takes 21 days to form a habit. Fast adapters may accept the change within a month. Of course, there are individual exceptions who may take several years or never accept it. For a normal person, a few months is enough to accept the change, because once they become familiar with it, they accept it.

Q: So it depends on the individual's acceptance and adaptability.

A: Yes.

Q: Regarding nurse change fatigue, what aspects do you think researchers should pay more attention to?

A: First, I think we need to understand why change is happening. Let me approach this from three perspectives. First, from the manager's perspective: we need to understand why managers want to change what is the meaning of the change? Where do we start the change? Change cannot detach from the grassroots foundation or detach from objective reality. Does our change have any pretentious and empty elements? This requires thinking based on practice and down to earth reality it is also a question that leaders and managers need to consider: the scientific basis and necessity of the change. Second, from the middle-level managers (the implementers): their methods of communicating the change, communication skills, and collection of feedback on the outcomes of the change- can they achieve a coherent process? Middle-level managers should not only implement without collecting feedback. Only through feedback can we know how well the group accepts the change. Finally, for us as the subjects of the change: we need to dialectically accept, try, and embrace the change not blindly accept it all. Is the change good? Does it truly optimise our work? If it is good, I do not think people would feel negative or burnout, because it is a good thing. If it is bad and has a negative impact on me, creating negative emotions, then I think it is necessary to report this feedback. But whether my feedback is useful and can be accepted that involves the communication feedback from upper leadership. I think forming a communication loop among the three levels upper, middle, and lower is a very important factor for the effective implementation of reform.

Q: Thank you very much for answering my questions in such detail. Regarding nurse change fatigue, is there anything else you would like to add?

A: Not for now.

Q: Thank you for your answers.
